# Red–Blue LED Light and Plasma-Activated Water Enhance Vindoline and Catharanthine Yields in *Catharanthus roseus* Grown in a Closed Plant Production System

**DOI:** 10.3390/plants15192970

**Published:** 2026-09-29

**Authors:** Alessandro Quadri, Mattia Trenta, Bianca Sambuco, Patrizia Tassinari, Daniele Torreggiani, Assunta Bertaccini, Laura Mercolini, Michele Protti, Francesco Tomelleri, Romolo Laurita, Alberto Barbaresi

**Affiliations:** 1 Department of Agricultural and Food Sciences (DISTAL), University of Bologna, Viale Fanin, 44, 40127 Bologna, Italy; mattia.trenta3@unibo.it (M.T.); alberto.barbaresi@unibo.it (A.B.); 2 Department of Pharmacy and Biotechnology (FABIT), University of Bologna, Via Belmeloro, 6, 40126 Bologna, Italy; 3 Department of Industrial Engineering (DIN), University of Bologna, Viale Risorgimento, 2, 40136 Bologna, Italy

**Keywords:** controlled environment agriculture (CEA), indoor vertical farming, LED light, plasma-activated water, medicinal and aromatic plants (MAPs), alkaloids, vindoline (VDL) and catharanthine (CAT), *Catharanthus roseus*

## Abstract

Madagascar periwinkle [*Catharanthus roseus* (L.) G. Don] produces vindoline and catharanthine, key precursors used in the semi-synthesis of the high-value anticancer alkaloids vinblastine and vincristine. Closed plant production systems (CPPS) allow precise application of environmental *stimuli*, including light spectrum and plasma-activated water (PAW). In a previous study, we demonstrated the potential of combining artificial light (white and red) and PAW to enhance the production of these alkaloids in periwinkle grown under controlled CPPS conditions. The present study expands this approach by adopting a more scalable PAW application method and a broader range of LED light spectra to evaluate their effects on vindoline and catharanthine accumulation. After a 37-day pre-treatment under white LEDs in environmentally controlled rooms, plants were subjected to fourteen treatments: phosphor-converted warm white (WW-P, control), red (R), blue (B), red–blue (RB), red–blue–far red (RBFR), phosphor-converted cool white (CW-P), and color-mixed white (W-CM) LED light, each with or without PAW. Plant growth and alkaloid-related responses were assessed at three sampling times: before treatment application (T0), 47 (T1), and 55 (T2) days after pre-treatment. RB produced the highest total vindoline and catharanthine yields [sum of leaf and root yields, with each organ yield calculated as organ dry mass (DM, g plant^−1^) × organ alkaloid concentration (µg g^−1^ DM)], reaching 228.0 ± 16.9 and 338.0 ± 27.0 µg plant^−1^, respectively, which were significantly higher than those obtained under all other light spectra (1.4-fold for vindoline and 1.3-fold for catharanthine relative to WW-P), except for W-CM, for which no significant differences were observed. PAW significantly increased total vindoline and catharanthine yields, reaching 211.0 ± 8.0 and 278.0 ± 11.0 µg plant^−1^, respectively, corresponding to 1.9- and 1.3-fold increases compared with the untreated condition. Sampling time had a strong effect on total vindoline and catharanthine yields, increasing from 6.0 ± 0.7 and 13.9 ± 1.7 µg plant^−1^, respectively, at T0, to 258.7 ± 9.8 and 414.0 ± 17.0 µg plant^−1^, respectively, at T2, corresponding to 43.1- and 29.8-fold increases. In conclusion, the combined use of RB light and PAW, together with harvesting at T2, represented the most effective strategy to achieve high vindoline and catharanthine production, as it enhanced the total yields of both alkaloids.

## 1. Introduction

A closed plant production system (CPPS), including plant factories with artificial lighting (PFALs) and their small-scale counterparts, such as environmentally controlled growth rooms, enables strict control over environmental conditions for plant cultivation [1,2]. This ensures stable production regardless of weather conditions and allows the cultivation of clean vegetables. However, to operate an enclosed artificial-lighting plant factory economically, it is essential to focus on high-value crops, such as medicinal and aromatic plants (MAPs) [3] that permit high profitability.

Among MAPs, Madagascar periwinkle [*Catharanthus roseus* (L.) G. Don.] is a studied and commercially relevant species due to its ability to synthesize terpenoid indole alkaloids (TIAs) [4]. These compounds include the dimeric alkaloids vinblastine (VBL) and vincristine (VCR), two cornerstone anticancer drugs used for the treatment of various hematological and solid cancers [5]. Structural analogue derivatives such as vinorelbine, vindesine, and vinflunine have subsequently been developed and also hold considerable therapeutic importance for cancer treatment [6]. However, the exclusive presence of VBL and VCR in Madagascar periwinkle leaves, together with their extremely low concentrations in dry plant leaves (0.01% for VBL and 0.0003% for VCR [7]) and their complex chemical structures, results in difficult and expensive purification and extraction processes [8], with consequent market prices of millions of dollars per kilogram [8,9]. These challenges have therefore stimulated interest in strategies to enhance the biosynthesis of the monomeric precursors of these compounds, namely vindoline (VDL) and catharanthine (CAT), which occur in relatively higher concentrations in the plant. These compounds can be used as substrates for the semi-synthesis of VBL and VCR through enzymatic or non-enzymatic coupling processes [10].

The production of Madagascar periwinkle in open-field conditions is strongly influenced by climatic variability, which can hinder both the continuity and the quality of alkaloid supply [11]. In Mediterranean regions, its annual growth habit further restricts outdoor cultivation during the cold season [12]. Consequently, growing Madagascar periwinkle in a CPPS represents a promising strategy to guarantee year-round production of biomass rich in valuable alkaloids such as VDL and CAT. In addition to providing stable production conditions, CPPSs also offer a highly controllable experimental platform for the precise manipulation of environmental *stimuli*, thereby enabling targeted optimization of both plant growth and specialized metabolite (SM) accumulation [13].

Among environmental *stimuli*, light plays a crucial role in regulating SM biosynthesis [14]. Recent advances in LED lighting technology have enabled the design of highly specific lighting “recipes” with optimized spectra, exposure durations, and intensities [15,16], able to enhance the production of plant SMs, including TIAs [17,18,19]. In particular, several studies have shown that LED light spectral composition, particularly in the red (R) and red–blue (RB) ranges, can significantly influence the accumulation of VDL and CAT in Madagascar periwinkle [20,21,22,23,24,25,26]. Although modulation of light quality has demonstrated potential as an effective strategy to enhance TIA biosynthesis, additional approaches are required to further increase their accumulation.

In this context, plasma-activated water (PAW) has emerged as another promising environmental *stimulus*. PAW employment demonstrated efficacy as a plant growth promoter and resistance inducer, and it can also be used in combination with LED lighting [26,27,28,29]. PAW is a solution enriched in reactive oxygen and nitrogen species (RONS), generated by exposing water to a plasma source [30,31]. These molecules are involved in plant signalling pathways regulating stress responses and specialized metabolism [31].

Several studies have demonstrated that RONS can induce defence-like responses in plant tissues, thereby stimulating SM biosynthesis in different plant species [32,33], including TIAs in Madagascar periwinkle [25,26], suggesting its potential as a complementary tool to LED-based strategies.

In a previous study [25], we investigated the combined effects of artificial light and PAW on Madagascar periwinkle cultivated under controlled indoor conditions. In that study, artificial lighting consisted of white light (W) provided by fluorescent lamps and R light provided by LEDs, and the response to PAW was evaluated as VDL and CAT concentrations in leaves. The study provided an initial proof of concept that PAW could enhance the accumulation of these TIA precursors, particularly under R light, and demonstrated that the plant response to PAW could depend on the light environment. However, the experimental scope was limited to two light conditions and to foliar alkaloid concentrations, leaving unresolved how PAW responsiveness varies across a broader range of light spectra and whether changes in alkaloid concentration are associated with changes in overall alkaloid production.

The present study was designed to address these limitations by substantially expanding the evaluation of the LED–PAW combination. Seven LED spectral configurations were investigated, encompassing monochromatic red and blue spectra, red–blue and red–blue–far-red combinations, and different white-light configurations, each applied with and without PAW. This broader spectral range allows the Madagascar periwinkle response to PAW to be evaluated across different light environments and provides a more comprehensive assessment of the role of spectral composition in modulating plant growth and alkaloid production. Thus, rather than simply extending the number of light treatments, the present experimental design addresses a broader biological question: whether the effects of PAW on VDL and CAT production are dependent on the spectral environment in which plants are cultivated.

A further important extension concerns the distinction between alkaloid concentration and alkaloid yield [i.e., alkaloid concentration × dry mass (DM) of the corresponding organ]. The previous study [25] focused on VDL and CAT concentrations in leaves, whereas the present study considers both biomass accumulation and alkaloid concentration. Leaf and root dry masses (DMs) are determined separately, together with total plant DM, while VDL and CAT concentrations are quantified in both leaves and roots. These measurements are then integrated to calculate VDL and CAT yields in leaves, roots, and the whole plant. This approach is important because concentration alone does not necessarily reflect the amount of alkaloid produced by the plant: changes in concentration may occur independently of, or in parallel with, changes in biomass accumulation. By integrating these two components, the present study provides a more comprehensive assessment of the production response to LED spectrum and PAW.

The inclusion of roots also extends the analytical scope of the previous work, which focused exclusively on leaves. Evaluating VDL and CAT in both leaves and roots enables the response of different plant organs to LED spectrum and PAW to be characterized and allows their respective contributions to whole-plant alkaloid production to be considered. This organ-resolved approach is particularly relevant for Madagascar periwinkle, given the complex spatial organization of TIA metabolism and the established importance of inter-organ processes in the formation and distribution of these alkaloids. Accordingly, assessing both organs provides a broader framework for interpreting the effects of environmental *stimuli* on TIA production than measurements restricted to foliar tissues.

The temporal dimension was also expanded relative to the previous work [25]. Whereas the previous experiment included two sampling times during the treatment period, the present study includes three sampling times, comprising a pre-treatment baseline and two subsequent experimental sampling times. This additional temporal characterization allows changes in biomass, alkaloid concentrations, and alkaloid yields to be followed over the course of the experiment and provides a more complete assessment of the temporal dynamics of the response to LED spectrum and PAW.

In parallel with this expanded biological and analytical scope, the present study introduces a new, scaled-up, and more operationally relevant PAW production and application strategy. In the previous study [25], PAW was generated from sterile distilled water (SDW) using a corona-discharge plasma system and applied by briefly immersing individual potted plants upside down into PAW-filled jars. This more laboratory-oriented procedure was intentionally designed as a controlled approach to evaluate the physiological response of Madagascar periwinkle to PAW. However, the use of SDW, the need to manually handle individual potted plants, and the production capacity of up to 0.5 L of PAW per cycle limited the practicality of this procedure for the treatment of larger plant batches. In the present study, PAW was instead generated from tap water using a hybrid corona-discharge/dielectric barrier discharge (DBD) plasma system, with a production capacity of up to 6 L per cycle, and applied by foliar spraying using a battery-powered backpack atomizer commonly used for agricultural treatments. These modifications are intended to improve the operational applicability and potential scalability of PAW treatment under controlled-environment cultivation conditions, thereby facilitating its application to larger plant batches.

To complement the evaluation of treatment effects, a *Logarithmic Mean Divisia Index* (LMDI)-based decomposition analysis was applied to quantify the relative contributions of biomass and alkaloid concentration to changes in VDL and CAT yields. This analytical approach makes it possible to distinguish changes associated with biomass accumulation from those associated with changes in alkaloid concentration and, at the whole-plant level, to evaluate the respective contributions of leaves and roots. Consequently, the present study is designed not only to assess whether the LED spectrum and PAW modify alkaloid yield, but also to provide a quantitative framework for evaluating the relative contributions of biomass and alkaloid concentration to changes in productivity.

Therefore, the principal objective of the present study is to move beyond the previous proof-of-concept assessment of LED–PAW effects on foliar VDL and CAT concentrations by providing an integrated evaluation of how a broader range of LED spectral environments, with and without PAW, affects biomass accumulation and VDL and CAT production at the organ and whole-plant levels. By considering biomass, alkaloid concentration, alkaloid yield, organ-specific responses, and changes over time within a single experimental framework, the study provides a more comprehensive framework for elucidating how LED spectral composition and PAW influence the production of valuable anticancer TIA precursors in Madagascar periwinkle cultivated in a CPPS.

## 2. Results

### 2.1. Plasma Discharge Performance and PAW Properties

#### 2.1.1. Electrical Performance of Plasma Sources

The DBD configuration exhibited an average discharge power of 250.4 ± 29.3 W, while the corona discharge operated at 82.2 ± 12.1 W. The overall energy density imparted by the hybrid system was estimated at 0.01 kWh·L^−1^.

#### 2.1.2. pH of PAW

**Figure 1:**
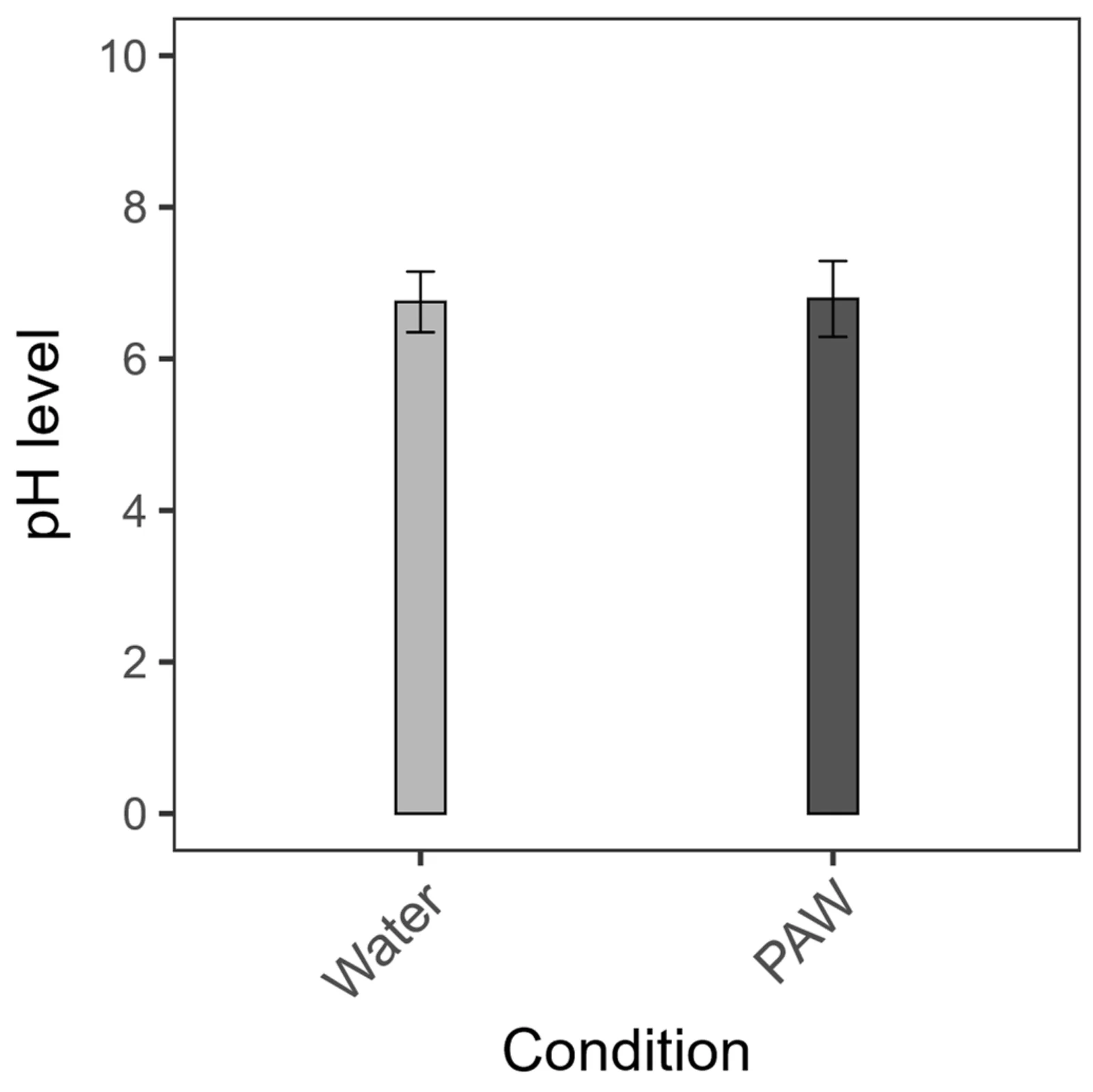
Comparison of pH values between untreated water and PAW.

The pH of PAW was comparable to that of untreated water, with only a minor difference between the two mean values (Figure 1).

#### 2.1.3. RONS Content in PAW

**Figure 2:**
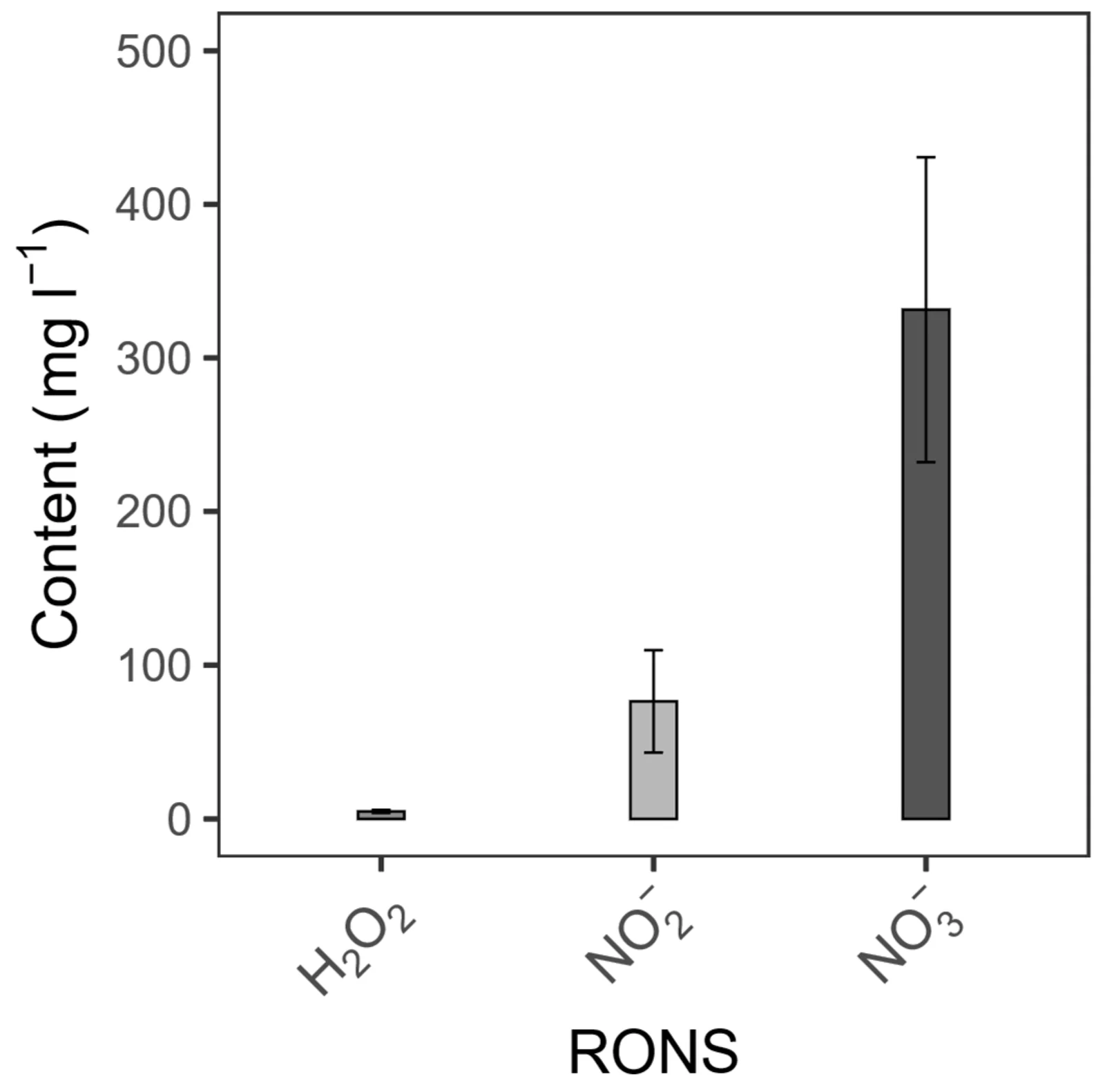
Contents of H_2_O_2_, NO_2_^−^, and NO_3_^−^ in PAW after treatment.

Plasma exposure induced the formation of RONS in water, including hydrogen peroxide (H_2_O_2_), nitrite (NO_2_^−^), and nitrate (NO_3_^−^) (Figure 2). These contents remained stable for at least 4 h when samples were stored at room temperature.

### 2.2. ANOVA Results

Table 1 presents the results of the three-way ANOVA assessing the effects of LED light spectrum, PAW, and sampling time on DM, as well as on the concentrations and yields of VDL and CAT in Madagascar periwinkle. Regarding DMs, leaf, root, and total DM were significantly affected by LED light spectrum, PAW, and sampling time, with a significant PAW:sampling time interaction observed only for total DM.

For alkaloid concentrations, VDL was significantly influenced by all three factors, with two significant interactions detected: PAW:sampling time for leaf VDL and LED light spectrum:sampling time for root VDL. CAT concentrations were significantly affected by LED light spectrum and sampling time, while PAW had a significant effect only on leaf CAT. No significant interactions were detected for either leaf or root CAT.

Regarding alkaloid yields, both VDL and CAT were significantly affected by the LED light spectrum, PAW, and sampling time across all plant compartments (leaf, root, and total). In particular, leaf and total yields of both alkaloids were significantly influenced by all three main factors, with no significant interaction effects detected. A similar pattern was observed for root VDL yield, which was also significantly affected by all three factors without significant interactions. In contrast, root CAT yield showed not only significant effects of the LED light spectrum, PAW, and sampling time, but also a significant PAW:sampling time interaction.

The significant interactions identified in this study all involved sampling time, namely PAW:sampling time and LED light spectrum:sampling time (codings for their combinations are reported in detail in Supplementary Materials, Table S1). Although we are aware that, when significant two-way interactions are present (particularly when at least one of the corresponding main factors is also significant), only the interaction effect should typically be analyzed and discussed to avoid potentially misleading interpretations, we nonetheless decided to examine and discuss the effect of the sampling time. This was done to evaluate temporal variations in a more isolated manner and to highlight general trends that may not be fully evident when considering the interaction alone. While the interaction provides information on the combined influence of the LED light spectrum or PAW and sampling time, the effect of the sampling time offers a clearer view of the temporal dynamic patterns, illustrating how, on a general level, the variable changes over time independently of LED light spectrum or PAW.

Furthermore, the decision to analyze the effect of sampling time was motivated by the need to compare the temporal trends of variables showing significant interactions with those for which only the effect of sampling time was significant, and no time-related interactions were detected. This approach enabled a more direct and coherent comparison of temporal variations across variables that share similar time-driven patterns but differ in their interaction structure.

### 2.3. Treatment Effects on Plant DMs

#### 2.3.1. Leaf DM

##### LED Light Spectrum Effects

**Figure 3:**
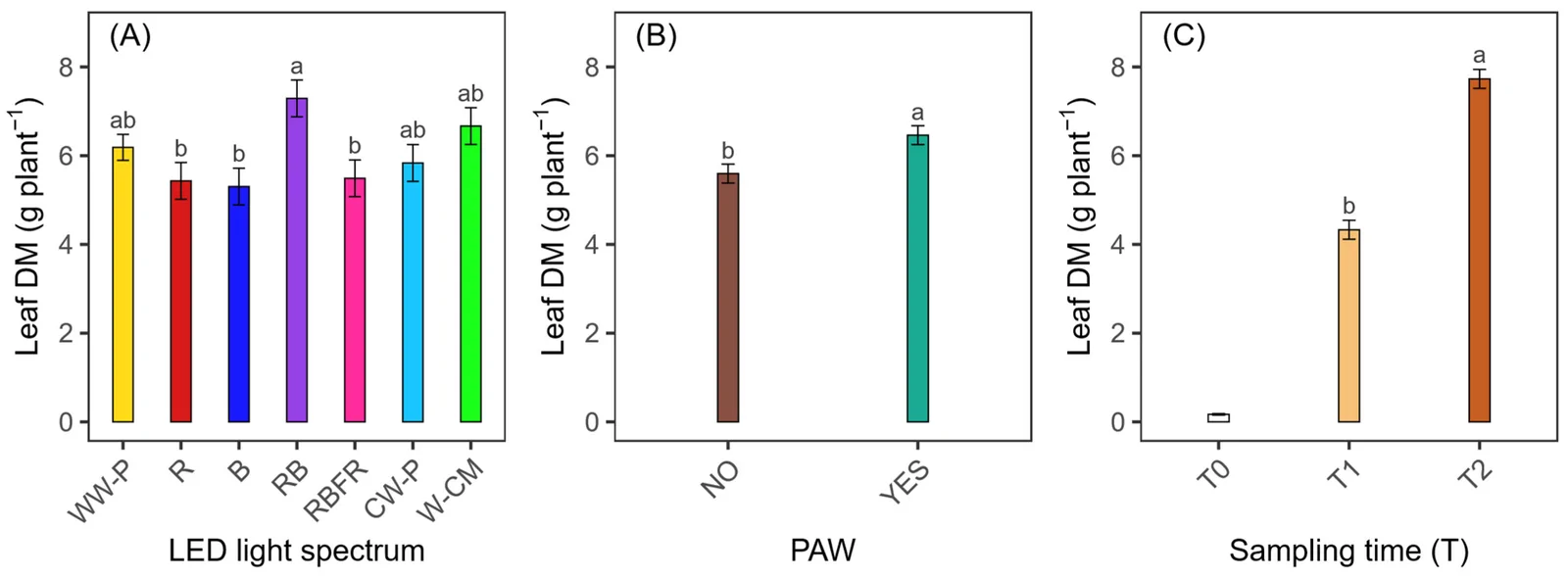
Treatment effects on leaf dry mass (DM) in Madagascar periwinkle. (**A**) Light spectrum (WW-P, R, B, RB, RBFR, CW-P, and W-CM). (**B**) PAW (NO = absence of PAW treatment; YES = presence of PAW treatment). (**C**) Sampling time (T: T1 = 47 DAP, T2 = 55 DAP); T0 (1 DAP) represents the reference baseline condition prior to the application of any experimental treatment and is labelled in this form for graphical and terminological consistency within the sampling time effect structure; therefore, T0 is not part of the post-hoc comparisons. Bars represent SE (*n* = 5). Different letters indicate significant differences (*p* ≤ 0.05) according to Tukey’s method with Benjamini–Hochberg adjustment (LED light spectrum) or *t*-tests (PAW and sampling time).

Figure 3A displays the effects of LED light spectrum on leaf DM. RB light treatment resulted in the highest leaf DM, while the lowest was observed under B light. In particular, RB light produced a significantly higher DM only compared to R, B, and RBFR light (1.3-, 1.4-, and 1.3-fold higher, respectively), while showing no significant differences relative to the other spectra.

##### PAW Effects

Plants receiving PAW showed a clear increase in leaf DM, with values significantly exceeding those of the untreated condition (1.2-fold higher; Figure 3B).

##### Sampling Time Effects

Leaf DM increased markedly over time (Figure 3C), showing a progressive accumulation pattern. Values at T1 were substantially (25.5-fold) higher than at T0 and increased significantly further to levels 1.7-fold higher at T2 compared to T1. Overall, leaf DM at T2 was dramatically (43.1-fold) higher than at T0.

#### 2.3.2. Root DM

##### LED Light Spectrum Effects

**Figure 4:**
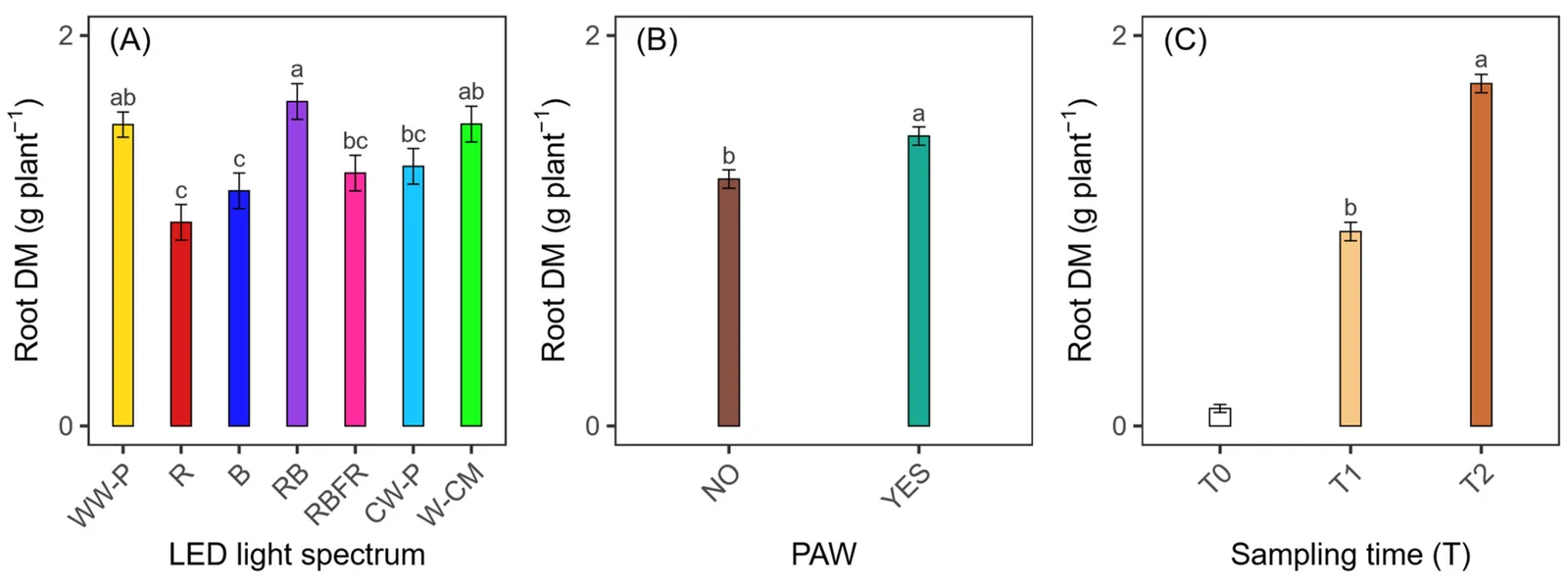
Treatment effects on root dry mass (DM) in Madagascar periwinkle. (**A**) Light spectrum (WW-P, R, B, RB, RBFR, CW-P, and W-CM). (**B**) PAW (NO = absence of PAW treatment; YES = presence of PAW treatment). (**C**) Sampling time (T: T1 = 47 DAP, T2 = 55 DAP); T0 (1 DAP) represents the reference baseline condition prior to the application of any experimental treatment and is labelled in this form for graphical and terminological consistency within the sampling time effect structure; therefore, T0 is not part of the post-hoc comparisons. Bars represent SE (*n* = 5). Different letters indicate significant differences (*p* ≤ 0.05) based on Tukey’s method with Benjamini–Hochberg adjustment (LED light spectrum) or *t*-tests (PAW and sampling time).

The impact of LED spectral conditions on root DM is depicted in Figure 4A. Maximum root DM was observed under RB light, whereas the lowest values were recorded under R light. In detail, RB light promoted significantly higher root DM compared to R, B, RBFR, and CW-P treatments (1.6-, 1.4-, 1.3-, and 1.2-fold higher, respectively), while no significant differences were detected relative to WW-P and W-CM.

##### PAW Effects

Root DM responded positively to PAW application, with a significant increase (1.2-fold higher) compared to the untreated condition (Figure 4B).

##### Sampling Time Effects

A strong temporal effect was also evident for root DM (Figure 4C), with biomass progressively increasing across sampling times. In particular, root DM at T1 was substantially (11.1-fold) higher than at T0, followed by a further significant increase of 1.8-fold at T2 relative to T1. Over the entire experimental period, root DM at T2 was dramatically (19.4-fold) higher than at T0.

#### 2.3.3. Total DM

##### LED Light Spectrum Effects

**Figure 5:**
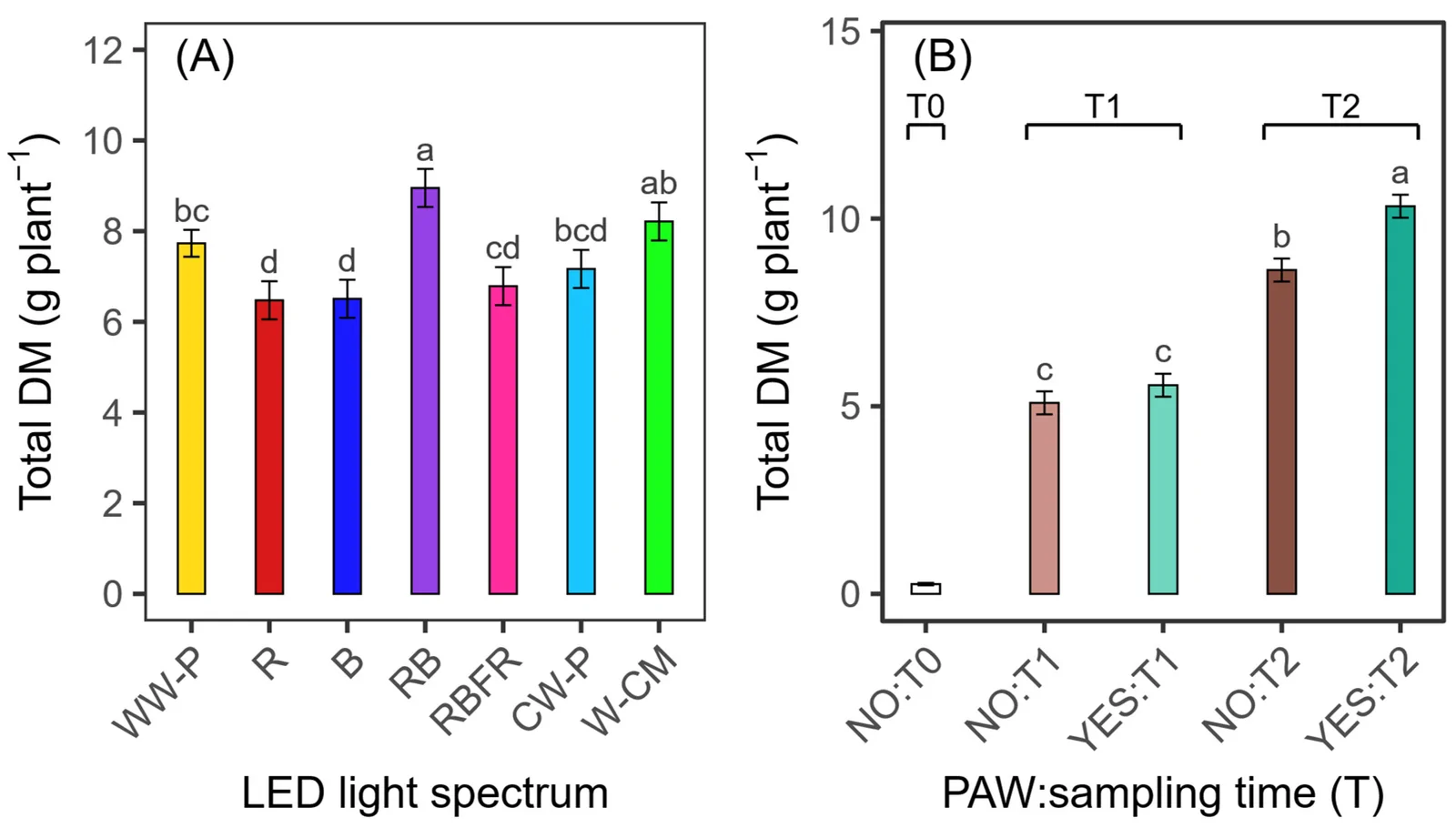
Treatment effects on total dry mass (DM) in Madagascar periwinkle. (**A**) LED light spectrum (WW-P, R, B, RB, RBFR, CW-P, and W-CM). (**B**) All combinations of PAW (NO = absence of PAW treatment; YES = presence of PAW treatment) and sampling time (T: T1 = 47 DAP, T2 = 55 DAP); NO:T0 denotes the reference baseline condition (T0 sampling, 1 DAP) prior to the application of any experimental treatment and is labelled in this form for graphical and terminological consistency within the PAW:sampling time (T) interaction structure; therefore, NO:T0 is not part of the post-hoc comparisons. Bars represent SE (*n* = 5). Different letters indicate significant differences (*p* ≤ 0.05) according to Tukey’s method with Benjamini–Hochberg adjustment.

LED light spectrum effect on total DM is shown in Figure 5A. The highest values were measured under RB light (8.95 ± 0.42 g plant^−1^), whereas plants grown under R light exhibited the lowest total DM (6.47 ± 0.42 g plant^−1^). RB treatment resulted in significantly greater total biomass compared to all other spectra (e.g., 1.2-fold higher than WW-P), except for W-CM, with which it did not differ statistically.

##### Sampling Time Effects

Total DM increased markedly over time (Figure S1), showing a clear accumulation trend. Values at T1 were substantially higher (20.5-fold) than at T0, followed by a further significant increase of 1.8-fold at T2 relative to T1. Across the entire sampling period, total DM increased markedly (36.5-fold higher).

##### Combined Effects of PAW and Sampling Time

Contrary to leaf and root DM, the effect of PAW on total DM depended on the harvest period (Figure 5B). Overall, the highest total DM was observed under PAW-treated plants harvested at T2 (YES:T2, 10.33 ± 0.31 g plant^−1^), while the lowest was observed in untreated plants harvested at T1 (NO:T1, 5.09 ± 0.31 g plant^−1^). When also considering the T0 baseline (NO:T0), the lowest absolute DM occurred prior to treatment application (0.26 ± 0.07 g plant^−1^). Specifically, YES:T2 plants exhibited a significantly higher total DM than the other treatment combinations (approximately 2.0- and 1.2-fold higher compared with NO:T1 and NO:T2, respectively) and markedly higher values relative to the pre-treatment baseline (NO:T0).

PAW treatment produced higher total DM only under equal sampling-time conditions: at T1, PAW-treated plants (YES:T1) showed slightly greater (though not statistically significant) total DM compared with untreated plants (NO:T1), whereas at T2, PAW-treated plants (YES:T2) exhibited significantly higher (1.2-fold) total DM than NO:T2; however, total DM under NO:T2 was significantly higher than in YES:T1.

Moreover, total DM followed a temporal pattern consistent with that observed for both leaf and root DM, exhibiting a substantial cumulative increase across sampling times, in line with the overall trend highlighted for the effect of sampling time alone. Indeed, both treatment combinations at T1 (NO:T1 and YES:T1) showed substantially higher values than the T0 baseline (NO:T0), with increases of 19.6- and 21.4-fold, respectively; both treatment combinations at T2 (YES:T2 and NO:T2) exhibited significantly higher total DM than those at T1. Overall, all treatments at T1 (NO:T1 and YES:T1) and T2 (NO:T2 and YES:T2) produced markedly higher total DM than NO:T0, confirming a strong cumulative effect over time.

### 2.4. Treatment Effects on VDL and CAT Concentrations

#### 2.4.1. Leaf Concentrations

##### LED Light Spectrum Effects

**Figure 6:**
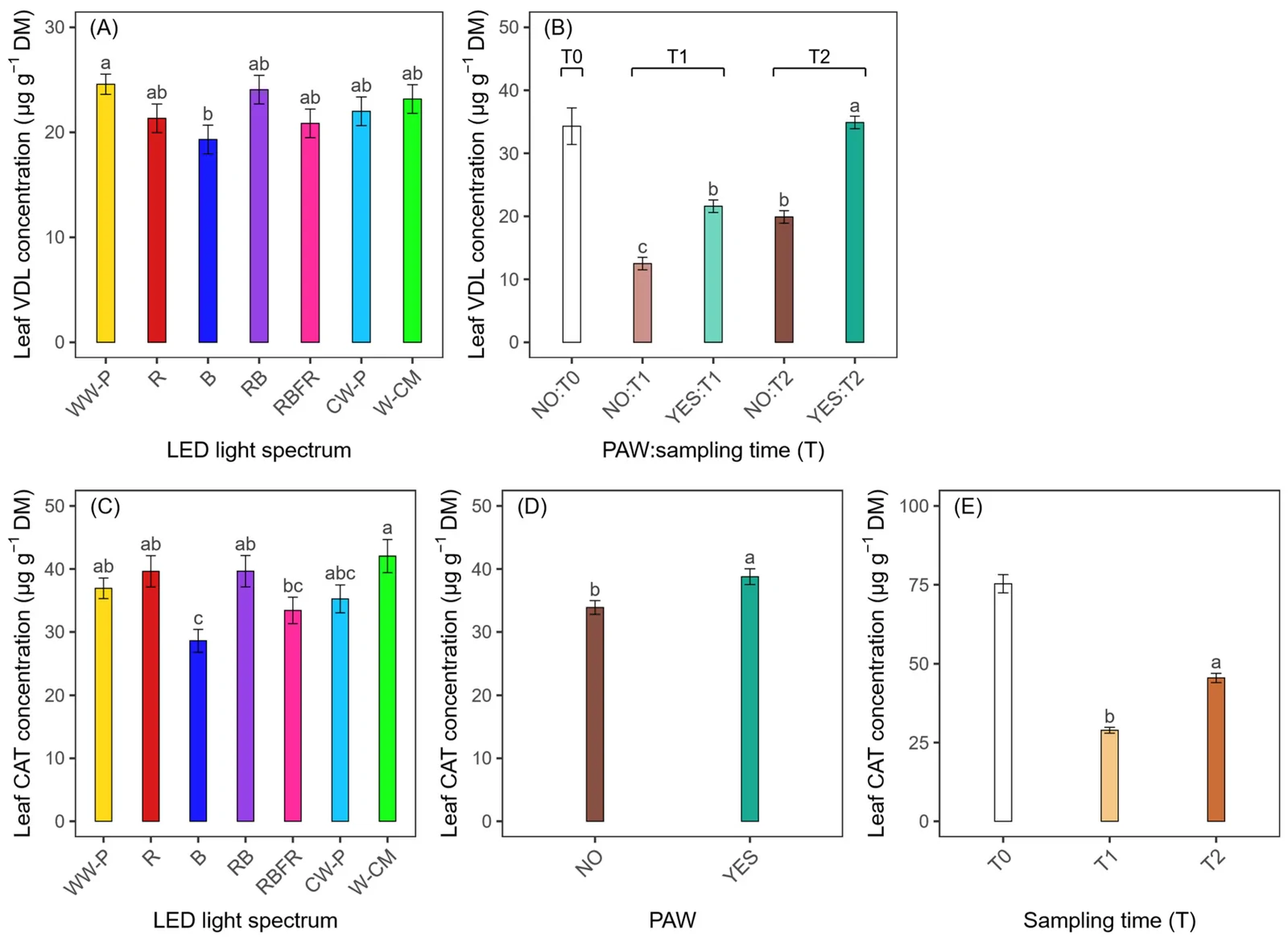
Treatment effects on leaf vindoline (VDL) and catharanthine (CAT) concentrations in Madagascar periwinkle. (**A**,**C**) LED light spectrum (WW-P, R, B, RB, RBFR, CW-P, and W-CM) on VDL and CAT, respectively. (**B**) All combinations of PAW (NO = absence of PAW treatment; YES = presence of PAW treatment) and sampling time (T: T1 = 47 DAP, T2 = 55 DAP) on VDL; NO:T0 denotes the reference baseline condition (T0 sampling, 1 DAP) prior to the application of any experimental treatment and is labelled in this form for graphical and terminological consistency within the PAW:sampling time (T) interaction structure; therefore, NO:T0 is not part of the post-hoc comparisons. (**D**) PAW on CAT. (**E**) Sampling time on CAT; T0 (1 DAP) represents the pre-treatment baseline condition and is coded in this form for graphical and terminological consistency within the sampling time effect structure; therefore, T0 is not part of the post-hoc comparisons. Bars represent SE (*n* = 5). Different letters indicate significant differences (*p* ≤ 0.05) based on Tukey’s method with Benjamini–Hochberg adjustment (LED light spectrum, PAW:sampling time interaction) or *t*-tests (PAW and sampling time).

Figure 6A,C illustrates the effect of LED light spectrum on leaf VDL and CAT concentrations, respectively. In general, WW-P and W-CM light produced the highest leaf VDL and CAT contents (24.6 ± 1.0 µg g^−1^ DM and 42.0 ± 2.6 µg g^−1^ DM, respectively, while B resulted in the lowest levels (19.3 ± 1.4 µg g^−1^ DM and 28.6 ± 1.8 µg g^−1^ DM, respectively). RB light produced the second-highest leaf concentrations of both VDL (24.1 ± 1.4 µg g^−1^ DM) and CAT (39.6 ± 2.5 µg g^−1^ DM).

Specifically, for VDL, WW-P light resulted in a significantly higher leaf concentration only compared to B light (1.3-fold higher), while being statistically comparable to R, RB, RBFR, CW-P, and W-CM; no significant differences were also observed among R, B, RB, RBFR, CW-P, and W-CM treatments. For CAT, W-CM light produced a significantly higher concentration compared to B and RBFR (1.5- and 1.3-fold higher, respectively), while being statistically comparable to WW-P, R, RB, and CW-P. WW-P, R, and RB light also resulted in significantly higher CAT concentrations compared to B (1.4-fold higher), with no significant differences among them or relative to RBFR, WW-P, and CW-P. Notably, CAT concentration under B light was significantly lower than under all other spectra, except CW-P and RBFR, with which it was statistically comparable.

##### PAW Effects

Leaf CAT concentration was significantly higher in PAW-treated plants than in untreated plants (Figure 6D), with a 1.1-fold increase.

##### Sampling Time Effects

Although the effect of sampling time alone on leaf VDL concentration was not significant according to the ANOVA, its analysis (Figure S2) revealed a non-linear temporal pattern characterized by an initial decline followed by partial recovery. Specifically, VDL concentration was highest at T0 (34.3 ± 2.9 µg g^−1^ DM), decreased markedly at T1 (0.5-fold relative to T0), where the minimum value was observed (17.0 ± 0.7 µg g^−1^ DM), and then increased significantly at T2 (27.4 ± 0.7 µg g^−1^ DM; 1.6-fold higher than T1). However, VDL levels at T2 remained substantially lower than those at T0 (0.8-fold of T0 levels).

A similar non-linear trend was observed for leaf CAT concentration (Figure 6E). CAT levels were highest at T0 (75.3 ± 2.9 µg g^−1^ DM), decreased substantially at T1 (0.6-fold relative to T0), reaching minimum values (28.9 ± 0.9 µg g^−1^ DM), and then increased significantly at T2 (45.5 ± 1.5 µg g^−1^ DM; 1.6-fold higher than T1). Nevertheless, even at T2, CAT concentration remained markedly lower than at T0 (0.4-fold relative to T0).

##### Combined Effects of PAW and Sampling Time

Leaf VDL concentration exhibited a clear interaction between PAW treatment and sampling time (Figure 6B). Across the sampling period, VDL levels exhibited a pattern consistent with that observed for the effect of sampling time alone, namely a non-linear trend characterized by an initial decrease followed by recovery. At T0, VDL concentration was high (34.3 ± 2.9 µg g^−1^ DM), followed by a substantial decline at T1 (NO:T1 −12.5 ± 1.0 µg g^−1^ DM; YES:T1 −21.6 ± 1.0 µg g^−1^ DM). At T2, VDL levels increased again, showing partial recovery under NO:T2 (19.9 ± 1.0 µg g^−1^ DM) and reaching the highest value under the YES:T2 combination (34.9 ± 1.0 µg g^−1^ DM).

Comparing all different treatment combinations (excluding the pre-treatment baseline NO:T0), YES:T2 resulted in significantly higher VDL concentration than the others (e.g., 2.8- and 1.8-fold higher than NO:T1 and NO:T2, respectively).

In addition, PAW application consistently promoted higher VDL concentrations, as evidenced by the YES:T1 combination, which showed a significantly higher value than NO:T1 (1.7-fold higher) and remained higher, although not significantly, than NO:T2.

#### 2.4.2. Root Concentrations

##### LED Light Spectrum Effects

**Figure 7:**
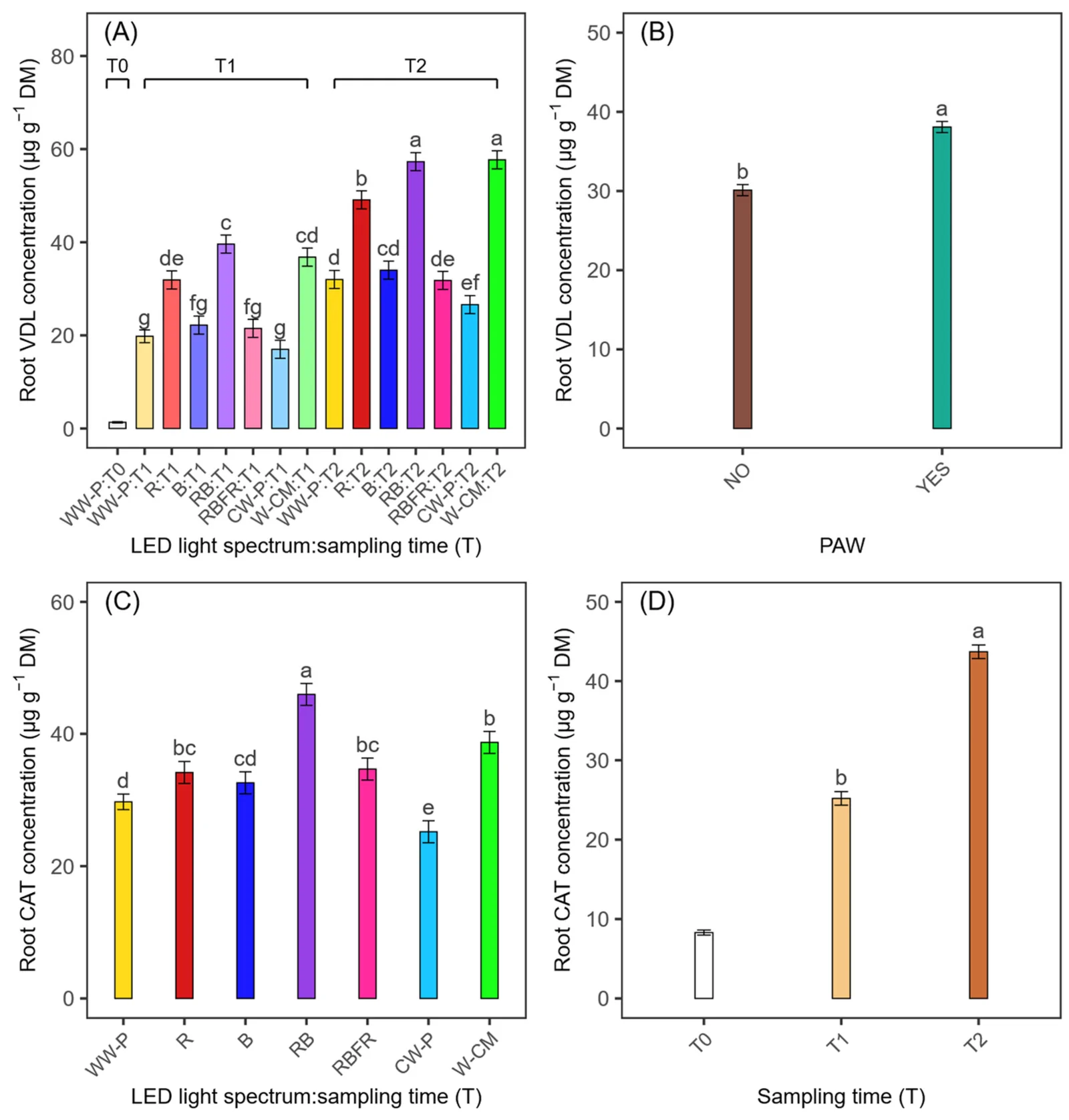
Treatment effects on root vindoline (VDL) and catharanthine (CAT) concentrations in Madagascar periwinkle. (**A**) All combinations of LED light spectrum (WW-P, R, B, RB, RBFR, CW-P, and W-CM) and sampling time (T: T1 = 47 DAP, T2 = 55 DAP) on VDL; WW-P:T0 denotes the reference baseline condition prior to the application of any experimental treatment (T0 sampling, 1 DAP) and is labelled in this form for graphical and terminological consistency within the LED light spectrum:sampling time (T) interaction; therefore, WW-P:T0 is not part of the post-hoc comparisons. (**B**) PAW (NO = absence of PAW treatment; YES = presence of PAW treatment) on VDL. (**C**) LED light spectrum on CAT concentration. (**D**) Sampling time on CAT; T0 (1 DAP) represents the pre-treatment baseline condition and is coded in this form for graphical and terminological consistency within the sampling time effect structure; therefore, T0 is not part of the post-hoc comparisons. Bars represent SE (*n* = 5). Different letters indicate significant differences (*p* ≤ 0.05) based on Tukey’s method with Benjamini–Hochberg adjustment (LED light spectrum, LED light spectrum:sampling time interaction) or *t*-tests (PAW and sampling time).

Figure 7C depicts the effect of LED light spectrum on root CAT concentration. In general, the highest root CAT levels were observed under RB light (46.0 ± 1.7 µg g^−1^ DM), while the lowest were observed under CW-P light (25.2 ± 1.7 µg g^−1^ DM). W-CM produced the second-highest root CAT levels (42.0 ± 2.6 µg g^−1^ DM). In particular, RB light produced a significantly higher root CAT concentration compared to all the other light spectra (e.g., 1.5-fold higher relative to WW-P). Similarly, W-CM also resulted in a significantly higher root CAT concentration compared to WW-P and CW-P, while remaining statistically comparable to that of B, R, and RBFR.

##### PAW Effects

PAW produced a significant effect only on VDL (Figure 7B). Specifically, PAW-treated plants exhibited a significantly higher root VDL concentration compared to untreated plants (1.7-fold higher).

##### Sampling Time Effects

Contrary to what was observed for leaf VDL and CAT concentrations, the analysis of the effect of sampling time alone (Figure S3) reveals a strong non-linear increasing trend in root VDL concentration from T0 to T2. Root VDL content was highest at T2 (41.2 ± 0.7 µg g^−1^ DM) and lowest at T0 (1.3 ± 0.7 µg g^−1^ DM). From T0 to T1, root VDL content markedly increased by 20.5-fold. From T1 to T2, the effect of sampling time showed a significant increase of 1.5-fold. Overall, from T0 to T2, root VDL levels increased by 31.2-fold.

Similarly, root CAT concentration showed a notable, approximately linear increasing pattern across the different time points (Figure 7D). In particular the CAT concentration was the lowest at T0 (8.3 ± 0.3 µg g^−1^ DM), increased notably at T1 (3.6-fold higher), and then increased significantly at T2 (1.7-fold higher), reaching the maximum value (43.7 ± 0.9 µg g^−1^ DM), with an overall increase of 5.3-fold from T0 to T2. Moreover, in this study, we confirmed the findings reported by Quadri et al. [24], namely that VDL is not only present in the roots, but that its average concentration exceeds that in the leaves (Figure S4), in contrast to what has been previously reported for Madagascar periwinkle by several other authors.

##### Combined Effects of LED Light Spectrum and Sampling Time

Figure 7A illustrates the effect of the interaction between LED light spectrum and sampling time on root VDL. The highest root VDL concentration was observed in plants treated with W-CM light and sampled at T2 (W-CM:T2 −57.7 ± 1.9 µg g^−1^ DM), whereas the lowest concentrations were recorded exposed to CW-P light and sampled at T1 (CW-P:T1 −17.0 ± 1.9 µg g^−1^ DM) and, in term of absolute values, in those sampled at the pre-treatment baseline condition (WW-P:T0 −1.3 ± 0.1 µg g^−1^ DM). Plants exposed to RB light and sampled at T2 (RB:T2) also exhibited particularly high root VDL levels, representing the second highest values after W-CM:T2 (57.3 ± 1.9 µg g^−1^ DM). Specifically, the W-CM:T2 and RB:T2 combinations resulted in significantly higher root VDL concentrations than all other treatment combinations. Both W-CM:T2 and RB:T2 showed approximately 2.9- and 1.8-fold higher values compared with WW-P-treated plants sampled at T1 (WW-P:T1) and T2 (WW-P:T2), respectively. The W-CM:T2 and RB:T2 treatments were statistically comparable and both exhibited dramatically higher root VDL concentrations relative to the pre-treatment baseline WW-P:T0 (44.4- and 44.0-fold higher, respectively).

Furthermore, at T1, plants treated with RB light (RB:T1) displayed the highest root VDL concentration, followed by W-CM plants (W-CM:T1). In particular, RB:T1 resulted in a significantly higher concentration than all other combinations (e.g., two-fold higher compared to WW-P:T1), except for W-CM:T1, with which it was statistically comparable.

Temporal analysis across combinations indicates that root VDL concentration is strongly governed by the interaction between light spectrum and sampling time. Indeed, only within the same light spectrum was root VDL concentration significantly higher at T2 than at T1. Notably, some treatment combinations at T1 [RB:T1, W-CM:T1 and R:T1 (plants treated with R light and sampled at T1)] resulted in higher root VDL concentrations than certain combinations at T2 (e.g., RBFR:T2, CW-P:T2), indicating that the effect of light spectrum was not consistent over time and that the interaction between light spectrum and sampling time plays a key role in regulating this variable.

### 2.5. Treatment Effects on VDL and CAT Yields

#### 2.5.1. Leaf Yields

##### LED Light Spectrum Effects

**Figure 8:**
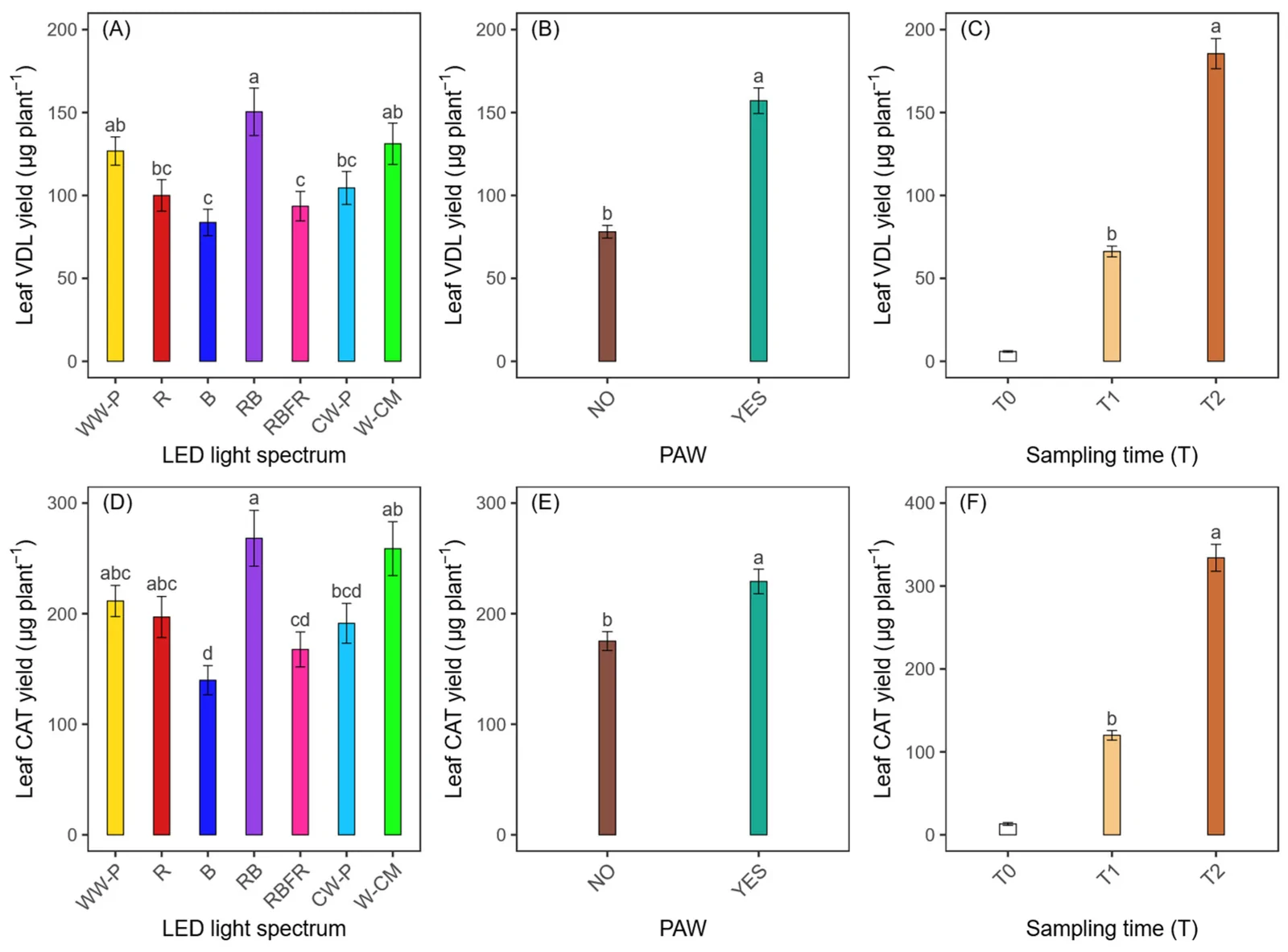
Treatment effects on leaf vindoline (VDL) and catharanthine (CAT) yields in Madagascar periwinkle. (**A**,**D**) LED light spectrum (WW-P, R, B, RB, RBFR, CW-P, and W-CM) on VDL and CAT, respectively. (**B**,**E**) PAW (NO = absence of PAW treatment; YES = presence of PAW treatment) on VDL and CAT, respectively. (**C**,**F**) Sampling time (T: T1 = 47 DAP, T2 = 55 DAP) on VDL and CAT, respectively; T0 (1 DAP) represents the reference baseline condition prior to the application of any experimental treatment and is labelled in this form for graphical and terminological consistency within the sampling time effect structure; therefore, T0 is not part of the post-hoc comparisons. Bars represent SE (*n* = 5). Different letters indicate significant differences (*p* ≤ 0.05) based on Tukey’s method with Benjamini–Hochberg adjustment (LED light spectrum) or *t*-tests (PAW and sampling time).

The influence of LED light quality on leaf VDL and CAT yields is shown in Figure 8A,D. Overall, RB light resulted in the highest yields for both alkaloids (VDL: 150.4 ± 14.3 µg plant^−1^; CAT: 268.0 ± 25.3 µg plant^−1^), while the lowest values were recorded under B light (VDL: 83.7 ± 8.0 µg plant^−1^; CAT: 140.0 ± 13.2 µg plant^−1^). W-CM light also supported relatively high yields, ranking second for both VDL (131.1 ± 12.5 µg plant^−1^) and CAT (259.0 ± 24.4 µg plant^−1^).

Specifically, for VDL, RB light significantly increased leaf VDL yield compared to all the other light spectra (e.g., 1.8- and 1–5-fold higher relative to B and R light, respectively), except for W-CM and WW-P, with which it was statistically comparable; W-CM light showed a significantly higher leaf VDL yield only compared to RBFR and B light. For CAT, plants grown under RB light exhibited significantly higher leaf yields than those under B (1.9-fold), RBFR (1.6-fold), and CW-P (1.4-fold), while remaining statistically comparable to WW-P, W-CM, and R. In addition, W-CM light resulted in significantly higher CAT yields only compared with RBFR and B light.

##### PAW Effects

PAW-treated plants showed significantly higher yields of both VDL (Figure 8B) and CAT (Figure 8E) compared with untreated plants, with increases of 2-fold for VDL and 1.3-fold for CAT.

##### Sampling Time Effects

A non-linear temporal pattern was observed for both leaf VDL and CAT yields across sampling times (Figure 8C,F). Both alkaloids increased markedly over time, with minimum values observed at T0 (VDL: 5.9 ± 0.5 µg plant^−1^; CAT: 13.1 ± 1.7 µg plant^−1^) and maximum values at T2 (VDL: 185.5 ± 9.1 µg plant^−1^; CAT: 334.0 ± 16.2 µg plant^−1^). The increase was particularly pronounced between T0 and T1 (11.2-fold for VDL and 9.2-fold for CAT), followed by a further significant rise from T1 to T2 (2.8-fold for both alkaloids). Overall, leaf VDL and CAT yields at T2 were 31.4-fold and 25.5-fold higher than at T0, respectively.

#### 2.5.2. Root Yields

##### LED Light Spectrum Effects

**Figure 9:**
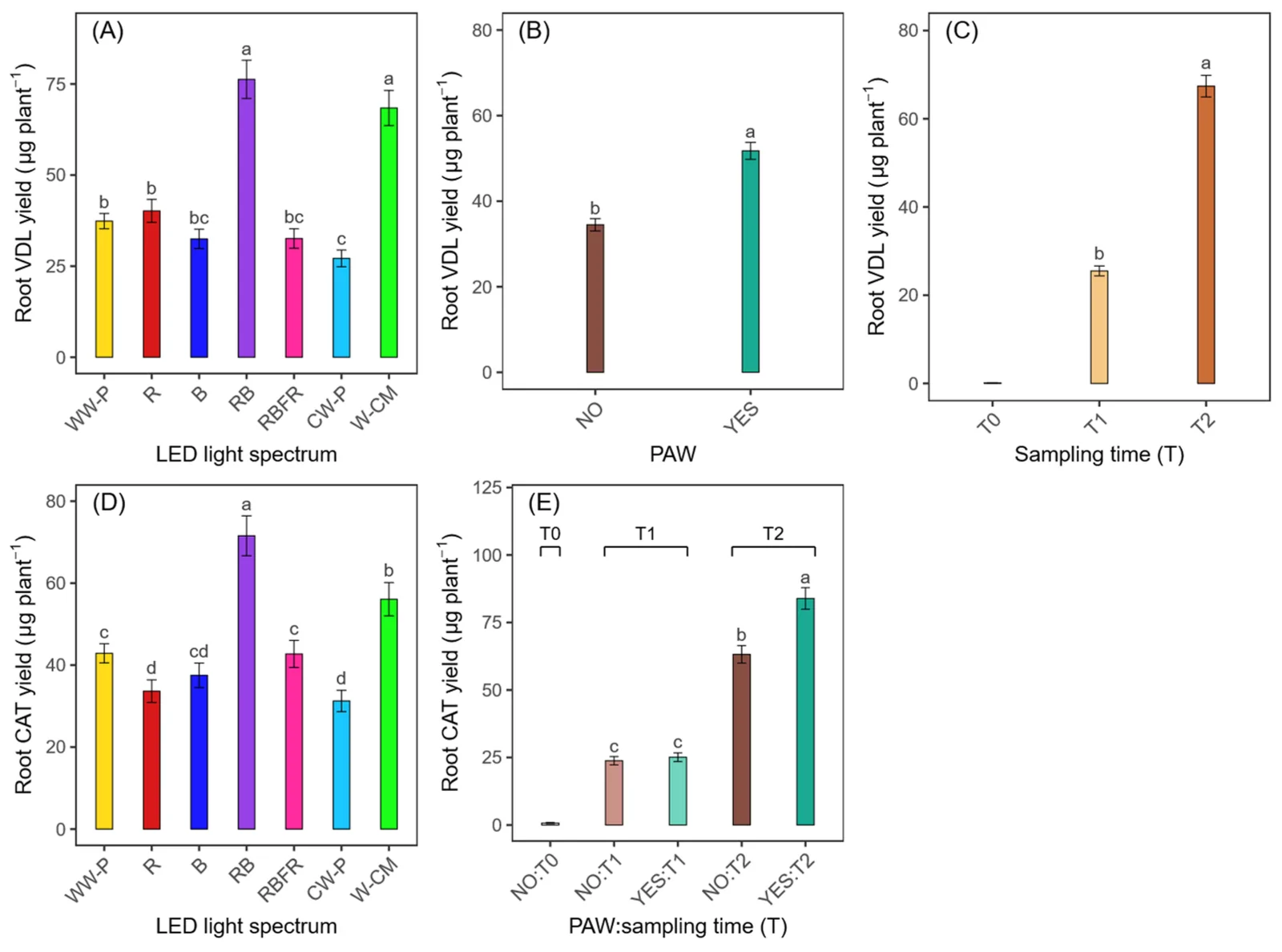
Treatment effects on root vindoline (VDL) and catharanthine (CAT) yields in Madagascar periwinkle. (**A**,**D**) LED light spectrum (WW-P, R, B, RB, RBFR, CW-P, and W-CM) on VDL and CAT, respectively. (**B**) PAW (NO = absence of PAW treatment; YES = presence of PAW treatment) on VDL. (**C**) Sampling time (T: T1 = 47 DAP, T2 = 55 DAP) on VDL yield; T0 (1 DAP) represents the reference baseline condition prior to the application of any experimental treatment and is labelled in this form for graphical and terminological consistency within the sampling time effect structure; therefore, T0 is not part of the post-hoc comparisons. (**E**) All combinations of PAW and sampling time (T) on CAT; NO:T0 denotes the pre-treatment reference baseline condition (T0 sampling, 1 DAP) and is labelled in this form for graphical and terminological consistency within the PAW:sampling time (T) interaction structure; therefore, NO:T0 is not part of the post-hoc comparisons. Bars represent SE (*n* = 5). Different letters indicate significant differences (*p* ≤ 0.05) based on Tukey’s method with Benjamini–Hochberg adjustment (LED light spectrum, PAW:sampling time interaction) or *t*-tests (PAW and sampling time).

The effect of LED spectral conditions on root VDL and CAT yields is shown in Figure 9A,D. Overall, the yields of both alkaloids peaked under RB light (VDL: 76.3 ± 5.3 µg plant^−1^; CAT: 71.6 ± 4.9 µg plant^−1^, respectively), whereas the lowest values occurred under CW-P light (VDL: 27.1 ± 2.3 µg plant^−1^; CAT: 31.3 ± 2.6 µg plant^−1^, respectively). W-CM light again produced comparatively high yields, ranking second for both VDL (68.4 ± 4.8 µg plant^−1^) and CAT (56.1 ± 4.1 µg plant^−1^). Specifically, for VDL, RB light resulted in a significantly higher root VDL yield than all the other light spectra (e.g., 2.0-fold higher relative to WW-P), except for W-CM, with which it was statistically comparable. W-CM light showed a significantly higher root VDL yield compared to all the other spectra, except RB. For CAT, RB-grown plants exhibited significantly higher root yields than those under all the other light spectra (e.g., 1.7-fold higher compared to WW-P). Likewise, W-CM light led to a significantly higher CAT yield compared to that of all the other tested spectra, except that of WW-P, with which no significant differences were observed.

##### PAW Effects

Root alkaloid yields also responded positively to PAW application. Notably, PAW-treated plants showed significantly higher yields of both VDL (Figure 9B) and CAT (Figure 9E) compared with untreated plants, with values being 1.5-fold and 1.2-fold higher, respectively.

##### Sampling Time Effects

Figure 9C and Figure S5 show the temporal dynamics of root VDL and CAT yields, which exhibited a pronounced non-linear response over time, characterized by a sharp increase at early stages followed by a more gradual rise thereafter. Both alkaloid yields increased markedly from T0 to T2, reaching minimum levels at T0 (VDL: 0.1 ± 0.07 µg plant^−1^; CAT: 0.7 ± 0.2 µg plant^−1^) and maximum values at T2 (VDL: 67.4 ± 2.5 µg plant^−1^; CAT: 73.0 ± 2.5 µg plant^−1^). The increase was particularly steep between T0 and T1, where yields were substantially higher than at T0 (255.0-fold for VDL and 34.9-fold for CAT), followed by a further significant rise up to T2, with values 2.6-fold and 3.0-fold higher than at T1 for VDL and CAT, respectively. Overall, from T0 to T2, root VDL and CAT yields dramatically increased by 674- and 104.3-fold, respectively.

##### Combined Effects of PAW and Sampling Time

The effect of PAW treatment on root CAT yield was actually dependent on sampling time (Figure 9E). On a general level, the highest root CAT yield was observed under the YES:T2 combination (83.9 ± 4.0 µg plant^−1^), while the lowest was under NO:T1 (23.8 ± 1.5 µg plant^−1^). Including T0, the minimum absolute value was for plants harvested before treatment application (NO:T0, 0.7 ± 0.2 µg plant^−1^). YES:T2 plants exhibited significantly higher root CAT yields than all the other post-treatment combinations (e.g., 3.5- and 1.3-fold higher, respectively, compared to NO:T1 and NO:T2). Analyzing PAW effects across all treatment combinations, PAW-treated plants showed higher root CAT yield only under equal sampling-time conditions. At T1 (YES:T1), values were slightly higher (though not statistically significant) than in untreated plants (NO:T1), whereas at T2 (YES:T2), they were significantly higher (1.3-fold) than in NO:T2. However, root CAT yield under NO:T2 remained significantly higher than under YES:T1. Furthermore, root CAT yield increased markedly across sampling times, consistent with the general trend associated with the effect of sampling time alone. Specifically, at T1 both treatment combinations (NO:T1 and YES:T1) exhibited markedly higher values than the T0 baseline (NO:T0) with 34.0- and 35.9-fold increases, respectively; similarly, at T2 both treatment combinations (YES:T2 and NO:T2) highlighted a significantly higher root CAT yield than those observed at T1. Overall, all treatments at both T1 and T2 (NO:T1, YES:T1, NO:T2, and YES:T2) resulted in a dramatically higher root CAT yield compared to the pre-treatment baseline (NO:T0), showing a strong cumulative temporal effect.

#### 2.5.3. Total Yields

##### LED Light Spectrum Effects

**Figure 10:**
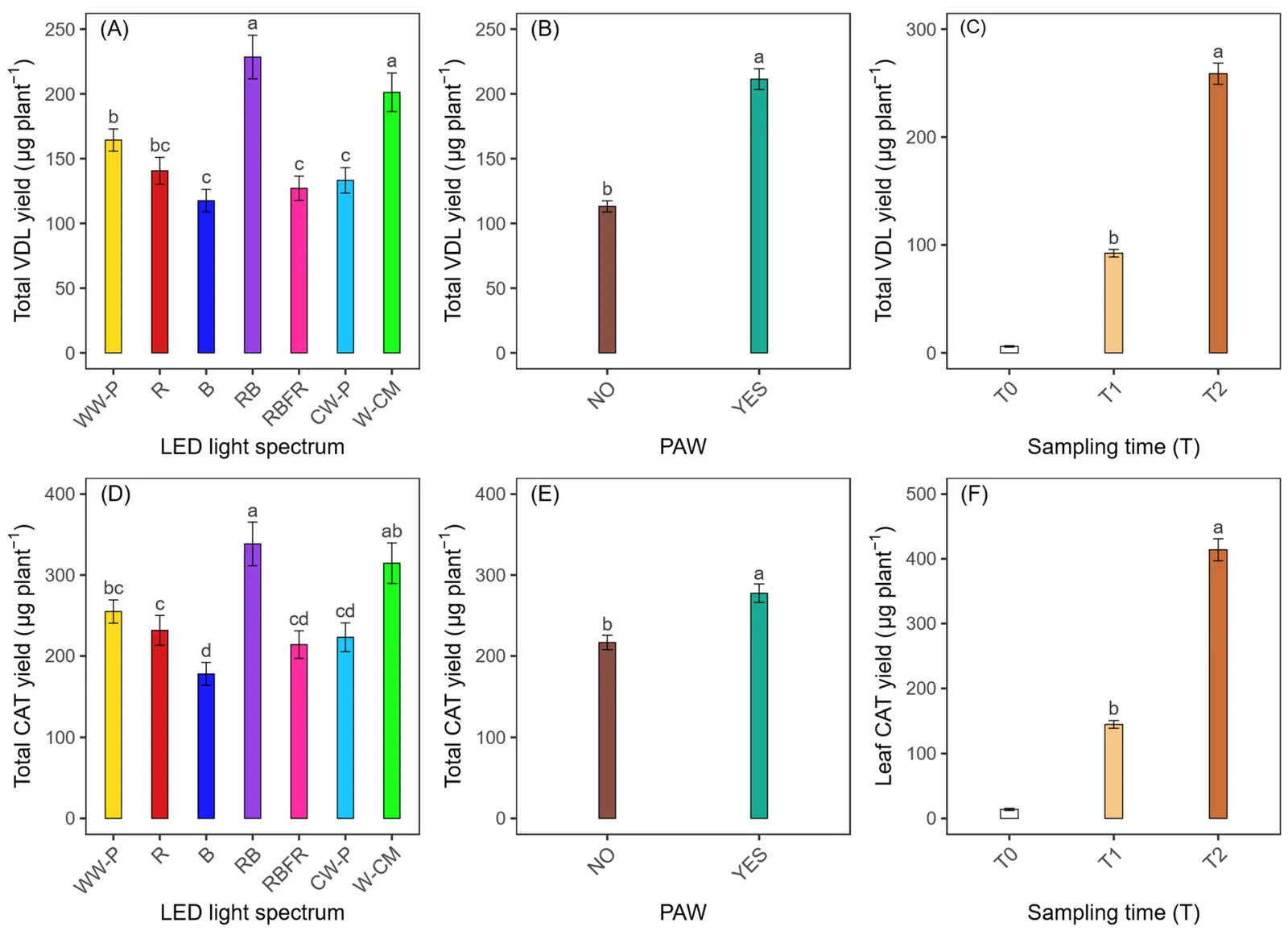
Treatment effects on total (leaf +root) vindoline (VDL) and catharanthine (CAT) yields in Madagascar periwinkle. (**A**,**D**) LED light spectrum (WW-P, R, B, RB, RBFR, CW-P, and W-CM) on VDL and CAT, respectively. (**B**,**E**) PAW (NO = absence of PAW treatment; YES = presence of PAW treatment) on VDL and CAT, respectively. (**C**,**F**) Sampling time (T: T1 = 47 DAP, T2 = 55 DAP) on VDL and CAT, respectively; T0 (1 DAP) represents the reference baseline condition prior to the application of any experimental treatment and is labelled in this form for graphical and terminological consistency within the sampling time effect structure; therefore, T0 is not part of the post-hoc comparisons. Bars represent SE (*n* = 5). Different letters indicate significant differences (*p* ≤ 0.05) based on Tukey’s method with Benjamini–Hochberg adjustment (LED light spectrum) or *t*-tests (PAW and sampling time).

Figure 10A,D show the effect of LED light spectrum on total VDL and CAT yields. Overall, both alkaloids reached their highest values under RB light (VDL: 228.0 ± 16.9 µg plant^−1^; CAT: 338.0 ± 27.0 µg plant^−1^), whereas the lowest yields were recorded under B light (VDL: 118.9 ± 8.9 µg plant^−1^; CAT: 178.0 ± 14.2 µg plant^−1^). W-CM light also led to relatively high yields, ranking second for both VDL (201.0 ± 14.5 µg plant^−1^) and CAT (315.0 ± 25.1 µg plant^−1^). In particular, RB light resulted in significantly higher total VDL and CAT yields than all the other light spectra (e.g., 1.4- and 1.3-fold higher compared to WW-P, respectively), except for W-CM, with which it was statistically comparable. Similarly, W-CM light led to significantly higher total VDL yields compared to all the other spectra, with the exception of RB. For CAT, W-CM light produced significantly higher total yields than all the other spectra, except for WW-P and RB, with which no significant differences were observed.

##### PAW Effects

PAW treatment had an overall strong positive effect also on total alkaloid yields, which were significantly higher in treated plants than in the untreated control for both VDL (Figure 10B) and CAT (Figure 10E). Total VDL and CAT yields reached 211.0 ± 8.0 and 278.0 ± 11.0 µg plant^−1^, respectively, under PAW treatment, corresponding to significant increases of 1.9- and 1.3-fold, respectively, compared with the untreated condition.

##### Sampling Time Effects

A pronounced non-linear temporal pattern was observed for both total VDL and CAT yields across sampling times (Figure 10C,F), characterized by a rapid increase at early developmental stages followed by a more gradual accumulation. Total yields were lowest at T0 (VDL: 6.0 ± 0.7 µg plant^−1^; CAT: 13.9 ± 1.7 µg plant^−1^) and reached their highest values at T2 (VDL: 258.7 ± 9.8 µg plant^−1^; CAT: 414.0 ± 17.0 µg plant^−1^). In particular, a steep increase was observed between T0 and T1 (15.4-fold for VDL and 10.4-fold for CAT), followed by a further significant increase from T1 to T2 (about 2.8-fold for VDL and 2.9-fold for CAT). Overall, total VDL and CAT yields at T2 were substantially higher than at T0, corresponding to increases of approximately 43.1-fold and 29.8-fold, respectively.

## 3. Discussion

### 3.1. LED Light Spectrum Effects

#### 3.1.1. DMs

RB light generally promoted DM accumulation in both leaves and roots compared with the other tested spectra, resulting in a significant increase in total DM. In most cases, the combined use of R and B light promotes superior crop growth and performance relative to monochromatic R or B treatments [34,35,36,37]. A large body of evidence has shown that RB light (particularly in LED-based systems) enhances the excitation of photosynthetic pigments and, across a wide range of plant species and cultivation conditions, improves overall photosynthetic performance. Reported responses include increased CO_2_ assimilation rates, enhanced electron transport efficiency, and higher chlorophyll content relative to plants grown under single-wavelength R or B light [38,39].

This enhancement is primarily attributed to the complementary absorption spectra of chlorophylls and carotenoids, which increase light-harvesting efficiency. Moreover, RB light provides a more balanced excitation of Photosystem II and Photosystem I, thereby optimizing energy distribution and promoting more efficient electron transport within the photosynthetic apparatus [38].

Beyond its role in photosynthesis, R and B light also regulate the biosynthesis and spatial distribution of key plant hormones, including auxins, cytokinins, and gibberellins, which are essential regulators of plant growth and development [40]. Auxins mainly stimulate cell elongation and control apical dominance, root initiation, and vascular differentiation [41]. Gibberellins promote cell expansion in stems and leaves by enhancing elongation and increasing internode length [42], whereas cytokinins control shoot apical meristem activity, leaf primordium formation, and shoot growth by stimulating cell division [43].

B light generally exerts a stronger effect on auxins and gibberellins, stimulating auxin biosynthesis in various tissues and contributing to phototropic responses, while potentially increasing gibberellin levels via photoreceptor-mediated signalling cross-talk [44,45,46]. In contrast, R light predominantly enhances the biosynthesis and signalling of auxins and cytokinins through phytochrome-mediated regulation of genes involved in their production and downstream signalling pathways [47].

The superiority of RB light in promoting plant growth has been consistently reported across multiple species, including Madagascar periwinkle. For example, Li et al. [48] reported that the DM of *Lycoris longituba* was significantly higher under RB light (1:2 ratio) than other spectral treatments, with additional RB ratios (1:1 and 2:1) also exceeding the performance of W and R light. Similarly, Fan et al. [37] showed that Chinese cabbage (*Brassica campestris*) grown under RB light (6:1) accumulated significantly greater DM than plants exposed to other light spectra. Naznin et al. [49] likewise reported comparable trends in coriander (*Coriandrum sativum*), with increased biomass under various R:B ratios relative to 100% R light. Moreover, Quadri et al. [24] observed that RB (6:1) tended to increase root DM compared to W, B, and R light, while Li et al. [20] showed that leaf and stem DM were significantly higher under R7B1 (an RB ratio very close to that of the present study) relative to R2B1 and W light.

Taken together, the increased DMs observed under RB light may be mechanistically attributed to the synergistic enhancement of photosynthetic pigment excitation and photoreceptor activation. These integrated processes improve photosynthetic efficiency, regulate hormone-mediated growth pathways, and ultimately promote greater biomass accumulation.

Furthermore, regarding total DM, its significant enhancement under RB treatment may be linked to both higher leaf DM and elevated alkaloid concentrations under the same spectral condition. Indeed, LED light spectrum significantly influenced both leaf and root DM, with RB generally producing the highest values for both variables.

To quantify the drivers of the significant total DM increase under RB light treatment, a logarithmic decomposition analysis based on the LMDI [50,51,52,53,54,55,56,57,58,59] was applied. This approach was used to determine which variables contributed most to the observed increases in total DM, as well as in leaf, root, and total yields of VDL and CAT under specific reference conditions.

In particular, three complementary LMDI-based approaches were employed to determine these contributions. First (*i*), a *simplified version of the multiplicative LMDI* with unit weights (equivalent to a simple logarithmic decomposition) was applied to multiplicative variables such as leaf and root VDL and CAT yields in order to quantify the contributions of alkaloid concentrations and DMs to their respective alkaloid yields. Second (*ii*), an additive *LMDI “structural” (organ-specific) approach* was applied to additive variables such as total DM and total VDL and CAT yields to separate the contributions of leaf and root DM to total DM, as well as the contributions of leaf and root yields of VDL and CAT to their respective total yields. Third (*iii*), an *additive LMDI “physiological” (single-component) approach* was applied to additive variables such as total VDL and CAT yields, which depended on both DMs and concentrations, to quantify the contribution of individual physiological variables. A detailed description of the analytical approach is provided in the Supplementary Materials.

The increase in total DM under RB light was mainly driven by leaf DM. The structural LMDI decomposition showed that 81.40% of the increase in total DM was attributable to leaf DM, whereas only 18.60% was associated with root DM (Spreadsheet S1). These results indicate that the stimulatory effect of RB light on biomass accumulation was primarily associated with enhanced leaf growth rather than increased root development.

#### 3.1.2. VDL and CAT Concentrations

Light spectrum significantly affected leaf VDL and CAT concentrations, though in most cases, no significant differences were detected among the tested spectra. Nevertheless, a consistent trend was the inhibitory effect of B light on alkaloid accumulation. In fact, B light resulted in the lowest values in both cases, significantly reducing leaf VDL content compared with WW-P light and significantly decreasing leaf CAT concentration relative to all other spectra, except W-CM, with which no significant difference was observed.

The influence of light spectrum on alkaloid biosynthesis remains only partially understood, and the specific role of B light is still unclear, with contradictory findings reported in the literature. In several medicinal species, including white spider lily (*Lycoris longituba*), happy tree (*Camptotheca acuminata*), wild coffee (*Psychotria leiocarpa*), black-jointed dendrobium (*Dendrobium officinale*), and summer snowflake (*Leucojum aestivum*), B light irradiation has been shown to stimulate alkaloid production [48,60,61,62] and, in the first three species, it has also upregulated genes involved in their biosynthetic pathways. However, in Madagascar periwinkle, B light appears to inhibit the biosynthesis of VDL and CAT in leaves. This trend was reported by Fukuyama et al. [21] and Quadri et al. [24], who observed that B light generally reduced leaf VDL and CAT levels compared with W, R, and RB treatments.

In contrast, at the root level, combined RB light overall promoted the biosynthesis of both VDL and CAT. Specifically, RB significantly increased CAT accumulation and, at the same sampling time, also enhanced VDL content. While the influence of light spectrum on alkaloid biosynthesis is still relatively underexplored, knowledge regarding its specific effects on root alkaloid production is even more limited, particularly in Madagascar periwinkle [24]. Previous studies indicate that, beyond B light, whose effects appear to be species-dependent as discussed above, R and RB light can also positively regulate alkaloid accumulation, including VDL and CAT, as well as the transcription of genes involved in their biosynthetic pathways, not only in leaves but also in roots [19,20,24,25,63]. VDL and CAT biosynthesis proceeds through three major phases within the TIA pathway: upstream, midstream, and downstream stages [64]. In the upstream stage, the precursors tryptamine and secologanin are synthesized via the shikimate and monoterpenoid pathways, respectively. Tryptophan synthase (*TS*) catalyzes the formation of tryptophan, which is subsequently decarboxylated by tryptophan decarboxylase (*TDC*) to yield tryptamine, while secologanin is produced through the terpenoid pathway via secologanin synthase (*SLS*). The midstream stage comprises the condensation of tryptamine and secologanin, catalyzed by strictosidine synthase (*STR*), producing strictosidine as the central intermediate of TIA biosynthesis. Following deglycosylation by strictosidine β-glucosidase (*SGD*), strictosidine-derived aglycones give rise to multiple branch pathways. Among these, O-acetylstemmadenine represents a key metabolic branching point leading either toward CAT or toward tabersonine. The downstream stage begins from the tabersonine branch and leads to vindoline (VDL) biosynthesis. Tabersonine is converted into VDL through seven consecutive enzymatic steps mediated by tabersonine 16-hydroxylase (*T16H1/2*), 16-hydroxytabersonine-O-methyltransferase (*16OMT*), tabersonine-3-oxygenase (*T3O*), tabersonine-3-reductase (*T3R*), N-methyltransferase (*NMT*), desacetoxyvindoline-4-hydroxylase (*D4H*), and deacetylvindoline-4-O-acetyltransferase (*DAT*) [64,65].

R light is recognized as a regulator of alkaloid biosynthesis, acting through phytochromes and secondary messengers such as G proteins and calmodulin (CaM) [66]. In *C. roseus*, R light has been demonstrated to stimulate leaf VDL biosynthesis by inducing the expression of the transcription factor GATA1 as well as key genes involved in the VDL biosynthetic pathway, including *T16H2*, *T3O*, *T3R*, *D4H*, and *DAT* [63]. Ohashi-Kaneko et al. [22] showed that, under a constant light intensity (150 μmol m^−2^ s^−1^), R light, compared to W light, significantly enhanced leaf VDL concentrations, whereas Quadri et al. [24], using the same PPFD, reported that, in contrast to the present study, R light increased CAT concentration in leaves and VDL concentration in roots relative to W, B, and RB light.

Furthermore, regarding the combined application of R and B (RB) light, Quadri et al. [24] also demonstrated that RB treatment significantly increased leaf VDL levels compared with W, B, and R light, and also enhanced root CAT concentration relative to W and R light, showing comparable results to B light. Likewise, Fukuyama et al. [21] observed that RB light at the same PPFD tended to elevate leaf VDL and CAT concentrations compared with W, R, and B light, although these differences were not statistically significant. Zhang et al. [67] reported that RB light, regardless of the specific R:B ratio tested, overall enhanced the concentrations of (R, S)-goitrin, indigo, and indirubin in the underground organs of *Isatis tinctoria* compared with W, B, and R light.

Therefore, the enhanced accumulation of VDL and CAT in the roots under RB light may be mechanistically explained by tissue-specific responses to light and differential regulation of the TIA biosynthetic pathway. In leaves, the primary site of precursor and intermediate biosynthesis, the downstream steps leading to VDL and CAT formation may be relatively stable across R-, RB-, RBFR-, and W-based spectra under the experimental conditions tested. This lack of significant spectral differences suggests that leaf TIA biosynthesis may be less responsive to spectral modulation than root accumulation, at least within the tested light intensities and exposure durations. In contrast, B light may exert a general inhibitory effect on the leaf TIA pathway, possibly by downregulating key biosynthetic genes or limiting precursor availability, thereby accounting for the lower leaf VDL and CAT levels observed under B light. Consequently, RB light does not appear to significantly enhance leaf alkaloid concentrations under the present conditions.

In roots, which do not directly perceive light, RB light might enhance alkaloid accumulation via systemic signalling or phloem-mediated translocation of intermediates from leaves. However, no active leaf-to-root long-distance transport mechanisms for VDL or CAT have been conclusively identified to date [65,68]. The few characterized transporters, such as CrTPT2 (belonging to the pleiotropic drug resistance (PDR) family of ATP-binding cassette (ABC) transporters), are involved in intracellular trafficking within leaf tissues (e.g., CAT export to the leaf wax layer) and are not reported to be present in root tissues [69]. Thus, long-distance transport of these alkaloids remains hypothetical.

The prevailing literature describes VDL and CAT biosynthesis as largely confined to aerial organs of Madagascar periwinkle. VDL is generally considered restricted to green tissues (especially leaves) [63], and transcriptomic analyses consistently report only leaf-specific expression of key genes of the VDL branch (e.g., SGD and DAT), with no expression or presence detected in roots [70]. Similarly, CAT biosynthesis is primarily associated with young leaf epidermal tissues, where the compound accumulates in vacuoles and is secreted onto the waxy surface [6,69]. In some cultivars (e.g., Dhawal), CAT has been detected in roots, though at very low levels [70,71]. Organ-resolved transcriptomic, cytological, and metabolomic studies further support a strong spatial specialization of TIA metabolism, with leaves accumulating VDL, CAT, and their dimeric derivatives (VBL and VCR), whereas roots predominantly accumulate alternative indole alkaloids (e.g., hörhammercine, ajmalicine, and serpentine) [65,70,72].

Contrary to this widely accepted spatial model, the present study consistently detected both VDL and CAT in root tissues. Notably, average root VDL concentrations exceeded those measured in leaves, and average root CAT levels were comparable to foliar values (Figure S4) as previously reported by Quadri et al. [24], independently confirming substantial root accumulation of these alkaloids. These findings challenge the widely accepted aerial confinement of the VDL branch and suggest that the spatial organization of TIA metabolism in *C. roseus* may be more flexible than previously reported.

Under this perspective, the presence of VDL and CAT in roots may reflect either (i) activation of the corresponding biosynthetic branches within root tissues under specific physiological conditions, implying potential local synthesis, or (ii) systemic redistribution processes from aerial organs that have not yet been mechanistically characterized. In the first scenario, the dominant R component of RB light might stimulate the expression of downstream TIA biosynthetic genes, promoting the conversion of intermediates into final alkaloids, while the B component, although insufficient alone, may enhance upstream precursor supply. This combined effect could increase metabolic flux through the TIA pathway in roots, explaining the pronounced accumulation of VDL and CAT under RB light. Alternatively, RB light might enhance long-distance transport, potentially stimulating the activity or expression of transport systems involved in the redistribution of TIA intermediates or end products from leaves to roots. In this case, RB light would not necessarily induce de novo synthesis in roots but could promote systemic allocation of these alkaloids from aerial tissues. Importantly, both mechanisms could operate simultaneously, so that systemic redistribution may synergize with local biosynthesis to produce the observed root accumulation of VDL and CAT under RB light. Root tissues may also differ from leaves in their regulatory constraints, potentially rendering them more responsive to spectral modulation.

It is worth noting that the results obtained in the present study differ in some respects from those reported by Quadri et al. [24]. These discrepancies may reasonably be attributed to differences in experimental conditions. First, the duration of the pre-treatment period differed between studies: in the previous experiment, plants were exposed to white light for 60 days before LED treatments, whereas in the present study the pre-treatment lasted 37 days. The length of the pre-treatment could influence the baseline physiological state of the plants, including enzyme activity levels and precursor pools within the TIA pathway, potentially affecting the magnitude and tissue-specificity of the response to subsequent spectral treatments. Second, the overall duration of the LED treatment differed, with 92 days in the previous study versus 55 days in the present experiment. This difference in cumulative light exposure could alter the integration of photoreceptor-mediated signals over time, further contributing to the observed variation in tissue responses, including RB responsiveness. Furthermore, the present study included a broader range of light spectra (seven vs. four) and the inclusion of an additional sampling time (therefore considering it as an experimental factor), allowing more detailed comparisons and the detection of interaction effects with sampling time, indicating that the response to RB and other spectra may depend on harvesting time (see the dedicated Section 3.3.2 and Section 3.4.2 below). Collectively, these factors provide a plausible explanation for the observed differences between the two studies.

#### 3.1.3. VDL and CAT Yields

RB light markedly improved leaf, root, and total VDL and CAT yields. On a general level, the notable improvements in VDL and CAT yields in both leaves and roots under RB lighting can be attributed to higher DMs as well as elevated alkaloid concentrations under the same spectral condition; similarly, the overall enhanced total VDL and CAT yields under the RB spectrum can be explained by the higher alkaloid yields observed in both leaves and roots. Indeed, ANOVA results showed that LED light spectrum significantly affected alkaloid concentrations, DMs, and alkaloid yields, with RB generally producing the highest values across all variables. However, it is worth explicitly identifying the key drivers behind these increases. At leaf level, the main contribution to the overall increased yields under RB was due to the enhanced leaf DM for both VDL and CAT. According to the multiplicative LMDI-based analysis, 69.68% (VDL) and 68.78% (CAT) of the increase in leaf yield were attributable to leaf DM, with the remaining contributions associated with changes in concentration (Spreadsheet S1). In contrast, at the root level, alkaloid concentration represented the main driver. Under RB, 66.49% (VDL) and 60.40% (CAT) of the increase in root yield were attributable to concentration. These results indicate that leaf DM represents the main physiological driver of yield variation in leaves, whereas alkaloid concentration plays the dominant role in determining root yields.

At the whole-plant level (leaves + roots), the increase in VDL and CAT yields under RB light was primarily driven by leaf alkaloid yield from a structural (organ-specific) perspective, whereas from a physiological (component-specific) perspective, leaf DM emerged as the dominant factor. Specifically, the additive structural LMDI-based analysis showed that, under RB conditions, 54.59% of the increase in total VDL yield and 70.96% of that in total CAT yield were attributed to leaf alkaloid yields, while the remaining were explained by root alkaloid yields (Spreadsheet S1). Similarly, the additive physiological LMDI-based approach identified leaf DM as the main driver of both total VDL and CAT yields, contributing to 38.72% and 48.73% of their respective increases. In contrast, secondary drivers were yield-specific: leaf alkaloid concentration contributed to 21.91% of the increase in total CAT yield, whereas root alkaloid concentration contributed to 29.13% of the increase in total VDL yield.

Together, these results emphasize that leaf growth predominantly drives whole-plant alkaloid accumulation under RB light conditions, while variations in alkaloid concentrations (particularly in roots) represent a secondary yet significant factor influencing total yields.

### 3.2. PAW Effects

#### 3.2.1. DMs

PAW application significantly increased the DM of both leaves and roots, while also enhancing total DM at the same sampling time for each PAW condition (the effect of PAW on total DM was significantly time-dependent; see Section 3.4.1 below). Previous studies have reported that plasma treatments can stimulate plant growth [73]. However, most research on PAW in agriculture has focused on direct seed treatment or early seedling development [74]. For example, Takaki et al. [75] conducted a hydroponic greenhouse experiment on cabbage (*Brassica rapa* var. *perviridis*) and observed that PAW increased plant DM by up to 6.6-fold relative to the control. Similarly, Takahata et al. [76] examined the influence of bubble discharge in water on plant growth using spinach (*Spinacia oleracea*), radish (*Raphanus sativus* var. sativus), and strawberry (*Fragaria* × *ananassa*), reporting a marked increase in the DM of the harvested edible portions.

Although the mechanisms underlying PAW-induced growth enhancement remain under investigation, current evidence suggests that multiple interacting factors are involved. First, NO_3_^−^ represents a primary source of nitrogen (N) and is readily absorbed by plants as an essential macronutrient [77]. Its presence is therefore considered a key driver of growth stimulation [75]. NO_3_^−^-N is absorbed by plant roots and subsequently reduced by nitrate reductase and nitrite reductase within plant cells. This reduction pathway ultimately produces ammonium ions, which are incorporated into amino acids and other essential biomolecules that support plant growth [30]. Although NO_2_^−^ is generally toxic to many plants, certain species can utilize this reactive nitrogen species (RNS) as a nitrogen source [78]. The increased DM and overall healthy development observed under PAW in the present study suggest that *C. roseus* may tolerate (or even benefit from) these reactive nitrogen species. In addition, the consumption of bicarbonate ions during plasma activation may further contribute to enhanced plant growth [79].

Second, reactive oxygen species (ROS), particularly H_2_O_2_, function as important signalling molecules in plants. At controlled concentrations, H_2_O_2_ can enhance stress tolerance, reduce oxidative damage, and help maintain normal physiological and metabolic processes [30,78]. İşeri et al. [80] demonstrated that low external concentrations of H_2_O_2_ increased ascorbate peroxidase (APX) activity and proline accumulation in tomato seedlings, thereby improving osmotic regulation, membrane stability, antioxidant capacity, and tolerance to cold stress. APX plays a key role in enzymatic antioxidant defence by detoxifying ROS, while malondialdehyde (MDA), a product of lipid peroxidation, is commonly used as an indicator of membrane damage [81]. Sayed and Gadallah [82] reported that H_2_O_2_ treatment reduced both H_2_O_2_ and MDA levels in pea seedlings by stimulating proline and ascorbic acid accumulation, thereby alleviating cadmium-induced growth inhibition.

Finally, RONS may promote plant growth through their effects on phytohormone balance and gene expression. RONS can modulate the balance between indole-3-acetic acid (IAA) and abscisic acid (ABA). IAA stimulates cell elongation [30], and its levels have been shown to increase significantly in mung bean sprouts following plasma treatment [83]. In contrast, ABA inhibits growth by suppressing cell division and elongation. RONS may also activate growth-related gene expression, such as Mitogen-Activated Protein Kinase 2 (MAPK2), via the MAPK signalling cascade, while simultaneously reducing ABA levels, thereby enhancing plant growth and development [84].

In summary, the significant increase in leaf DM under PAW treatment is likely the result of synergistic mechanisms. NO_3_^−^ provides a readily available N source; ROS such as H_2_O_2_ act as signalling molecules that enhance stress tolerance and antioxidant defences; and RONS, more generally, modulate hormonal balance and gene expression. These combined effects promote cell division and elongation, ultimately leading to increased biomass accumulation.

#### 3.2.2. VDL and CAT Concentrations

Overall, PAW treatment promoted VDL concentrations in both organs, significantly increased CAT concentration in leaves, but did not affect CAT concentration in roots. In micropropagated Madagascar periwinkle plants, PAW exposure has been reported by Zambon et al. [28] and Contaldo et al. [27] to enhance *SGD* gene expression, a key midstream enzyme necessary for biosynthetic flux toward both VDL and CAT pathways. The same authors suggested that this activation of *SGD* may be associated with the presence of H_2_O_2_. This interpretation is supported by Tang et al. [85], who observed that different concentrations of exogenous H_2_O_2_ increased the accumulation in the leaves of alkaloids such as VDL, CAT, and VBL. These findings indicate that H_2_O_2_ may function as a signalling molecule capable of triggering stress-related pathways that ultimately enhance alkaloid biosynthesis. Moreover, in our prior reference study [25], we also reported that PAW treatment generally increased VDL and CAT levels in the leaves of *C. roseus* plants cultivated in an environmentally controlled growth chamber.

In addition, recent evidence suggests that NO_3_^−^, the major RONS present in the PAW used in this study, may play an important role in regulating alkaloid accumulation in Madagascar periwinkle [86]. For instance, Guo et al. [87] investigated the combined effects of UV-B radiation and different NO_3_^−^ levels on plant growth and alkaloid production, reporting that the interaction between UV-B exposure and N-NO_3_^−^ supply increased the levels of VDL, CAT, and VBL. The authors suggested that enhanced N availability provides additional substrates required for UV-B-induced alkaloid biosynthesis, thereby promoting alkaloid accumulation.

Consequently, the involvement of NO_3_^−^ introduces another element that could explain the observed increase in CAT and VDL in the leaves and VDL in the roots. NO_3_^−^ functions not only as an essential nutrient but also as a signalling molecule capable of modulating plant responses to environmental *stimuli*. The findings of Guo et al. [87] further clarify this relationship by highlighting the critical role of N sources in metabolic pathways associated with alkaloid production. Similar considerations may also apply to NO_2_^−^, which could serve as an additional N source alongside NO_3_^−^.

In contrast, the absence of a PAW effect on CAT accumulation in roots may be explained by two main hypotheses. First, assuming that CAT biosynthesis is indeed active in roots and that the corresponding branch of the TIA metabolic pathway operates in root tissues, PAW treatment may not significantly influence the expression of genes involved in this specific biosynthetic branch. Second, also assuming the existence of a systemic transport mechanism of CAT from leaves to roots, PAW treatment may fail to stimulate transport systems responsible for this long-distance redistribution. Finally, both mechanisms may operate simultaneously, meaning that PAW may neither stimulate local CAT biosynthesis in roots nor enhance systemic transport from aerial tissues. It should be noted that this interpretation remains hypothetical, as no studies to date have specifically investigated the effect of PAW on alkaloid accumulation in Madagascar periwinkle roots. Indeed, for instance, in our earlier reference work [25] we evaluated the effects of PAW only on CAT and VDL levels in leaves.

From a mechanistic perspective, the overall increase in VDL concentrations in both organs and the increase in CAT concentration in leaves under PAW treatment may be attributed to the combined signalling effects of ROS and RNS present in PAW. In particular, H_2_O_2_ may act as a stress-related signalling molecule capable of activating transcription factors and key genes involved in the TIA biosynthetic pathway, including those responsible for strictosidine formation and downstream steps leading to VDL and CAT biosynthesis. This activation may enhance metabolic flux through the pathway, thereby promoting VDL and CAT accumulation. At the same time, NO_3_^−^ and NO_2_^−^ may contribute through a dual role. On one hand, they provide additional N resources required for the biosynthesis of N-containing SMs such as TIAs; on the other, they function as signalling molecules regulating nitrogen-dependent metabolic pathways. This combined nutritional and regulatory effect may increase precursor availability and enzymatic activity along the TIA pathway. Collectively, these mechanisms could enhance VDL accumulation in both organs and CAT accumulation in leaves.

Conversely, the absence of a response in root CAT concentration suggests that the regulatory steps controlling CAT accumulation in roots may be either weakly responsive to ROS/RNS signalling or constrained by organ-specific metabolic regulation, thereby limiting the effect of PAW on this metabolite in root tissues.

#### 3.2.3. VDL and CAT Yields

On a general level, PAW application promoted VDL and CAT yields. The observed increases in leaf and root VDL yields, as well as in leaf CAT yields, can be attributed to both higher DMs and elevated alkaloid concentrations. Similarly, the enhanced total VDL and CAT yields were driven by increased alkaloid yields in both leaves and roots. Indeed, ANOVA showed that PAW significantly affected DMs, alkaloid concentrations, and alkaloid yields across organs, with the YES condition significantly increasing all these variables. The only exception was root CAT yield, which increased under YES conditions only at the same sampling time, particularly at T2 (see Section 3.4.3).

However, while these results indicate that multiple components contributed to the observed increases, a more detailed analysis (LMDI-based decomposition) permitted us to identify their relative importance and determine the main drivers underlying these yield responses. At the single organ level, the increase in VDL under PAW was primarily driven by higher alkaloid concentrations in both leaves and roots. According to the multiplicative simplified LMDI-based decomposition analysis, 79.51% of the increase in leaf VDL yield and 63.34% of that in root VDL yield were attributable to concentration, with the remaining contributions due to changes in DM (Spreadsheet S1). In contrast, for CAT, biomass accumulation played the dominant role. Under PAW, 51.41% of the increase in leaf CAT yield and 75.80% of that in root CAT yield were attributable to DM, while the remaining contributions were associated with changes in alkaloid concentration.

At the whole-plant level, total VDL and CAT yield increases under PAW were primarily driven by leaf alkaloid yield from a structural (organ-specific) perspective. From a physiological (single-component) perspective, however, different dominant factors emerged for the two alkaloids. For VDL, leaf concentration represented the main driver, whereas for CAT, leaf DM was the dominant factor.

Specifically, the additive structural LMDI-based analysis showed that, under PAW, 82.04% of the increase in total VDL yield and 87.10% of the increase in total CAT yield were attributable to leaf yield, with the remainder explained by root contributions. On the other hand, the additive physiological LMDI-based analysis revealed distinct dominant drivers for the two alkaloids. The increase in total VDL yield was mainly driven by leaf alkaloid concentration (65.92%), whereas that of total CAT yield was primarily driven by leaf DM (44.16%), with leaf alkaloid concentration also making a substantial contribution (41.73%). Other factors, including root-related components, played comparatively minor roles.

Overall, these results indicate that PAW enhances total CAT yield mainly by promoting leaf DM accumulation, whereas total VDL yield is driven primarily through enhanced leaf VDL concentration.

### 3.3. Sampling Time Effects

#### 3.3.1. DMs

Leaf, root, and total DM increased dramatically across the different sampling times, indicating a progressive accumulation of biomass during plant development. This pattern reflects sustained growth dynamics and is likely influenced by environmental conditions such as nutrient availability, soil moisture, and generally favourable cultivation conditions throughout the cultivation period. The greater leaf and root DM observed at T2 suggests that plants had reached a more advanced developmental stage, potentially associated with improved resource acquisition and enhanced photosynthetic capacity. Comparable temporal increases in leaf, root, and total DM over time in *C. roseus* have also been reported by Quadri et al. [24] and by our previous reference study [25], supporting the findings observed in the present study.

With respect to total DM, its increase was predominantly driven by the contribution of leaf biomass. Specifically, the additive LMDI-based analysis revealed that the increase in total plant DM observed at T2 relative to T1 and T0 was largely attributable to leaf DM, accounting for 81.93% and 82.00% of the increase, respectively, while root DM contributed 18.07% and 18.00%. The mean contribution across all pooled comparisons (T2 vs. T1 and T0) was 81.99% for leaf DM, with the remaining 18.01% attributable to root DM. These results clearly indicate that temporal biomass accumulation was overwhelmingly driven by leaf growth, with root contribution remaining comparatively limited.

#### 3.3.2. VDL and CAT Concentrations

The temporal dynamics of VDL and CAT differed depending on the plant organ. In leaves, on a general level, the concentrations of both alkaloids followed a non-linear complex pattern, peaking at T0, sharply decreasing at T1, and subsequently significantly increasing at T2, although remaining lower than at T0. In our earlier reference study [25], we observed a significant increase in alkaloid concentrations from T1 to T2; however, no T0 sampling was conducted, preventing the assessment of baseline levels prior to treatment application. In contrast, Quadri et al. [24] reported that both compounds were drastically higher at T0 compared to T1. This observation provides a plausible explanation for the patterns observed in the present study. It appears that VDL and CAT levels peak around the 60th day of cultivation, subsequently decline, and then undergo a secondary increase. Nevertheless, even at later sampling times, as observed in this study and in Quadri et al. [24], alkaloid levels remained lower than those recorded at T0, at least within the overall cultivation time frame considered, namely until 152 days of cultivation.

In roots, by contrast, at the overall level VDL and CAT concentrations showed a marked increasing pattern from T0 to T2. For VDL, this trend was light spectrum-dependent and was observed only when comparing the same spectral conditions across time (see Section 3.4.2). Quadri et al. [24] reported a similar pattern, with concentrations of both alkaloids rising notably from T0 to T1. These findings suggest that, at the root level, VDL and CAT accumulation appear to follow a progressive increase up to the developmental stages examined of 152 days of cultivation.

#### 3.3.3. VDL and CAT Yields

VDL and CAT yields increased substantially from T0 to T2. Considering only the experimental period T1-T2 (i.e., excluding T0, which was also not included in the post-hoc analysis), the significant increases observed in both leaf and root yields from T1 to T2 were driven by concurrent increases in both DM and alkaloid concentration, as sampling time significantly affected these variables in both organs. Plants harvested at T2 exhibited significantly higher biomass and alkaloid concentrations compared to those collected at T1. Similarly, the increase in total VDL and CAT yields can be attributed to the combined enhancement of leaf and root yields, with T2 plants showing markedly higher values than those at T1.

However, a more complete and precise understanding of the temporal dynamics also requires the inclusion of T0 in the analysis. Therefore, to disentangle and quantitatively assess the relative contribution of the underlying drivers to the observed increases in leaf and root yields across the entire temporal framework, the LMDI-based decomposition analysis was applied. At the level of leaf yields, the multiplicative simplified LMDI-based approach indicated that leaf DM emerged as the dominant driver of yield increases at T2 across multiple temporal comparisons. Specifically, when comparing T2 with T1, T2 with T0, and T2 relative to both previous time points, leaf DM consistently accounted for the majority of the increase in leaf yields. In particular, considering the T2 vs. T1 and T0 framework, leaf DM contributed 94.56% to the increase in leaf VDL yield and 101.15% to that of leaf CAT yield, whereas variations in leaf alkaloid concentration accounted for only 5.44% and −1.15%, respectively (Spreadsheet S1). These findings demonstrate that the temporal enhancement of leaf yields is overwhelmingly driven by leaf biomass accumulation, regardless of the reference time point considered. At the level of root yields, an opposite pattern was observed. Across comparisons involving T2 vs. T1, T2 vs. T0, and T2 relative to both previous time points, the relative contribution of root DM and root alkaloid concentration varied depending on the temporal reference. Root DM contributed more strongly to the increase from T1 to T2, whereas root alkaloid concentration had a greater influence in comparisons involving T0. When specifically considering the overall mean pooled comparison (T2 vs. both previous time points combined), root alkaloid concentration showed a slightly higher contribution than root DM, accounting for 53.13% of the increase in VDL yield and 53.87% of that in CAT yield, compared with 46.87% and 46.13%, respectively, attributable to root DM. This suggests that, unlike leaves, root yield dynamics are more dependent on metabolic regulation of alkaloid accumulation and appear more sensitive to temporal variation.

At the level of total yields, from a structural perspective, the additive structural LMDI analysis indicated that leaf yield, rather than root yield, was the primary driver of the increase in total VDL and CAT yields at T2 across all temporal comparisons (T2 vs. T1, T2 vs. T0, and T2 vs. both previous time points). Specifically, considering the overall mean pooled comparison T2 vs. T1 and T0, leaf yield contributed 73.01% of the increase in total VDL yield and 81.58% of that in total CAT yield, whereas root yield accounted for 26.99% and 18.42%, respectively.

From a physiological perspective, the additive LMDI-based approach further highlighted the dominant role of leaf DM in driving the observed increases in total yields across all relevant comparisons. In particular, considering the overall mean pooled comparison of T2 vs. T1 and T0, leaf DM accounted for 70.82% of the increase in total VDL yield and 82.90% of that in total CAT yield. All other components showed substantially lower contributions, with root alkaloid concentration representing the second most relevant factor, albeit with a modest effect overall, followed by root DM and leaf alkaloid concentration, which contributed marginally to the observed changes.

Taken together, these considerations demonstrate that, at the whole-plant level, leaves, and particularly leaf DM, are the primary drivers of the observed increases in total VDL and CAT yields. Therefore, strategies aimed at maximizing total alkaloid production, regardless of the specific treatment applied, should primarily focus on enhancing leaf growth and biomass accumulation.

### 3.4. Combined (Interaction) Effects

#### 3.4.1. Total DM

PAW treatment effects on total DM were dependent on sampling time, with the YES:T2 combination showing significantly higher values among all PAW:sampling time conditions as well as a dramatic increase relative to the baseline NO:T0. The elevated total DM under YES:T2 was mainly driven by the contribution of leaf DM. Indeed, the additive LMDI-based analysis comparing YES:T2 against the pooled set of all other conditions, including the NO:T0 baseline (YES:T2 vs. NO:T2, NO:T1, YES:T1, and NO:T0 comparison, Spreadsheet S1), indicated that this increase was largely attributable to leaf DM (81.57%), whereas root DM contributed to a lesser extent (18.43%). Comparable results were also observed when analyzing the same comparison but without considering the baseline. This indicates that the combined effect of PAW treatment and advanced developmental stage promotes total biomass accumulation through a consistent preferential allocation toward leaf growth.

Moreover, total DM generally increased under PAW treatment at the same sampling time condition and was predominantly driven by leaf DM. The additive structural LMDI analysis indicated that the significant increase in total DM under the YES:T2 vs. NO:T2 comparison was predominantly driven by leaf DM (79.53%), with a similar contribution observed for the YES:T1 vs. NO:T1 comparison (80.85%). Overall, these findings indicate that PAW effects on total biomass are consistently leaf-driven at equal sampling times, while their magnitude increases over time, likely reflecting a cumulative exposure effect that progressively enhances leaf DM and strengthens its dominant structural contribution across developmental stages.

Temporal dynamics showed that the dramatic increase in total DM at T2 compared to both T1 and T0 was predominantly leaf-driven. Indeed, structural LMDI analysis revealed that the higher total DM observed under the YES:T2 condition, relative to the pooled set of the PAW:sampling time combinations NO:T1, YES:T1, and the baseline condition NO:T0 (YES:T2 vs. NO:T1, YES:T1, and NO:T0 comparison), was largely attributable to leaf DM (81.64%); similar results were obtained when excluding the NO:T0 baseline from the comparison set. A comparable pattern was observed under the NO:T2 condition, where the increase in total DM relative to the same pooled set was also primarily driven by leaf biomass (82.22%), with consistent results when NO:T0 was excluded from the comparison. These observations indicate that PAW effects on total DM are consistently governed by a stable leaf-driven structural allocation pattern, which is progressively amplified over time, resulting in cumulative increases under prolonged exposure conditions.

#### 3.4.2. VDL Concentrations

The effect of PAW treatment varied with sampling time, highlighting a clear non-linear time-dependent response in leaf VDL accumulation. This pattern largely mirrored the trend observed for the main effect of sampling time, characterized by an initial decline from T0 to T1 followed by a recovery phase at T2, while also highlighting a consistent modulatory role of PAW across the treatment period. In particular, leaf VDL concentration peaked under the YES:T2 combination compared to all other treatment combinations, including the pre-treatment baseline (NO:T0) and, across the treatment period (T1–T2), it was consistently higher in PAW-treated plants (YES:T2 and YES:T1) than their untreated counterparts (NO:T2 and NO:T1). Overall, these findings point to a dynamic response in which the initial decrease in VDL concentration is primarily driven by time-dependent metabolic adjustment rather than by PAW application, whereas the subsequent recovery phase is further enhanced under PAW treatment, particularly at later developmental stages. Moreover, the consistently higher VDL levels observed under PAW during the treatment period indicate that its effect is maintained across sampling times and is consistent with a stimulatory influence on VDL accumulation. Importantly, the fact that VDL concentration under YES:T2 slightly exceeded the pre-treatment baseline (NO:T0) suggests that prolonged PAW exposure not only restores but also amplifies VDL biosynthesis beyond initial physiological levels.

These findings are consistent with our previous reference study [25], in which a significant interaction between PAW treatment and sampling time was reported for both leaf VDL and CAT concentrations. Although sampling times were not identical between the two studies, comparable temporal patterns emerged: VDL concentration increased from T1 to T2, with both PAW-treated and untreated plants sampled at T2 (YES:2 and NO:2) showing significantly higher levels than those collected at T1 (YES:1 and NO:1). In addition, PAW treatment enhanced VDL accumulation at T1, as YES:1 showed significantly higher concentrations than NO:1. At T2, the YES:2 combination resulted in VDL levels comparable to those observed under NO:2. More broadly, PAW-treated plants (YES:1 and YES:2) exhibited a higher VDL concentration than the corresponding untreated conditions.

Despite some differences in the magnitude of the responses, the overall agreement between the two studies supports the presence of a robust interaction between treatment and time in regulating alkaloid accumulation. Such discrepancies are likely attributed to differences in sampling timing, which can strongly influence specialized metabolism dynamics.

Notably, the previous study did not include a pre-treatment baseline (T0). Based on the results of the present study, it is plausible that, had such a baseline been included, VDL concentrations at T0 might have been comparable to those observed at later stages under PAW treatment (e.g., YES:T2), further supporting the role of PAW in sustaining or enhancing alkaloid accumulation over time.

Regarding root VDL concentration, the influence of light spectrum was strongly dependent on the harvest period. The significant increase observed under the RB:T2 combination, together with the consistently higher levels measured under RB at the same sampling time, suggests that RB light stimulates the VDL biosynthetic pathway in a time-dependent manner. In particular, the pronounced effect at T2 is likely the result of cumulative exposure to RB light over time, leading to sustained activation of VDL biosynthesis and progressive alkaloid accumulation in roots. A similar interpretation may also apply to W-CM light, particularly at T2, where W-CM:T2 exhibited VDL levels comparable to those observed under RB:T2; likewise, W-CM showed a similar performance to RB at the same sampling period. This suggests that prolonged exposure to this spectral condition may similarly support VDL biosynthesis in roots.

Moreover, W-CM light exhibited trends comparable to RB in root CAT concentration, with the highest values observed under RB and the second-highest under W-CM. This performance is noteworthy, as W-CM was the only “white” spectrum to consistently approach RB, particularly at the root level, where CW-P and WW-P generally performed worse. In contrast, W-CM maintained both VDL and CAT concentrations at levels similar to those achieved under RB, indicating a distinct spectral behaviour among the white-light treatments. This pattern may be related to its spectral composition, characterized by a relatively high R-to-B ratio (~2.8), a lower proportion of G light (19% compared to 35% in WW-P and 47% in CW-P), and a reduced FR fraction (15% vs. 2% in WW-P and CW-P). Overall, these findings tentatively suggest that higher proportions of G light, especially when combined with R and B wavelengths, might be associated with reduced VDL and CAT biosynthesis at the root level, although further investigation is required.

#### 3.4.3. Root CAT Yield

Root CAT yield was jointly influenced by PAW treatment and sampling time, with the strongest response observed under YES:T2 and mainly driven by root DM. Indeed, when comparing YES:T2 with all the other combinations, including NO:T0 (YES:T2 vs. NO:T2, NO:T1, YES:T1, and NO:T0 comparison, Spreadsheet S1), multiplicative LMDI analysis showed that root DM accounted for 60.67% of the increase, whereas root concentration contributed 39.33%. Analogue results were obtained when the same comparison was performed without including the baseline. These findings indicate that the combined PAW and T2 effect enhances root CAT yield mainly through biomass accumulation rather than changes in alkaloid concentration, indicating a shift in yield determination towards DM-driven structural components under prolonged exposure.

Furthermore, root CAT yield generally increased under PAW treatment (confirming the trend observed for the effect of sampling time alone) and was primarily driven by root biomass. The multiplicative LMDI decomposition indicated that the significant increase in root CAT yield under the YES:T2 compared to NO:T2 was predominantly driven by root DM (69.69%), with the remaining attributable to root concentration; similarly, the significant increase observed under YES:T1 vs. NO:T1 was largely driven by root DM (154.03%), while root concentration contributed negatively (−54.03%). This suggests that PAW mainly acts by promoting a strong biomass-driven amplification of CAT yield, while in early stages (T1) metabolic concentration may even be temporarily diluted or reallocated due to rapid growth or metabolic adjustment processes induced by PAW exposure.

Consistently with the trend observed for the effect of sampling time alone, root CAT yield increased dramatically from T0 to T2 across all PAW:sampling time combinations, including the NO:T0 baseline. Indeed, the enhanced root CAT yield observed under the YES:T2 relative to YES:T1, NO:T1, and NO:T0 (YES:T2 vs. NO:T1, YES:T1, and NO:T0 comparison) was largely attributable to root DM (60.82%); comparable results were also observed when analyzing the same comparison but without considering the NO:T0 baseline. A similar pattern was observed under NO:T2, where the increase compared to YES:T1, NO:T1, and NO:0 (NO:T2 vs. NO:T1, YES:T1, and NO:T0 comparison, Spreadsheet S1) was primarily driven by root DM (59.31%). However, when the same comparison was analysed without the NO:T0 baseline, root concentration (51.17%) emerged as the main contributor, although very close to root DM (48.83%).

Overall, this pattern indicates that root CAT yield is mainly driven by root DM when the baseline is included in the comparison, whereas root alkaloid concentration becomes slightly more important when the baseline is excluded (i.e., when considering only the treatment period), with both components contributing in a comparable range.

### 3.5. Scientific Implications and Novelty of the Integrated LED–PAW Approach

The present study extends our previous work [25], which provided the initial proof of concept that PAW can enhance foliar VDL and CAT concentrations under selected artificial-light conditions. In our previous study, PAW was evaluated under W fluorescent and R LED light, and the response was assessed exclusively through alkaloid concentrations in leaves. The present study moves beyond this concentration-based, leaf-focused framework by investigating how LED spectrum and PAW affect biomass accumulation and alkaloid productivity according to plant organ and harvesting time (sampling time, reflecting different developmental stages), and by integrating these dimensions at the whole-plant level. Thus, the main advance is not simply the inclusion of additional light spectra or a modified PAW application and generation procedure, but the expansion of the biological question from whether PAW can modify foliar alkaloid concentration to how spectral conditions and PAW influence alkaloid production across organs and harvesting times.

A key finding of the present study is that changes in alkaloid concentration do not necessarily translate into proportional changes in alkaloid yield, because yield reflects both metabolite concentration and biomass accumulation. By integrating these two components, the present study shows that alkaloid accumulation and productivity (yield) cannot be considered interchangeable measures and that their relationship may differ according to the plant organ, metabolite, and experimental condition. This distinction represents an important extension of our previous LED–PAW study [25], in which we considered only foliar alkaloid concentrations, and demonstrates that concentration alone is insufficient to characterize the production response of medicinal plants to environmental treatments. Accordingly, the present study provides a production-oriented framework in which changes in biomass and metabolite accumulation can be evaluated jointly rather than considered separately.

The organ-resolved analysis further extends the scope of our previous work [25] by including roots in the evaluation of VDL and CAT production. Whereas our previous LED–PAW study focused exclusively on leaves, the present study quantifies alkaloid concentrations and yields separately in leaves and roots and evaluates their respective contributions to whole-plant production. Quadri et al. [24] already provided evidence for the presence of VDL and CAT in roots; therefore, the novelty of the present findings does not lie in their detection per se, but in demonstrating that root accumulation is quantitatively relevant, varies according to spectral condition and harvesting time, and contributes measurably to whole-plant alkaloid yield. The organ-specific analysis therefore provides a broader view of alkaloid production than can be obtained from foliar measurements alone. Although the present experiment cannot distinguish between local biosynthesis and systemic redistribution, the observed differences between leaves and roots provide new quantitative evidence that the response of TIA accumulation to environmental treatments is organ-specific.

The temporal dimension was also expanded relative to our previous study [25]. Whereas our previous experiment included two post-treatment sampling points, the present study includes a pre-treatment baseline and two subsequent sampling times, allowing biomass accumulation, alkaloid concentrations, and alkaloid yields to be evaluated within a common baseline-to-later-harvesting framework. The results showed a common overall pattern of markedly increasing alkaloid productivity over the experimental period, while concentration trajectories differed according to organ and metabolite. Moreover, significant treatment:harvesting time interactions were detected for specific responses, including root CAT yield under PAW and root VDL concentration under different LED spectra. These findings indicate that the magnitude of specific treatment responses varied with harvesting time and, therefore, that treatment evaluation cannot be fully separated from the timing of harvest when the objective is to maximize alkaloid production. This temporal perspective was not available in our previous LED–PAW study [25] and represents a further dimension of the present contribution.

The LMDI-based decomposition analysis then provided a quantitative framework for explaining the basis of the yield responses identified above. Rather than representing an independent measure of treatment efficacy, LMDI was used to separate the relative contributions of biomass accumulation and alkaloid concentration to changes in VDL and CAT yields and, at the whole-plant level, to distinguish the contributions of leaves and roots. This analysis revealed that the relative importance of biomass and concentration was not uniform across treatments, organs, metabolites, or harvesting times. For example, leaf biomass accounted for a major proportion of the increase in whole-plant VDL and CAT yield under RB light and of the increase in whole-plant yield from the baseline to the later harvesting time, whereas alkaloid concentration made a larger contribution to root VDL and CAT yield under RB light and to PAW-induced increases in VDL production in both leaf and root tissues. Thus, LMDI adds a quantitative interpretation at the level of yield components by showing not only that productivity changed, but also how biomass accumulation and alkaloid concentration contributed to those changes. This quantitative decomposition could not be obtained from the concentration-based assessment used in our previous LED–PAW study [25].

The present study also introduces a new and scaled-up PAW production and application strategy [25]. In our previous study [25], PAW was generated from SDW using a corona-discharge plasma system and applied by briefly immersing individual potted plants upside down into PAW-filled jars. In the present study, PAW was generated from tap water using a hybrid corona-discharge/DBD plasma system, with a production capacity of up to 6 L per cycle, and applied by foliar spraying using a battery-powered backpack atomizer. Compared with the previous approach, which provided up to 0.5 L of PAW per cycle and required the manual handling of individual potted plants, the present procedure increased the maximum PAW volume per production cycle twelve-fold and facilitated the treatment of larger plant batches. These adaptations represent a practical and methodological extension of the more laboratory-oriented procedure used in our previous study.

Taken together, the present study therefore represents a conceptual and experimental extension of our previous LED–PAW proof-of-concept study [25]. Whereas our earlier work demonstrated that PAW can modulate foliar VDL and CAT concentrations under selected light conditions, the present study extends the assessment to alkaloid productivity by integrating a broader range of LED spectral regimes, a different PAW production and foliar application strategy, as well as biomass accumulation, metabolite concentration, organ specificity, and harvesting-time dynamics within a single multidimensional experimental framework. The combination of these dimensions provides a more comprehensive basis for interpreting LED–PAW effects on alkaloid production and reveals that the relative contribution of biomass and alkaloid accumulation varies across organs, metabolites, and harvesting times. Overall, the identification of distinct, organ- and time-dependent contributions to VDL and CAT productivity provides new biological and quantitative insight into how different LED spectral conditions and PAW treatments influence alkaloid production in CPPS, while extending the previous concentration-based proof of concept through a broader spectral evaluation and a new mode of PAW generation and application.

## 4. Materials and Methods

### 4.1. Plant Material and Growth Conditions

#### 4.1.1. Plant Material and Transplantation

Seedlings of Madagascar periwinkle “Titan Really Red,” approximately 50 days old with 2–3 pairs of leaves, were sourced from a large commercial nursery. These seedlings were provided in specialized cell trays and were promptly transplanted into 1.4 L square plastic pots (10 × 10 cm base, 17 cm height; Bamaplast, Massa e Cozzile, Italy) filled with a professional substrate (VIV COCCO TIIRENO 2, Vigorplant, Lodi, Italy).

#### 4.1.2. Growth Conditions and Pre-Treatment Acclimation Period

Following transplant, the young plants were placed in growth rooms with controlled environmental conditions, where they remained for 37 days to undergo an acclimation phase. This pre-treatment period aimed to facilitate adaptation to the new environment and allow recovery from transplant stress before the experimental phase. During this time, seedlings were maintained under a 16-h photoperiod, with a photosynthetic photon flux density (PPFD) of 100 μmol m^−2^ s^−1^, supplied by phosphor-converted warm white LED lamps (Extended white combo 150 W, 3221 K, C-LED, Imola, Italy). Light intensity was measured at the canopy level using an LP471/PAR quantum radiometric sensor (Delta Ohm S.r.l., Padua, Italy). Temperature was set at a constant 23 °C.

The duration of this pre-treatment phase was established based on previous research by Ohashi-Kaneko et al. [22,23] and Fukuyama et al. [3,21], who applied a W light acclimatization period of 32–35 days immediately after germination in studies on Madagascar periwinkle alkaloids. Given that our plants, despite being older (about 50 days), were still relatively small, a 37-day pre-treatment was chosen to ensure sufficient biomass development before initiating treatments with LED light and PAW.

Throughout the whole test, the plants were irrigated with tap water adjusted to pH 6 using nitric acid (38%) when necessary. At the end of the pre-treatment phase, healthy and uniform seedlings were selected and assigned to the respective experimental groups, as detailed in the following sections.

### 4.2. PAW Generation and Characterization

#### 4.2.1. Plasma System Setup for PAW Generation

**Figure 11:**
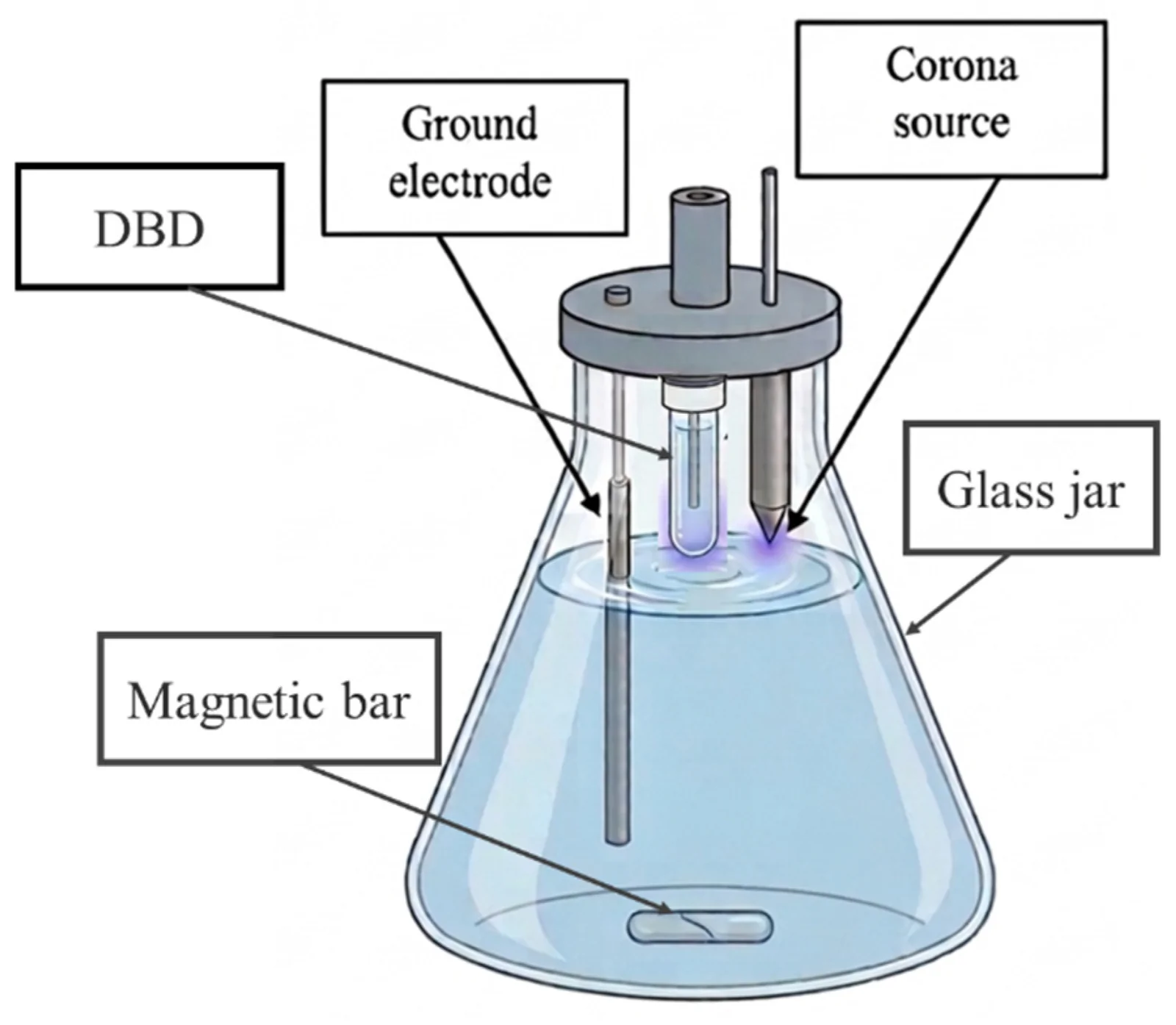
Hybrid cold atmospheric plasma (CAP) reactor configuration: Schematic representation of the hybrid CAP reactor, integrating both the dielectric barrier discharge (DBD) plasma source and the corona discharge plasma source within a single experimental setup.

A hybrid cold atmospheric plasma (CAP) system was used for PAW production (Figure 11). The main components of the system are a DBD plasma source and a Corona discharge plasma source, both accommodated in a glass jar containing 6 L of water taken from the public water supply. The chemical and physical characteristics of the water used for PAW production are reported in the Supplementary Materials, with a link to the official water-quality data provided by the public water supplier. The DBD source comprised a high-voltage electrode, consisting of a conductive aqueous solution in a glass test tube, and a ground electrode submerged in liquid. The distance between the tip of the plasma source and the liquid was fixed at 5 mm. The ground electrode consisted of a 2 mm diameter stainless steel cylinder. The corona plasma source was composed of a pointed 10 mm diameter tungsten cylinder high-voltage electrode and the same ground electrode used for the DBD source. The high-voltage electrode was positioned 5 mm from the water surface. A high-voltage microsecond-pulsed generator (Almapulse, Almaplasma, Bologna, Italy) was used to generate the plasma discharge using two different sources. A fixed frequency of 4 kHz was used for both plasma discharges.

To ensure the proper mixing of treated water, the water was continuously stirred at 1500 rpm during treatment using a magnetic stirrer.

#### 4.2.2. Operational Protocol for PAW Production

The plasma treatment was conducted by alternately activating the DBD and corona discharge sources. Each cycle consisted of 45 s of DBD exposure followed by 300 s of corona treatment. This sequence was repeated six times, resulting in cumulative treatment durations of 270 s for DBD and 1800 s for corona discharge. After completion of the plasma exposure, samples were further mixed for 3 min under continuous stirring using a magnetic stirrer to promote homogenization of RONS within the treated solution.

#### 4.2.3. Electrical Diagnostics of Plasma Discharge

Electrical characterization of the discharge was performed by monitoring voltage (V) and current (I) waveforms. Voltage measurements were acquired using a high-voltage probe (TEKTRONIX P6015A, Beaverton, OR, USA) connected to the supply line, while current was measured with a probe (TEKTRONIX TCP0030, Beaverton, OR, USA) placed on the ground connection. Signals were recorded via a digital oscilloscope (TEKTRONIX MSO46, Beaverton, OR, USA). The average power dissipated during plasma operation was determined over one voltage cycle according to the following:

$$P=\frac{1}{T}\int_{0}^{T}V(t)I(t)\mathrm{dt},$$

where (T) denotes the period of the applied voltage. The calculation of average power over one voltage cycle is based on the time-resolved voltage and current waveforms obtained from the oscilloscope. Each measurement has been repeated at least 3 times.

#### 4.2.4. Determination of pH in PAW

Aliquots of 25 mL were collected following plasma treatment for pH analysis. Measurements were conducted both prior to and after treatment using a calibrated pH meter (inoLab^®^ pH 7110, Xylem Analytics, Weilheim, Germany). Untreated water samples were analyzed under identical conditions to provide a reference baseline.

#### 4.2.5. Quantification of RONS in PAW

Quantitative determination of H_2_O_2_, NO_2_^−^, and NO_3_^−^ contents was carried out using the Amplex Red Hydrogen Peroxide Assay Kit (Thermo Fisher Scientific, Waltham, MA, USA) and the Nitrite/Nitrate Colorimetric Assay Kit (Roche, Basel, Switzerland), respectively.

Samples were dispensed into 96-well microplates (Jet Biofil, Guangzhou, China) and analyzed with a microplate reader (RT-2100C, Rayto, Shenzhen, China), which irradiates the wells with monochromatic light at a selected wavelength (λ). As a result of absorption by the chromogenic reaction products, the transmitted light intensity (I) decreased relative to the incident intensity (I_0_).

The content of the species (n) was calculated according to the Lambert–Beer law, expressed as follows:

$$n=-\frac{1}{L\sigma }\ln(\frac{I\left(\lambda \right)}{I_{0}\left(\lambda \right)})$$

where L represents the optical path length, σ the absorption cross-section, and λ the wavelength corresponding to maximum absorbance. The absorbance (A) measured by the spectrophotometer is defined as follows:

A=−ln(T)

where T denotes the transmittance. By introducing the molar extinction coefficient (ε), defined as the reciprocal of the absorption cross-section, the Lambert–Beer law can be rewritten in its conventional linear form:

A=ε L c

where c is the species content. Both reaction products exhibit maximum absorbance at 570 nm.

### 4.3. Experimental Treatments

#### 4.3.1. Definition of Treatments

Seven LED light spectra were combined with two PAW treatment conditions, resulting in a total of fourteen applied treatments. All LED modules were 150 W fixtures (C-LED, Imola, Italy) emitting specific spectra: phosphor-converted warm white (WW-P), red (R), blue (B), red–blue (RB), red–blue–far-red (RBFR), phosphor-converted cool white (CW-P), and color-mixed white (W-CM) light. The LED modules used were the Red Toplight, Blue Toplight, Hortis Toplight, Bloom Toplight, Extended White Combo, Cool White Combo, and Natural Toplight models, corresponding to the R, B, RB, RBFR, WW-P, CW-P, and W-CM spectra, respectively. The spectral composition of each treatment is expressed as the percentage contribution of the spectral components (R, B, G, and FR), or, for monochromatic lights, as peak wavelengths (Table 2). The two PAW conditions included its absence (NO) and presence (YES). To assess temporal variations in concentrations and yields of VDL and CAT (as well as in DMs), treatments were assessed at two distinct experimental sampling times (T: T1 and T2; details on the samplings and their methodology are provided in Section 4.4), resulting in 28 unique treatment combination codes (TCCs). A detailed summary of the applied treatments, including spectral composition, PAW condition, sampling times, and TCCs, is provided in Table 2. Each TCC indicates the light spectrum, PAW condition, and sampling time (T). For example, WW-P:NO:T1 refers to plants treated with sole WW-P and sampled at T1, whereas WW-P:NO:T2 refers to plants treated with sole WW-P and sampled at T2. Similarly, WW-P:YES:T1 and WW-P:YES:T2 denote plants treated with both WW-P and PAW and sampled at T1 and T2, respectively. The same coding scheme was applied to all LED:PAW treatment combinations.

The overall control treatment (irrespective of sampling time) is represented by WW-P light without PAW (WW-P:NO, control, CK); however, its definition should be interpreted within the context of the factorial experimental design adopted in this study (see Section 4.6). This design includes multiple factors (LED light spectrum, PAW, and sampling time) and their interactions, involving both multiple and simple pairwise comparisons. Accordingly, the definition of control depends on whether the analysis considers main effects or interactions (noting that some interactions, such as the three-way interaction, were not statistically significant). For single effects, controls correspond to the baseline levels of each factor considered independently [WW-P (LED light spectrum), NO (PAW), and T1 (sampling time)], whereas for interactions, controls are defined as specific combinations of factor levels used as reference points to interpret combined effects (e.g., WW-P:NO:T1 for the three-way interaction; WW-P:T1, NO:T1, and WW-P:NO for the relevant two-way interactions). A more detailed description and formal definition of all control levels across main effects and interactions is provided in the Supplementary Materials (Table S2).

#### 4.3.2. Environmental Settings and Treatment Application Protocol

Treatments were applied within the same growth rooms used for the pre-treatment period. The PPFD, photoperiod, temperature, and relative humidity of the chamber were set as follows: 150 μmol m^−2^ s^−1^, 16/8 h, 23 °C, and 70%, respectively. Canopy-level PPFD was measured with the quantum radiometric sensor used in the pre-treatment acclimatization period (LP471/PAR, Delta Ohm, Padua, Italy). The selection of a PPFD of 150 μmol m^−2^ s^−1^ was based on Fukuyama et al. [3], who identified this intensity as optimal for maximizing VDL and CAT concentrations.

While LED light treatments were applied over the course of the whole experiment (Figure S6), the treatment with PAW was performed three times using a battery-powered backpack mist sprayer (Figure S7): at the same time as the application of LED light treatments (1 DAP—day after the end of pre-treatment, 37 days after transplant, 38 total cultivation days), 41 DAP (77 days after transplant, 78 total cultivation days), and 49 DAP (85 days after transplant, 86 total cultivation days). Days after transplant were calculated excluding the day of transplant (day 0), whereas total cultivation days were calculated including the day of transplant (day 1), resulting in a +1 day offset relative to days after transplant.

The plants assigned to the PAW treatment were sprayed for 20 min with 10 L of PAW, while those assigned to the light-only treatment were sprayed for 20 min with 10 L of tap water from which the PAW was produced. PAW was generated in multiple production cycles as needed to obtain the 10 L required for each application. For each of the three applications, PAW treatment was carried out within one hour of production.

### 4.4. Samplings for Biomass and Alkaloid Temporal Analysis

#### 4.4.1. Temporal Sampling Scheme

To evaluate the temporal responses of plants for VDL and CAT production (both concentration and yield), sampling (harvest) was carried out at three distinct time points (T): (1) T0, at 1 DAP (37 days after transplant and 38 total cultivation days), immediately before treatment application, serving as the common biological baseline reference; (2) T1, at 47 DAP (83 days after transplant and 84 total cultivation days); (3) T2, at 55 DAP (final harvest; 91 days after transplant and 92 total cultivation days). At each time point, five whole plants were collected for each experimental unit. The leaves and roots were separated, dried, and weighed to determine DM, followed by biochemical analyses to quantify VDL and CAT concentrations (µg g^−1^ DM).

#### 4.4.2. Rationale for Sampling Time Points

The selection of sampling time points was based on multiple considerations, including previous research findings and the specific objectives of this study. Ohashi-Kaneko et al. [22,23] examined leaf VDL and CAT variations at 56 and 70 [23] and at 63 and 77 [22] days post-germination. In our previous reference study [25], after a 61-day pre-treatment acclimatization period common to all plants, we evaluated the temporal dynamics of VDL and CAT in Madagascar periwinkle leaves by sampling at 45 DAP (corresponding to 45 days of treatment and 106 days of total cultivation) and 70 DAP (70 days of treatment and 131 days of total cultivation). Similarly, Quadri et al. [24], following a 60-day pre-treatment acclimatization phase, assessed VDL and CAT concentrations in both Madagascar periwinkle leaves and roots at two time points: before treatment application (T0) and at a single post-treatment time point corresponding to the final harvest (T1), performed after 92 days of treatment (92 DAP = 152 days of total cultivation). These findings underscored the importance of including a true biological baseline (T0) for the temporal interpretation of alkaloid dynamics.

Therefore, to refine temporal resolution, we selected intermediate time points, particularly between T1 and T2, using shorter intervals than those adopted in previous studies. This approach was designed to better capture potential temporal changes in alkaloid accumulation following treatment application, including early responses and subsequent trends over time. It also allowed consideration of possible organ-specific patterns while ensuring that plants had fully recovered from transplant-related stress before sampling.

### 4.5. Biochemical Analyses of VDL and CAT

#### 4.5.1. HPLC-DAD-MS/MS Analysis

Determination of VDL and CAT was performed by exploiting a method based on liquid chromatography coupled with diode array detection and tandem mass spectrometry (HPLC-DAD-MS/MS) developed in our previous study [25]. Assays were carried out using a Waters Alliance e2695 chromatographic system (Waters Corporation, Milford, MA, USA) equipped with an autosampler. This system was paired with a Waters 2998 photodiode array detector (Waters Corporation, Milford, MA, USA) and a Waters Micromass Quattro Micro triple-quadrupole mass spectrometer (Waters Corporation, Milford, MA, USA) coupled to an electrospray ionization source working in positive mode (ESI+). The analysis was managed by means of Waters MassLynx 4.1 software (Waters Corporation, Milford, MA, USA). Chromatographic separation was achieved using a Waters XTerra MS C18 analytical column (100 × 2.1 mm, 3.5 µm particle size) (Waters Corporation, Wexford, Ireland) kept at room temperature and protected by a Waters VanGuard XTerra C18 guard column (Waters Corporation, Wexford, Ireland).

The mobile phase consisted of an automatic composition gradient of 0.15% formic acid in water (component A) and 0.15% formic acid in acetonitrile (component B), with a flow rate maintained at 0.3 mL/min. The gradient program began with a ratio of A:B at 90:10, held for 1 min, followed by a ramp to 70:30 within 1 min, maintained for 3 min. Then, the gradient moved to 50:50 over 1 min, held for 3 min, and then returned to initial conditions in 1 min with an additional 1-min re-equilibration phase. Both mobile phase components were filtered through Sartorius membrane filters (47 mm diameter, 0.2 µm pore size, polyamide) (Sartorius, Göttingen, Germany) and degassed using an ultrasonic bath.

For quantitative analysis of VDL and CAT, the DAD detector was set to scan within a range of 220–400 nm, while mass spectrometry employed multiple reaction monitoring (MRM) in ESI+ mode. Optimal MS parameters were determined by direct infusion of 1 µg/mL methanolic standard solutions of the target analytes at a flow rate of 20 μL/min. The optimized conditions included the following: ion source voltage at 3.5 kV, ion source temperature at 125 °C, desolvation temperature at 380 °C, and desolvation gas flow at 600 L/h. The extractor and collision exit potentials were set to 3.2 V and 1 V, respectively. Nitrogen was utilized as the desolvation gas, and argon served as the collision gas. Precursor and product ion parameters, including dwell time, cone voltage, and collision energy, were tailored for each analyte as follows. The selected parent ion for VDL was *m*/*z* 457.5; the quantifier product ion was *m*/*z* 427.5 (cone voltage: 35 V; collision energy: 40 eV), while for CAT, the parent ion was *m*/*z* 337.5 and the quantifier product ion was *m*/*z* 173.4 (cone voltage: 25 V; collision energy: 30 eV). Vindoline-d3 (VDL-d3) was used as an internal standard (IS) and monitored with the following parameters: parent ion, *m*/*z* 460.5; quantifier product ion, *m*/*z* 430.4; cone voltage, 35 V; collision energy, 40 eV.

#### 4.5.2. Sample Preparation and MEPS Extraction Procedure

Plant material (leaves and roots) was dried at 40 °C until a constant weight was reached, ground into fine powder using an electric analytical mill, and prepared for extraction. A 0.25 g aliquot of the powder was treated with 2.5 mL of 70% ethanol under constant stirring for 24 h. This process was repeated three times, after which the combined extracts were filtered, and the solvent was evaporated using a Savant SpeedVac SPD210 vacuum concentrator (Thermo Scientific, Waltham, MA, USA) at 40 °C, yielding crude extracts. The crude material was dissolved in methanol to prepare working solutions at a concentration of 1 mg/mL. After centrifugation, the supernatants were filtered through 0.45 µm pore size nylon syringe filters (25 mm diameter) and subjected to microextraction by packed sorbent (MEPS) for further clean-up.

The MEPS protocol employed a 100 μL HPLC syringe (SGE Analytical Science, Melbourne, VIC, Australia) equipped with a barrel insert and needle assembly (BIN) containing M1 (C8 + SCX mixed mode) sorbent. The sorbent was activated with 200 μL of methanol, followed by equilibration with 200 μL of ultrapure water. A loading solution comprising 100 μL of the diluted methanolic extract, 100 μL of ultrapure water, and 5 μL of IS working solution was drawn and discharged into the syringe barrel. The sorbent was then washed sequentially with 100 μL of water and 100 μL of a water/methanol mixture (90:10), followed by elution with 500 μL of methanol. The eluate was evaporated under vacuum, reconstituted in 100 μL of a 70:30 (*v*/*v*) mixture of components A and B, and injected into the HPLC-DAD-MS/MS system.

### 4.6. Experimental Design, Data Processing, and Statistical Analysis

#### 4.6.1. Experimental Design

A completely randomized factorial (7 × 2 × 2) design (CRD) was adopted to investigate the effects of three experimental factors on the traits of Madagascar periwinkle: LED light spectrum (seven levels: WW-P, R, B, RB, RBFR, CW-P, and W-CM), plasma-activated water (PAW; two levels: “YES” and “NO”), and sampling time (T; two levels: T1 and T2). The experimental design therefore comprised 14 treatments resulting from the combination of the seven LED spectra with the two PAW levels, each assessed at two destructive experimental sampling times (28 TCC).

The experiment was conducted in identical growth chambers with the same internal dimensions, cultivation-rack configuration, and environmental control system. Each growth chamber was equipped with four cultivation racks, and each rack comprised three vertically arranged shelf levels. Each shelf level was subdivided into two shelf compartments using opaque black plastic panels. The panels provided a physical separation between adjacent compartments and prevented light interference between different LED treatments, ensuring that each compartment received only its assigned light treatment.

The resulting configuration provided a total of 72 shelf compartments across the growth chambers. Each shelf compartment was designed to accommodate 24 plants and was considered an experimental unit (replicate), as all plants within a given compartment received the same LED × PAW treatment combination throughout the experiment. Of the 72 available shelf compartments, 70 were used for the experiment, whereas two compartments were left unused to maintain the predefined experimental layout and spatial separation among the treatment areas.

The 70 experimental units were randomly assigned to the 14 LED × PAW treatments, with five independent experimental-unit replicates assigned to each treatment combination. Thus, the final experimental design comprised 70 independent experimental units corresponding to 14 LED × PAW treatments × 5 replicates. The treatment combinations and their corresponding replicates were randomly distributed among the 70 experimental units across the growth chambers and their respective racks and shelf levels.

At each sampling time, five whole plants were destructively sampled from each replicate shelf compartment for biomass determination and subsequent alkaloid analysis. At T1, five plants were collected from each replicate shelf compartment, whereas at T2, five additional plants, different from those sampled at T1, were collected from the same replicate shelf compartments. Thus, destructive sampling was performed at each sampling time using different plants, while the assigned LED × PAW treatment remained unchanged throughout the experiment.

Depending on the characteristics of the LED system used for each spectral treatment, each shelf compartment was equipped with either one or two LED fixtures. The longer Toplight fixtures, characterized by a broader spatial light distribution, were used as single fixtures because they provided sufficient coverage of the experimental area. Conversely, two smaller Combo fixtures were used where required to ensure homogeneous illumination across the shelf compartment. Thus, the use of one versus two fixtures reflected the physical characteristics and spatial coverage requirements of the respective LED systems and did not represent additional experimental replicates.

Environmental conditions were carefully controlled, and environmental uniformity among shelf compartments across the growth chambers was assessed following the procedure previously applied by Quadri et al. [19] for Madagascar periwinkle indoor cultivation. This procedure adapts the original approaches developed by Barbaresi et al. [88,89] for the assessment of temperature and relative humidity and additionally incorporates the evaluation of light-distribution uniformity. These assessments were performed both before the experiment through preliminary tests to verify the homogeneity of environmental conditions and during the experiment to confirm that this uniformity was maintained throughout the experimental period.

Uniformity of light distribution across all shelf compartments of the different growth chambers was assessed in two phases. First, a preliminary assessment was performed in the absence of plants using DIALux lighting design software 13.2 (DIAL GmbH, Lüdenscheid, Nordrhein-Westfalen, Germany) to model and optimize the spatial distribution of light according to fixture positioning and growth-chamber geometry. Subsequently, in situ measurements were performed with plants present in the growth chambers using the quantum sensors described previously in order to verify the light distribution at canopy height and adjust LED intensity to obtain a consistent photosynthetic PPFD of 150 µmol m^−2^ s^−1^ across the experimental shelf compartments.

Temperature stability was assessed at both chamber and shelf levels, including the evaluation of potential heat emission from the LED fixtures. The heat generated by the fixtures was negligible and did not produce measurable temperature differences among the experimental units, with the chamber temperature maintained at a target value of 23 °C.

Temperature and relative humidity uniformity were assessed by positioning two sensors on each experimental shelf compartment, and measurements were recorded at three-minute intervals. After data cleaning, differences in temperature and relative humidity between pairs of experimental units were calculated at each time point, and the corresponding z-values were determined according to the cited methodology.

The resulting z-values were compared with two reference thresholds: the identity reference value (IRV), which accounts for sensor resolution and accuracy and defines the level below which measurements can be considered identical, and the acceptable reference value (ARV), which represents the maximum difference considered acceptable among environmental zones according to the performance of the environmental control system and the experimental requirements. The IRV values were 0.7 °C for temperature and 4.7% for relative humidity, whereas the corresponding ARV values were 1.5 °C and 5%, respectively. It is important to emphasize that these ARV thresholds are substantially more stringent than the environmental variability typically reported for the natural habitat of the Madagascar periwinkle. These thresholds were therefore used as predefined criteria for assessing environmental equivalence among experimental units. Experimental units were considered environmentally equivalent when, for both temperature and relative humidity, all calculated z-values remained below the corresponding ARV.

The environmental uniformity assessments conducted both before and during the experiment showed that temperature and relative humidity remained homogeneous and stable across the shelf compartments of the growth chambers, with no significant environmental heterogeneity according to the predefined criteria. Thus, according to the predefined criteria, no significant environmental variability was detected either among the shelf compartments within the same growth chamber or among shelf compartments belonging to different growth chambers. Based on these assessments, no blocking structure was adopted, and the shelf compartment was not included as a random effect in the statistical model.

#### 4.6.2. Measured Traits and Alkaloid Yield Calculation

Madagascar periwinkle traits analyzed included the following:

1. Leaf, root and total (leaf + root) dry DM;
2. Leaf and root concentrations of VDL and CAT;
3. Leaf and root yields of VDL and CAT;
4. Total (leaf + root) yields of VDL and CAT.

In order to calculate yields (µg plant^−1^), the DMs (g plant^−1^) were multiplied by their concentration (µg g^−1^ DM).

#### 4.6.3. Baseline (T0) Data Handling

Data collected at T0 (before treatment) were analyzed by calculating the arithmetic mean of yields, concentrations, and DMs from five sampled plants. Consequently, T0 data were used exclusively for baseline physiological characterization prior to treatment application and were not included in the factorial experimental design or statistical models; therefore, they were not subjected to post-hoc tests (i.e., analyses based solely on arithmetic means). To ensure consistency in the temporal framework, T0 is included in figures reporting the main effects of sampling time together with T1 and T2, whereas in figures reporting significant interactions (only the LED light spectrum:sampling time and PAW:sampling time interactions were found to be significant for some variables; see Section 2.2), it is represented using the labels WW-P:T0 and NO:T0 for the respective interaction terms. Accordingly, T0, WW-P:T0, and NO:T0 represent the same pre-treatment condition displayed in different analytical contexts.

#### 4.6.4. Statistical Analysis and Data Visualization

The data analysis for this study was conducted using RStudio version 4.3.3 [90]. Linear ANOVA models were used to assess the effects of LED light spectrum, PAW treatment, and sampling time (T) and their interaction on the previously described traits (considered as response variables in the models). At each sampling time, the measurements obtained from the five plants destructively sampled within each shelf-compartment experimental unit were averaged, resulting in one value per experimental unit. Therefore, the statistical analyses were based on five independent shelf-compartment replicates for each LED × PAW treatment combination at each sampling time. Normality and homogeneity of variances were checked using quantile-quantile and residual plots, respectively. Variables not meeting these assumptions were transformed: logarithmic transformation for leaf CAT concentration, leaf VDL yield, leaf CAT yield, total VDL yield, total CAT yield; Box–Cox transformation (λ = 0.26) for root CAT yield using the ‘make.tran’ function from the ‘emmeans’ package [91]. *p*-values were obtained using the ANOVA function (Type III) from the car package [92]. Estimated marginal means (EMMs) were obtained by applying linear models to the emmeans function from the emmeans package [91] to calculate estimated marginal means (EMMs). When necessary, EMMs were back-transformed to their original scale for interpretation using the type = ‘response’ argument. For multiple pairwise comparisons (more than two means), the cld function from the multcomp package was employed to generate compact letter displays (CLDs) of EMMs [93], applying the Benjamini–Hochberg (BH) adjustment. In contrast, for simple pairwise comparisons (between two means), *t*-tests were conducted using the same cld function on the EMMs to generate CLDs. All statistical analyses were performed at a significance level of 0.05. Data visualization was carried out with the ggplot function from the ggplot2 package [94], and results are presented as means ± SE, except for the pH (Section 2.1.2) and RONS concedntrations (Section 2.1.3) in PAW, which were expressed as mean ± SD.

## 5. Conclusions

This study demonstrated that the LED light spectrum and PAW, together with sampling time, significantly influence biomass accumulation and VDL and CAT production in Madagascar periwinkle grown under controlled conditions in a CPPS.

RB light consistently produced the highest VDL and CAT yields, with total yields significantly exceeding those obtained under all spectral treatments except W-CM, where comparable values were observed. LMDI decomposition clearly identified leaf DM as the main driver of total yield increases under RB light, indicating that biomass production, rather than concentration changes, was the dominant factor at the whole-plant level. Notably, although RB and W-CM resulted in comparable total yields, the use of R and B wavelengths, corresponding to major absorption regions of photosynthetic pigments, highlights RB as a promising spectral configuration for maximizing biomass and alkaloid production under the tested CPPS conditions. Compared with broad-spectrum W LEDs, which encompass a wider range of wavelengths including G and FR regions, the use of targeted R and B wavelengths may provide an advantage in terms of photosynthetic photon-use efficiency and may therefore constitute a potentially more energy-efficient option for CPPS when comparable alkaloid yields are achieved.

PAW application significantly enhanced VDL yields as well as leaf and total CAT yields, while also generally promoting root CAT yield (especially at later developmental stages). However, its mode of action was organ-dependent and physiologically complex. PAW promoted yield increases through different mechanisms, with concentration-driven effects prevailing for VDL, whereas CAT responses were more strongly associated with biomass accumulation, as shown by the LMDI analysis. These results indicate that PAW may act through multiple physiological pathways, likely involving ROS/RNS-mediated signaling, improved nitrogen availability, and modulation of specialized metabolism.

Sampling time (harvest period) had the strongest overall effect on plant performance. A marked increase in biomass and in leaf, root, and total VDL and CAT yields was observed from T0 to T2, indicating that developmental stage is a critical determinant of TIA accumulation. LMDI analysis consistently showed that leaf biomass was the dominant contributor to total yield increases over time, reinforcing the central role of leaf growth in driving whole-plant alkaloid productivity.

The combined use of RB LED light and PAW, together with harvesting at a later growth stage (T2), represents the most effective strategy for enhancing VDL and CAT production, as it optimized both total VDL and CAT yields.

These results demonstrate that precise manipulation of LED light spectrum, PAW, and harvest timing in CPPS can significantly optimize the production of high-value pharmaceutical compounds in Madagascar periwinkle. The integration of LMDI decomposition enabled quantitative identification of the physiological drivers underlying yield variation, providing a robust framework for interpreting complex multi-factor plant responses.

Despite the robustness of the experimental design and the large dataset generated, limitations should be acknowledged. The LMDI approach identifies physiological con-tributors to yield variation but remains correlative and does not directly confirm the underlying molecular or enzymatic mechanisms. Likewise, the accumulation of VDL and CAT in roots cannot distinguish between local biosynthesis and long-distance transport, which should be investigated using transcriptomic, proteomic, trans-porter-level, and isotope-labeling approaches. The study also evaluated multiple LED spectra at a single PPFD level and a single PAW composition and exposure duration; future experiments should therefore assess spectrum:light-intensity interactions and different PAW formulations and treatment durations. Importantly, electrical energy consumption, photon efficacy, operating costs, and energy and water use per unit of biomass or alkaloid yield were not directly assessed. Thus, although RB light may offer a potential advantage in terms of photosynthetic photon-use efficiency, its relative energy efficiency cannot be established from the present data and requires experimental validation. Similarly, although the present PAW production and backpack foliar ap-plication procedure provides a practical approach for treating plant batches, its broader economic and resource-use implications were not quantitatively evaluated. Future studies at larger cultivation scales should therefore incorporate energy-, water-, and cost-normalized analyses, including energy and water consumption per unit of biomass or alkaloid yield, to further evaluate the practical feasibility of translating the present RB–PAW approach to semi-commercial or industrial CPPS production.

## Figures and Tables

**Table 1:** Three-way ANOVA results. Effect of the LED light spectrum (*L*), plasma-activated water (*PAW*), and sampling time (*T*) on dry masses (DMs), and vindoline (VDL) and catharanthine (CAT) concentrations and yields in Madagascar periwinkle. Significant differences at *p* ≤ 0.05 (*), *p* ≤ 0.01 (**), and *p* ≤ 0.001 (***); ns = not significant differences.

Variables	L	PAW	T	L:PAW	L:T	PAW:T	L:PAW:T
Leaf DM (g plant^−1^)	**	**	***	ns	ns	ns	ns
Root DM (g plant^−1^)	***	**	***	ns	ns	ns	ns
Total DM (g plant^−1^)	***	***	***	ns	ns	*	ns
Leaf VDL concentration (µg g^−1^ DM)	*	***	***	ns	ns	**	ns
Leaf CAT concentration (µg g^−1^ DM)	*	**	***	ns	ns	ns	ns
Root VDL concentration (µg g^−1^ DM)	***	***	***	ns	*	ns	ns
Root CAT concentration (µg g^−1^ DM)	*	ns	***	ns	ns	ns	ns
Leaf VDL yield (µg plant^−1^)	***	***	***	ns	ns	ns	ns
Leaf CAT yield (µg plant^−1^)	***	***	***	ns	ns	ns	ns
Root VDL yield (µg plant^−1^)	***	***	***	ns	ns	ns	ns
Root CAT yield (µg plant^−1^)	***	**	***	ns	ns	*	ns
Total VDL yield (µg plant^−1^)	***	***	***	ns	ns	ns	ns
Total CAT yield (µg plant^−1^)	***	***	***	ns	ns	ns	ns

**Table 2:** Summary of the experimental treatments.

Treatments	LED Light Spectrum	PAW	Sampling Time (T)	TCC
WW-P (CK)	Phosphor-converted warm white; CCT 3221 K; 50% R, 13% B, 35% G, 2% FR	NO	T1 T2	WW-P:NO:T1 WW-P:NO:T2
WW-P + PAW	Same spectrum as WW-P	YES	T1 T2	WW-P:YES:T1 WW-P:YES:T2
R	Monochromatic red; peak 662 nm	NO	T1 T2	R:NO:T1 R:NO:T2
R + PAW	Same spectrum as R	YES	T1 T2	R:YES:T1 R:YES:T2
B	Monochromatic blue; peak 446 nm	NO	T1 T2	B:NO:T1 B:NO:T2
B + PAW	Same spectrum as B	YES	T1 T2	B:YES:T1 B:YES:T2
RB	86% R, 14% B (6:1); peaks 662 and 446 nm	NO	T1 T2	RB:NO:T1 RB:NO:T2
RB + PAW	Same spectrum as RB	YES	T1 T2	RB:YES:T1 RB:YES:T2
RBFR	75% R, 21% B, 4% FR; peaks 662, 445, and 727 nm	NO	T1 T2	RBFR:NO:T1 RBFR:NO:T2
RBFR + PAW	Same spectrum as RBFR	YES	T1 T2	RBFR:YES:T1 RBFR:YES:T2
CW-P	Phosphor-converted cool white; CCT 4877 K; 30% R, 21% B, 47% G, 2% FR	NO	T1 T2	CW-P:NO:T1 CW-P:NO:T2
CW-P + PAW	Same spectrum as CW-P	YES	T1 T2	CW-P:YES:T1 CW-P:YES:T2
W-CM	Color-mixed white; 53% R, 29% B, 17% G, 1% FR	NO	T1 T2	W-CM:NO:T1 W-CM:NO:T2
W-CM + PAW	Same spectrum as W-CM	YES	T1 T2	W-CM:YES:T1 W-CM:YES:T2

Abbreviations: CK, control; B: blue light; G: green light; R: red light; FR: far-red light; CCT, correlated color temperature; PAW, plasma-activated water; T1 and T2, experimental sampling times; TCC, treatment combination code.

## Data Availability

Data will be made available on request.

## Supplementary Materials

The following supporting information can be downloaded at https://www.mdpi.com/article/10.3390/plants15192970/s1. Table S1: Nomenclature for treatment combinations for the two-way interactions. Figure S1: Effect of sampling time on total dry mass (DM) in Madagascar periwinkle. Table S2: Definition of controls for main effects and interactions in the factorial experimental design. Figure S2: Effect of sampling time on leaf VDL concentration in Madagascar periwinkle. Figure S3: Effect of sampling time on root VDL concentration in Madagascar periwinkle. Figure S4: Total average VDL and CAT concentrations in Madagascar periwinkle leaves and roots. Figure S5: Effect of sampling time on root CAT yield in Madagascar periwinkle. Figure S6: Periwinkle plants treated with different LED light spectra. Figure S7: Treatment with PAW by spraying method using a battery-powered backpack atomizer. Spreadsheet S1: LMDI-based logarithmic decomposition of total dry mass (DM) as well as vindoline (VDL) and catharanthine (CAT) yields under light spectrum, PAW, and sampling-time comparisons. Supplementary text section: LMDI (Logarithmic Divisia Index)-based logarithmic decomposition analysis.
