# Supplementary material for: Red–Blue LED Light and Plasma-Activated Water Enhance Vindoline and Catharanthine Yields in Catharanthus roseus Grown in a Closed Plant Production System
Source: Plants (Basel). 2026 Sep 29;15(19):2970. doi: 10.3390/plants15192970 (PMC13667196; doi:10.3390/plants15192970)
Supplement: Supplementary file 1 [file plants-15-02970-s001.zip › Supplementary materials.pdf]

Supplementary materials

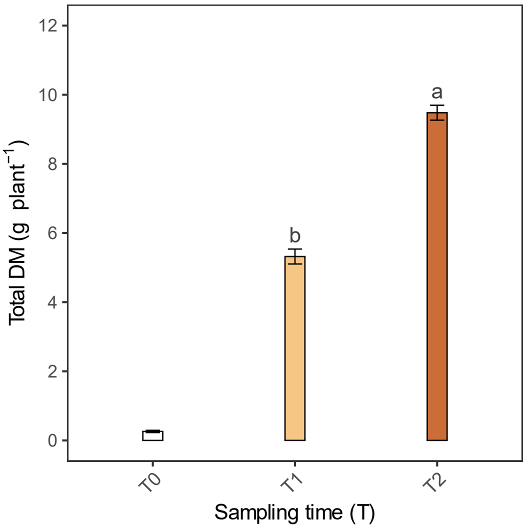

Figure S1. Effect of sampling time (T) on total dry mass (DM) in Madagascar periwinkle. T1 = 47 DAP; T2 = 55 DAP; T0 (1 DAP) represents the reference baseline condition prior to the application of any experimental treatment and is labelled in this form for graphical and terminological consistency within the sampling time effect structure; therefore, T0 is not part of the post-hoc comparisons. Bars represent SE (*n* = 5). Different letters indicate significant differences ($p \leq 0.05$) according to t-test.

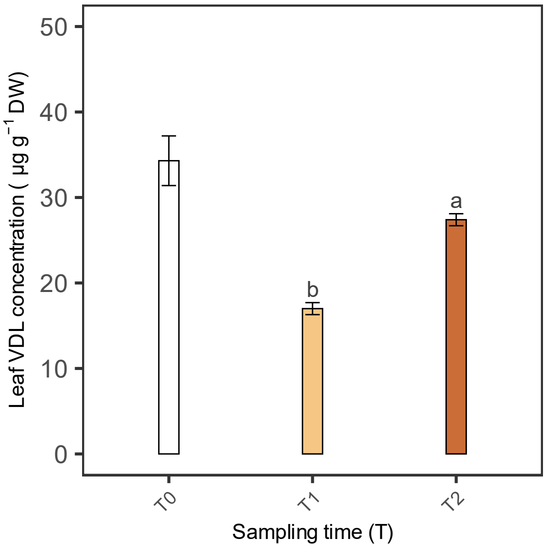

Figure S2. Effect of sampling time (T) on leaf VDL concentration in Madagascar periwinkle. T1 = 47 DAP; T2 = 55 DAP; T0 (1 DAP) represents the reference baseline condition prior to the application of any experimental treatment and is labelled in this form for graphical and terminological consistency within the sampling time effect structure; therefore, T0 is not part of the post-hoc comparisons. Bars represent SE (*n* = 5). Different letters indicate significant differences ($p \leq 0.05$) according to t-test.

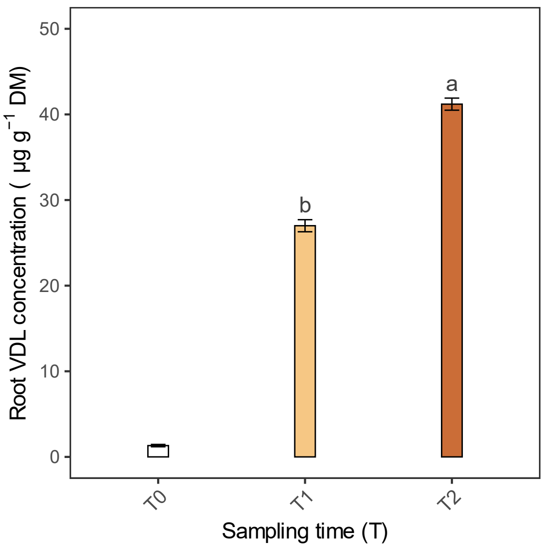

Figure S3. Effect of sampling time (T) on root VDL concentration in Madagascar periwinkle. T1 = 47 DAP; T2 = 55 DAP; T0 (1 DAP) represents the reference baseline condition prior to the application of any experimental treatment and is labelled in this form for graphical and terminological consistency within the sampling time effect structure; therefore, T0 is not part of the post-hoc comparisons. Different letters indicate significant differences ($p \leq 0.05$) according to t-test.

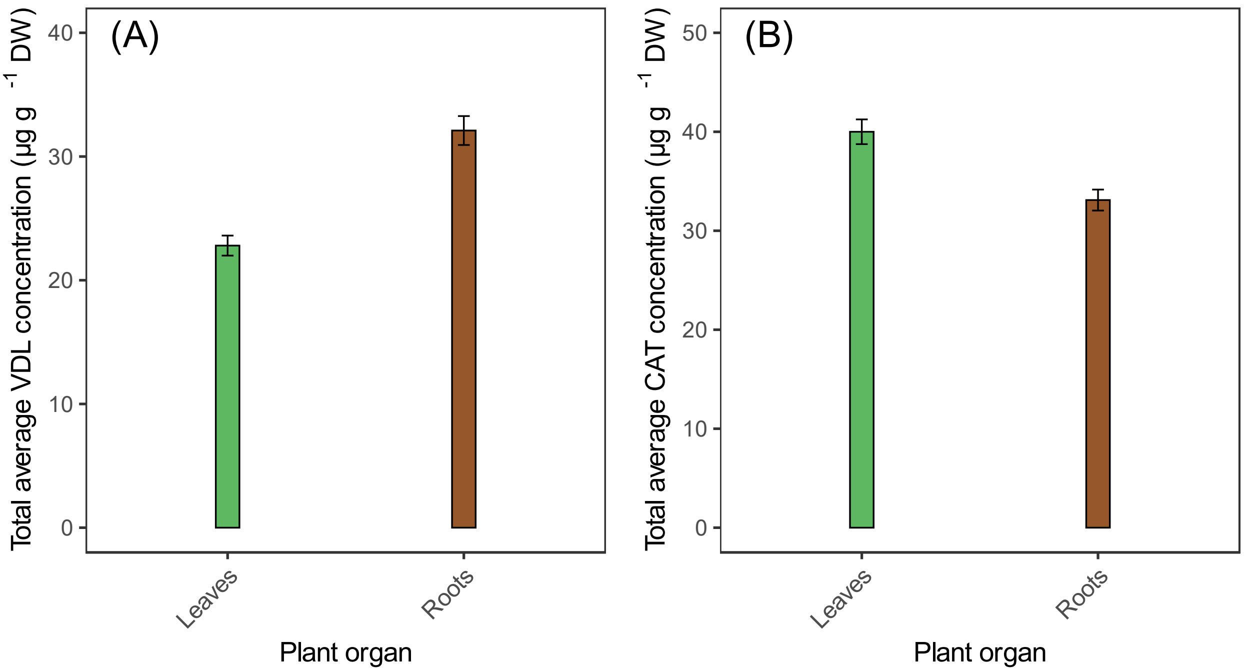

Figure S4. Total average VDL and CAT concentrations in Madagascar periwinkle leaves and roots. (**A**) Total average VDL concentration. (**B**) Total average CAT concentration. The green bars represent concentrations in leaves, whereas the brown bars represent concentrations in roots.

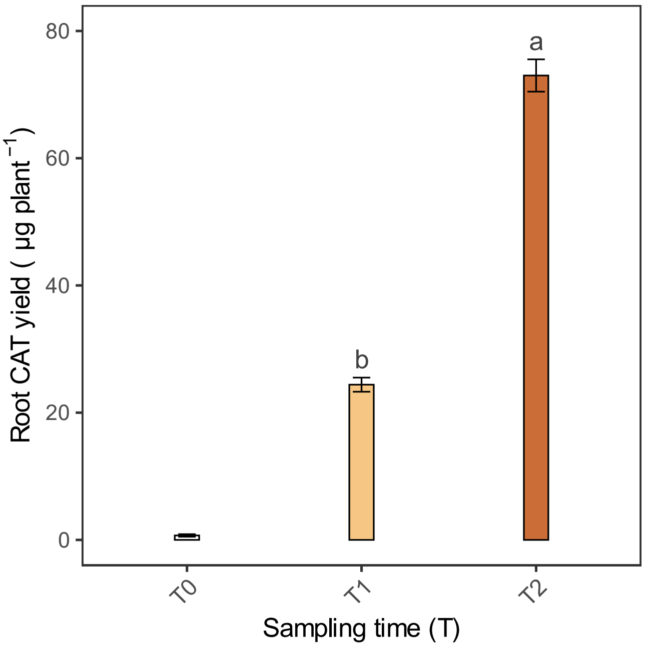

Figure S5. Effect of sampling time (T) on root CAT yield in Madagascar periwinkle. T1 = 47 DAP; T2 = 55 DAP; T0 (1 DAP) represents the reference baseline condition prior to the application of any experimental treatment and is labelled in this form for graphical and terminological consistency within the sampling time effect structure; therefore, T0 is not part of the post-hoc comparisons. Bars represent SE (n = 5). Different letters indicate significant differences ($p \leq 0.05$) according to t-test.

Table S1. Nomenclature for treatment combinations for the two-way interactions

Combination	Description
WW-P:T1	plants treated with phosphor-converted warm white light, regardless of PAW, sampled at T1
WW-P:T2	plants treated with phosphor-converted warm white light, regardless of PAW, sampled at T2
R:T1	plants treated with red light, regardless of PAW, sampled at T1
R:T2	plants treated with red light, regardless of PAW, sampled at T2
B:T1	plants treated with blue light, regardless of PAW, sampled at T1
B:T2	plants treated with blue light, regardless of PAW, sampled at T2
RB:T1	plants treated with red-blue light, regardless of PAW, sampled at T1
RB:T2	plants treated with red-blue light, regardless of PAW, sampled at T2
RBFR:T1	plants treated with red-blue-far red light, regardless of PAW, sampled at T1
RBFR:T2	plants treated with red-blue-far red light, regardless of PAW, sampled at T2
CW-P:T1	plants treated with phosphor-converted cool white light, regardless of PAW, sampled at T1
CW-P:T2	plants treated with phosphor-converted cool white light, regardless of PAW, sampled at T2
W-CM:T1	plants treated with color-mixed white light, regardless of PAW, sampled at T1
W-CM:T2	plants treated with color-mixed white light, regardless of PAW, sampled at T2
NO:T1	plants not treated with PAW, regardless of light spectrum, sampled at T1
NO:T2	plants not treated with PAW, regardless of light spectrum, sampled at T2

YES:T1	plants treated with PAW, regardless of light spectrum, sampled at T1
YES:T2	plants treated with PAW, regardless of light spectrum, sampled at T2

Link to the official water-quality data provided by the public water supplier.

https://eng.gruppohera.it/group_eng/sustainability/thematic-reports/in-good-waters/quality-data

Table S2. **Definition of controls for main effects and interactions in the factorial experimental design**. Single effects and interactions are listed in the first column. Controls for main effects are defined as baseline levels of each factor independently of the others, while controls for interactions (two-way and three-way) are defined as specific combinations of factor levels serving as reference points for interpreting combined effects. These controls and reference points served as baselines or references for statistical analyses.

Main effect/interaction	Control	Description
LED light spectrum	WW-P	plants treated with phosphor-converted warm white light regardless of PAW treatment and sampling time; baseline level of the LED light spectrum factor
PAW	NO	plants not treated with PAW regardless of LED light spectrum and sampling time; baseline level of the PAW factor
Sampling time (T)	T1	plants sampled at the first sampling time (T1) regardless of LED light spectrum and PAW; baseline level of the sampling time factor
LED light spectrum:sampling time (T)	WW-P:T1	plants treated with phosphor-converted warm white light, regardless of PAW, sampled at T1; primary reference point for the comparison among all light LED spectrum:sampling time (T) combinations and reference point for T1 under equal sampling times
	WW-P:T2	plants treated with phosphor-converted warm white light, regardless of PAW, sampled at the second sampling time (T2); reference point for T2 under equal sampling times
PAW:Sampling time (T)	NO:T1	plants not treated with PAW, regardless of light spectrum, sampled at T1; primary reference point for the comparison among all PAW:sampling time (T) combinations and reference point for T1 under equal sampling times
	NO:T2	plants not treated with PAW, regardless of LED light spectrum, sampled at T2; reference point for T2 under equal sampling times
LED light spectrum:PAW	WW-P:NO	plants treated with phosphor-converted warm white light without PAW, regardless of sampling time; considered the overall control

		(CK) of the experiment regardless of sampling time
LED Light spectrum:PAW:sampling time (T)	WW-P:NO:T1	plants treated with phosphor-converted warm white light without PAW, sampled at T1; primary reference point for the whole comparison (all LED light specrtrum:PAW:sampling time combinations) and reference for T1 under equal sampling times
	WW-P:NO:T2	plants treated with phosphor-converted warm white without PAW, sampled at T2; reference point for T2 under equal sampling times

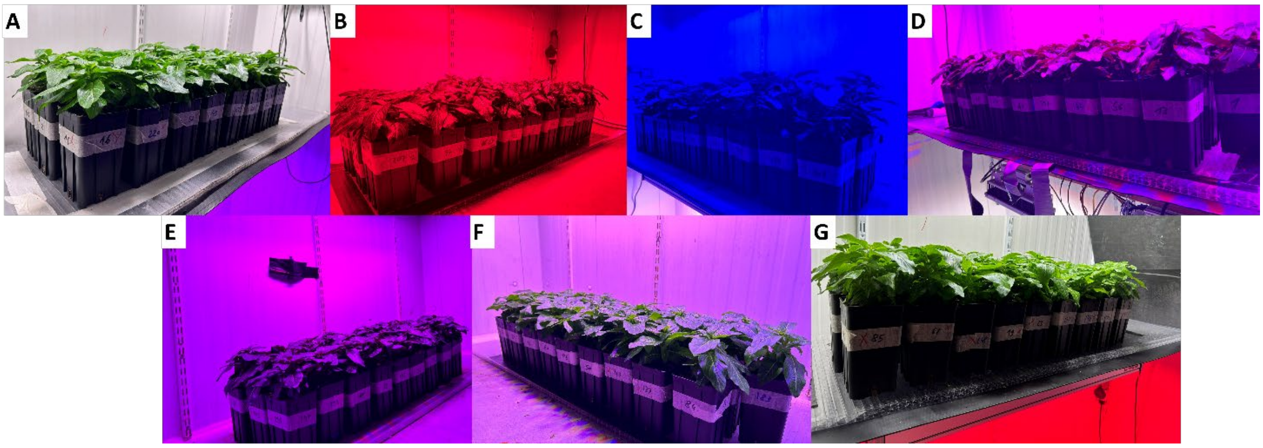

Figure S6. Periwinkle plants treated with the different LED light spectra. Periwinkle plants were exposed under seven LED light spectra: phosphor-converted warm white “WW-P (control)” (A), red “R”(B), blue “B”(C), a mixture of R and B (RB) (D), a mixture of R, B and Far-red (FR) “RBFR” (E), phosphor-converted cool white “CW”(F) and color-mixed white “W-CM” (G).

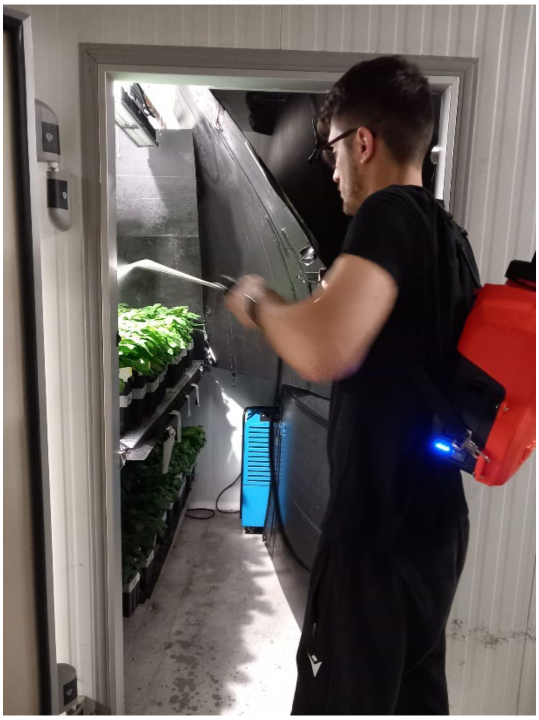

Figure S7. Treatment with PAW by spraying method using a battery-powered backpack atomizer. Plants assigned to the PAW treatments were sprayed for 20 minutes on the aerial parts. Plants in the non-PAW treatments were sprayed for 20 minutes with tap water (control), using the same method as for PAW.

LMDI (Logarithmic Divisia Index) -based logarithmic decomposition analysis

1. General objective of the analysis

The general objective of this analysis is to identify which structural and physiological variables have contributed most to the increase in total dry mass (DM) and in the yields of vindoline (VDL) and catharanthine (CAT) observed under the reference conditions, namely those that exhibited the highest mean yield values within the different considered experimental comparisons.

In particular, the aim was to understand the following:

a) For ***multiplicative variables*** (**leaf and root yields of VDL and CAT**), whether the observed increase in yields under the reference conditions was mainly due to an increase in the dry mass of the organ (DM), or to an increase in the concentration of the compound (C) under the same reference conditions.

General formula

$$Y_{m,o} = \prod_{i=1}^{n} X_i$$

where:

- $Y_{m,o}$ = yield of metabolite mmm in organ ooo (leaf or root yield of VDL or CAT)
- m = metabolite considered (VDL or CAT)
- o= organ considered (leaves or roots)
- X_i = i-th multiplicative factor contributing to the yield of metabolite m in organ ooo:
  - $i = 1 \rightarrow X_{1,o} = DM_o$ = dry mass of organ ooo (leaf or root dry mass)
  - $i = 2 \rightarrow X_{2,m,o} = C_{m,o}$ = concentration of metabolite m in organ o (leaf or root concentration of VDL or CAT)

- i=1,…,n= index of the multiplicative factors considered
- n = total number of multiplicative factors (in the present case, n = 2)

Specific formulas used in the present study:

$$Leaf\ VDL\ yield = Leaf\ DM\ x\ Leaf\ VDL\ concentration$$

$$Root\ VDL\ yield = Root\ DM\ x\ Root\ VDL\ concentration$$

$$Leaf\ CAT\ yield = Leaf\ DM\ x\ Leaf\ CAT\ concentration$$

$$Root\ CAT\ yield = Root\ DM\ x\ Root\ CATconcentration$$

b) For ***additive variables*** (**total dry mass, total yields of VDL and CAT**), whether the observed increase under the reference conditions could be attributed predominantly:

- to the increase in leaf dry mass or to the increase in root dry mass under the same reference conditions (total dry mass expressed as the sum of organ-specific dry masses – ***structural or organ-specific approach*** linked to *total dry mass*)
- to the increase in leaf VDL and CAT yield or to the increase in root VDL and CAT yield under the same reference conditions (total dry mass/total yield expressed as the sum of organ-specific dry masses/yields – **structural or organ-specific approach** linked to *total alkaloid yields*), independently of the physiological factors that determine it (mass or concentration)
- to variations in one of the individual components of alkaloid yields in the case of total VDL and CAT yields, namely variations in dry mass or compound concentration in the individual organs under the same reference conditions (total VDL and CAT yield expressed as the sum of products of mass and concentrations **– physiological approach linked** to *total alkaloid yields*)

General formula of total dry mass as the sum of organ-specific dry masses (structural or organ-specific approach related to biomass)

$$DM_{tot} = \sum_{i=1}^{n} (DM_i)$$

where:

- DM_{tot}= total dry mass
- i = index of the additive component corresponding to the considered organ

i=1 → leaves

i= 2 → roots

- DM_i = dry mass of the i-th organ (leaf or root dry mass)
- *n* = total number of additive components (organs) considered (in the present case n = 2)

General formula of total yields as the sum of organ-specific yields (structural or organ-specific approach related to alkaloid yields)

$$Y_{tot,m} = \sum_{i=1}^{n}(Y_{i,m})$$

where:

- $Y_{tot,m}$= total yield of metabolite *m* (total yield of VDL or CAT)
- *m* = metabolite considered (VDL or CAT)
- *i* = index of the additive component corresponding to the considered organ

i=1 → leaves

i= 2 → roots

- $Y_{i,m}$ = yield of metabolite mmm in the i-th organ (leaf or root yield of VDL or CAT)
- *n* = total number of additive components (organs) considered (in the present case *n* = 2)

Specific formulas used in the present study:

$$Total\ DW = Leaf\ DM + Root\ DM$$

$$Total\ VDL\ yield = Leaf\ VDL\ yield + Root\ VDL\ yield$$

$$Total\ CAT\ yield = Leaf\ CAT\ yield + Root\ CAT\ yield$$

General formula of total yields as the sum of products of masses and concentrations (physiological approach)

$$Y_{tot,m} \sum_{i}^{n} (Y_{i,m}) = Y_{tot,m} = \sum_{i}^{n} (DM_i \cdot C_{i,m})$$

where:

- Y_{tot}= total yield of metabolite m (total yield of VDL or CAT)
- m = metabolite considered (VDL or CAT)
- DM_i = dry mass of the *i-th* organ (leaf or root dry mass)
- C_i = concentration of metabolite m in the i-th organ (leaf or root concentration of VDL or CAT)
- *i*= index of the considered organ:
  - *i*=1 → leaves
  - *i*= 2 → roots
- n = total number of organs included in the sum (in the present case, n = 2)

Specific formulas used in the present study:

$$Total\ VDL\ yield = (Leaf\ DM\ x\ Leaf\ VDL\ concentration) + (Root\ DM\ x\ Root\ VDL\ concentration)$$

$$Total\ CAT\ yield = (Leaf\ DM\ x\ Leaf\ CATconcentration\ ) + (root\ DM\ x\ Root\ CAT\ concentration)$$

In summary, additive variables were analysed at two complementary levels:

- a **structural or organ-specific level** (leaf and root) linked to **total dry biomass or total alkaloid yields**
- a **physiological level** (DM and C) linked **exclusively to total alkaloid yields**

in order to obtain a complete and coherent representation of the factors driving the increase in total dry mass and in VDL and CAT yields.

2. Decomposition methods used

2.1 Logarithmic decomposition (LMDI)

The analysis of the contributions of the individual variables to total dry mass and to total alkaloid yields was conducted according to a systematic analysis of sources of variation, using logarithmic decomposition techniques and, specifically, a LMDI (*Logarithmic Mean Divisia Index*)-based approach. These techniques, originally developed in the economic and energy fields [1–6] and subsequently extended to agronomy, plant physiology and ecophysiology [7–10], allow the disaggregation of an observed overall variation (*e.g.*, an increase in total dry mass or total alkaloid yield) into the partial contributions of the elemental factors that determine it. In other words, they allow a complex change (resulting from the interaction of multiple components) to be mathematically translated into a sum of interpretable effects, each associated with a single structural factor (*e.g*. leaf and root dry mass for total dry mass; leaf and root yield of VDL and CAT for total yield of these alkaloids) or a biological/physiological factor (*e.g*. dry mass and concentration for individual leaf or root alkaloid yields). In particular, the LMDI approach was used in its simplified multiplicative version (simple logarithmic decomposition) and additive form [1–6].

2.2 Scope of application of logarithmic decompositions

Logarithmic decompositions were applied to three types of comparisons:

- Comparison between LED light spectra
- Comparison between PAW treatments
- Comparison between sampling times (T)
- Comparisons between PAW:sampling time combinations

In each comparison, the analysis aims to verify the consistency of the contribution of individual factors (leaf and root, or DM and C) to the variation in biomass or alkaloid yield, identifying the main drivers of the observed differences.

2.3 Selection of reference conditions and sign convention

In all comparisons, the reference condition (*ref*) was the one associated with the highest yield within the specific comparison considered:

- LED light spectra (variables in which the effect of the LED spectrum was significant and any interactions with it were not significant): RB vs all other spectra (WW-P, R, B, RBFF, CW-P, W-CM)
- PAW (variables in which the effect of PAW was significant and any interactions with it were not significant): YES vs NO
- Sampling times (T): (*a*) T_2 vs T_1 and T_0; (*b*) T_2 vs T_0, (c) T_2 vs T_1
- Combination PAW treatment: sampling time (variables in which the PAW:sampling time interaction was significant): (*a*) YES:T2 vs all other combinations including the baseline NO:T0 (NO:T2, YES:T1, NO:T1, NO:T0); (*b*) YES:T2 vs all other combinations excluding the baseline NO:T0 (NO:T2, YES:T1, NO:T1); (*c*) YES:T2 vs NO:T2; (*d*) YES:T1 vs NO:T1; (*e*) YES:T2 vs YES:T1; (*f*) YES:T2 vs NO:T1, YES:T1 and NO:T0; (*g*) YES:T2 vs NO:T1 and YES:T1; (*h*) NO:T2 vs NO:T1, YES:T1 and NO:T0; (*i*) NO:T2 vs NO:T1 and YES:T1

For each variable (Xi, $Y_{i,m}$, W_i , W_{tot}, $Y_{m,o}$ *e* $Y_{tot,m}$), the logarithmic variation between a reference condition (*ref*) and a comparison condition (*cond*) was calculated as:

$$\Delta \ln(X_i) = \ln(X_{i,ref}) - \ln(X_{i,cond})$$

$$\Delta \ln(Y_{i,m}) = \ln(Y_{i,m,ref}) - \ln(Y_{i,m,cond})$$

$$\Delta \ln(DM_i) = \ln(DM_{i,ref}) - \ln(DM_{i,cond})$$

$$\Delta \ln(DM_{tot}) = \ln(DM_{tot,ref}) - \ln(DM_{tot,cond})$$

$$\Delta \ln(Y_{m,o}) = \ln(Y_{m,o,ref}) - \ln(Y_{m,o,cond})$$

$$\Delta \ln(Y_{tot,m}) = \ln(Y_{tot,m,ref}) - \ln(Y_{tot,m,cond})$$

With this convention:

- $\Delta\ln(X_i)$, $\Delta\ln(Y_{i,m})$, $\Delta\ln(DM_{i,})$, $\Delta\ln(DM_{tot,})$, $\Delta\ln(Y_{m,o})$, $\Delta\ln(Y_{tot,m}) > 0 \rightarrow$ the variable is greater in the reference condition

- $\Delta\ln(X_i)$, $\Delta\ln(Y_{i,m})$, $\Delta\ln(DM_{i,})$, $\Delta\ln(DM_{tot,})$, $\Delta\ln(Y_{m,o})$, $\Delta\ln(Y_{tot,m}) < 0$ → the variable is greater in the comparison condition

A positive value of $\Delta\ln(DM_{tot,})$, $\Delta\ln(Y_{m,o})$ e $\Delta\ln(Y_{tot,m})$ therefore indicates that dry mass and alkaloid yield are higher under the reference condition, and the positive contributions of the associated factors represent physiological drivers of the increase in dry mass and alkaloid yield.

2.4 Multiplicative LMDI simplified: simple logarithmic decomposition (multiplicative variables)

In the case of multiplicative variables (leaf and root yields of VDL and CAT), a simplified form of multiplicative LMDI was applied, based on the direct logarithmic decomposition of factors (simple logarithmic decomposition). In this formulation, logarithmic weights are unitary ($\omega_{i,m} = 1$), since each variable Xi (dry mass and concentration) contributes directly and separately to the variation of $Y_{m,o}$, without sub-aggregates among which the total variation must be redistributed.

2.4.1 General mathematical definition

Therefore, thanks to the fundamental logarithmic property:

$$\ln(a \cdot b) = \ln(a) + \ln(b)$$

the relative variation of $Y_{m,o}$ between two conditions *(ref* vs *cond*) can be expressed (decomposed) into the contributions of the individual factors, i.e. as an exact sum of the logarithmic variations of the individual factors:

$$\Delta\ln(Y_{m,o}) = \ln(Y_{m,o,ref}) - \ln(Y_{m,o,cond}) = \sum_{i=1}^{n}[\ln(X_{i,ref}) - \ln(X_{i,cond})] = \sum_{i=1}^{n}\Delta\ln(X_i)$$

where:

- $\Delta\ln(Y_{m,o})$=overall logarithmic variation of the variable $Y_{m,o}$ between the reference condition (*ref*) and the comparison condition (*cond*)

- $\Delta\ln(X_i)$= logarithmic contribution of factor X_i to the total variation.

This approach requires no weighting: the sum of the partial contributions is exactly equal to the total variation of Y:

$$\Delta\ln(Y_{m,o}) = \sum_{i=1}^{n} \Delta\ln(X_i)$$

Logarithmic variation per single comparison

For each individual comparison j (*e.g.*, RB vs R, YES vs NO, T2 vs T1, T2 vs T0; YES:T2 vs NO:T2, etc.) between the *ref* condition and the *cond* condition:

$$\Delta\ln(X_{i,j}) = \ln(X_{i,ref}) - \ln(X_{i,cond,j})$$

$$\Delta\ln(Y_{m,o,j}) = \ln(Y_{m,o,ref}) - \ln(Y_{m,o,cond,j})$$

Weighted mean logarithmic variations

To summarise the results of multiple comparisons (all) for experimental factors with more than two levels (*e.g.*, light spectra and sampling time in the case of T2 vs T1 vs T0), weighted mean logarithmic contributions (logarithmic variations) were also calculated.

However, for comparisons involving experimental factors with only two levels (*e.g.*, PAW treatment: YES/NO) and for pairwise comparisons between specific sampling time points (*e.g.*, T2 vs T1 or T1 vs T0), weighted means could not be calculated; in these cases, individual logarithmic variations were used.

For each variable X_i the weighted mean (WM) of the logarithmic variation associated with each metabolite was calculated as:

$$\Delta\ln(X_i)_{WM} = \frac{\sum_{j=1}^{ncomp}\left|\Delta ln(Y_{m,o})_j\right| \cdot \Delta ln(X_i)_j}{\sum_{j=1}^{ncomp}\left|\Delta ln(Y_{m,o})_j\right|}$$

where:

- j = index of the individual experimental comparison

- n_{comp} = total number of comparisons considered (*e.g.*, n=6 for LED light spectrum: RB vs all the other spectra; n=2 for sampling time considering T2 vs T1 vs T0, n=3 for PAW:sampling time considering YES:T2 vs NO:T2, YES:T1, NO:T1))
- $\Delta ln(X_i)_j$ = logarithmic variation of variable X_i for the individual comparison *j*
- $|\Delta ln(Y_{m,o})_j|$ = weight assigned to each comparison, given by the absolute value of the logarithmic variation of Y_m for the individual comparison *j*

This weighting approach allows greater importance to be attributed to comparisons showing larger total yield variations, ensuring that the weighted mean consistently reflects the relative impact of different experimental comparisons.

Analogously, the weighted mean logarithmic variation of $Y_{m,o}$ was calculated as:

$$\Delta \ln(Y_{m,o})_{WM} = \frac{\sum_{j=1}^{ncomp}|\Delta ln(Y_{m,o})_j| \cdot \Delta ln(Y_{m,o})_j}{\sum_{j=1}^{ncomp}|\Delta ln(Y_{m,o})_j|}$$

where:

- $\Delta ln(Y_{m,0})_j$ = logarithmic variation of the variable Y_m for the individual comparison *j*

Definition of percentage contribution shares

Percentage shares per single comparison j

For each factor X_i, the logarithmic share is defined, i.e. the percentage contribution of factor *i-th* to the total variation of $Y_{m,o}$:

$$\text{Share}_{X_{i,j}}(\%) = \frac{\Delta\ln(X_i)_j}{\Delta\ln(Y_{m,o})_j} \times 100$$

where:

- j = index of the experimental comparison;
- $\Delta\ln(X_i)_j$ = logarithmic variation of X_i (leaf or root yield of VDL or CAT) in comparison *j*
- $\Delta\ln(Y_{m,o})_j$ = variazione logaritmica di $Y_{m,o}$ (leaf or root yield of VDL or CAT) nel confronto *j*

Interpretation:

- Se Share_{Xi} >0 , the factor contributes positively to the increase in $Y_{m,o}$;
- Se Share_{Xi} <0, the factor tends to reduce $Y_{m,o}$;

- The sum of all factor shares equals 100% (apart from numerical rounding effects)

Weighted mean (WM) percentage shares

Individual factor shares (Share_{Xi}) express the percentage contribution (%) of each factor to the total variation of yield ($Y_{m,o}$) in a single specific comparison. Therefore, for experimental factors with only two levels (*e.g.*, PAW treatment: YES/NO) and for single pairwise sampling time comparisons (*e.g.*, T2 vs T1 or T2 vs T0), averaging shares is not meaningful, and individual shares are sufficient.

However, for light spectra, for comparisons between T2 and the other sampling time levels (T1 and T0) as well as for comparisons across PAW:sampling time combinations, it is necessary to summarise all comparisons sharing the same reference condition. Therefore, a weighted mean of shares (weighted mean share) was calculated to represent the overall average contribution of each factor to the increase (or decrease) in yield under the reference condition.

The weighted mean (WM) is calculated as:

$$\text{Share}_{X_{i,WM}} = \frac{\sum_{j=1}^{ncomp} \left|\Delta ln(Y_{m,o})_j\right| \cdot \text{Share}_{X_{i,j}}}{\sum_{j=1}^{ncomp} \left|\Delta ln(Y_{m,o})_j\right|}$$

where:

- j = index of the experimental comparison;
- $n_{con\ mp}$ = total number of comparisons considered (*e.g.*, n=6 for LED light spectrum: RB vs all the other spectra; n=2 for sampling time considering T2 vs T1 and T0, n=3 for PAW:sampling time considering YES:T2 vs NO:T2, YES:T1, and NO:T1)
- $\text{Share}_{Xi,j}$ = share (non-percentage form) of factor X_i (leaf/root DM and concentration of VDL or CAT) in comparison *j*
- $\Delta ln(Y_{m,o})_j$= logarithmic variation of $Y_{m,o}$ (leaf or root yield of VDL or CAT) nel confronto j;
- $\left|\Delta ln(Y_{m,o})_j\right|$= absolute value of the logarithmic variation of $Y_{m,o}$ in comparison *j*, used as a weight to avoid cancellation between positive and negative variations and proportional to the magnitude of the total variation

The absolute value of $\Delta ln(Y_{m,o})_j$ is used only to define weights, ensuring that comparisons with larger yield variations have greater influence on the weighted mean and preventing numerical cancellation between positive and negative variations. The factor shares themselves are not transformed and therefore retain their original sign, preserving the direction (increase or decrease) of each factor's average contribution. Since logarithmic decomposition expresses contributions in logarithmic units, factor shares may exceed 100% or be negative when some factors reinforce the overall variation while others counteract it.

Weighted mean shares thus allow identification of which physiological factors (*e.g.*, dry mass or concentration) under the reference condition contributed on average most strongly to the variation in yield under the same reference condition. Positive values indicate factors that promoted yield increase, whereas negative values indicate factors that opposed this variation.

For example:

- If the weighted mean share of leaf dry mass (DM_{leaf}) is higher than that of leaf concentration (C_{leaf}) in RB vs other spectra comparisons, this indicates that the increase in leaf yield under RB is mainly due to a greater increase in biomass rather than concentration under the same spectral condition (the same reasoning applies to the other comparisons).
- Conversely, if the mean weighted share of concentration is higher, it means that the higher yield under RB is mainly due to metabolic enrichment under the same spectral condition.

In this way, weighted mean shares provide a synthetic but physiologically interpretable measure of the average percentage contribution of factors explaining the increase (or decrease) in yield under the condition of interest.

Application to the specific case of this study

For VDL and CAT:

$$Y_{leaf} = DM_{leaf} \cdot C_{leaf}$$

$$Y_{root} = DM_{root} \cdot C_{root}$$

where:

- $Y_{leaf =}$ leaf VDL/CAT yield
- DM_{leaf} = leaf dry mass
- C_{leaf} = leaf VDL/CAT concentration

- Y_{root} = root VDL/CAT yield
- DM_{root} = root dry mass
- C_{root} = root VDL/CAT concentration

Logarithmic variations for a single comparison between ref and cond conditions

$$\Delta\ln(Y_{leaf,j}) = \ln(Y_{leaf,ref}) - \ln(Y_{leaf,cond,j}) = [\ln(DM_{leaf,ref}) - \ln(DM_{leaf,cond,j})] + [\ln(C_{leaf,ref}) - \ln(C_{leaf,cond,j})]$$

$$\Delta\ln(Y_{root,j}) = \ln(Y_{root,ref}) - \ln(Y_{root,cond,j}) = [\ln(DM_{root,ref}) - \ln(DM_{root,cond,j})] + [\ln(C_{root,ref}) - \ln(C_{root,cond,j})]$$

$$\Delta\ln(Y_{leaf,j}) = \Delta\ln(DM_{leaf,j}) + \Delta\ln\,(C_{leaf,j})$$
$$\Delta\ln(Y_{root,j}) = \Delta\ln(DM_{root,j}) + \Delta\ln\,(C_{root,j})$$

Weighted logarithmic variation for VDL or CAT in both leaves and roots:

$$\Delta\ln(DM_{leaf})_{WM} = \frac{\sum_{j=1}^{ncomp}\left|\Delta ln(Y_{leaf})_j\right| \cdot \Delta ln(DM_{leaf})_j}{\sum_{j=1}^{ncomp}\left|\Delta ln(Y_{leaf})_j\right|}$$

$$\Delta\ln(C_{leaf})_{WM} = \frac{\sum_{j=1}^{ncomp}\left|\Delta ln(Y_{leaf})_j\right| \cdot \Delta ln(C_{leaf})_j}{\sum_{j=1}^{ncomp}\left|\Delta ln(Y_{leaf})_j\right|}$$

$$\Delta\ln(Y_{leaf})_{WM} = \frac{\sum_{j=1}^{ncomp}\left|\Delta ln(Y_{leaf})_j\right| \cdot \Delta ln(Y_{leaf})_j}{\sum_{j=1}^{ncomp}\left|\Delta ln(Y_{leaf})_j\right|}$$

$$\Delta\ln(DM_{root})_{WM} = \frac{\sum_{j=1}^{ncomp}\left|\Delta ln(Y_{root})_j\right| \cdot \Delta ln(DM_{root})_j}{\sum_{j=1}^{ncomp}\left|\Delta ln(Y_{root})_j\right|}$$

$$\Delta\ln(C_{root})_{WM} = \frac{\sum_{j=1}^{ncomp}\left|\Delta ln(Y_{root})_j\right| \cdot \Delta ln(C_{root})_j}{\sum_{j=1}^{ncomp}\left|\Delta ln(Y_{root})_j\right|}$$

$$\Delta\ln(Y_{root})_{WM} = \frac{\sum_{j=1}^{ncomp}\left|\Delta ln(Y_{root})_j\right| \cdot \Delta ln(Y_{root})_j}{\sum_{j=1}^{ncomp}\left|\Delta ln(Y_{root})_j\right|}$$

Percentage shares per organ and single comparison:

$$\text{Share}_{DM_{leaf},j}(\%) = \frac{\Delta ln(DM_{leaf})j}{\Delta\ln\,(Y_{leaf})j} \times 100$$

$$\text{Share}_{DM_{root},j}(\%) = \frac{\Delta ln(DM_{root})j}{\Delta\ln\,(Y_{root})j} \times 100$$

$$\text{Share}_{C_{leaf},j}(\%) = \frac{\Delta\ln\,(C_{leaf}j}{\Delta\ln\,(Y_{leaf})j} \times 100$$

$$\text{Share}_{C_{root},j}(\%) = \frac{\Delta\ln\,(C_{root})j}{\Delta\ln\,(Y_{root})j} \times 100$$

Weighted mean (WM) percentage shares that summarize all comparisons under a single reference condition:

$$\text{Share}_{DM_{leaf},WM}(\%) = \frac{\sum_{j=1}^{ncomp}\left|\Delta\ln(Y_{leaf})_j\right| \cdot \text{Share}_{DM_{leaf},j}}{\sum_{j=1}^{ncomp}\left|\Delta\ln(Y_{leaf})_j\right|}$$

$$\text{Share}_{DM_{root},WM}(\%) = \frac{\sum_{j=1}^{ncomp}\left|\Delta\ln(Y_{root})_j\right| \cdot \text{Share}_{DM_{root},j}}{\sum_{j=1}^{ncomp}\left|\Delta\ln(Y_{root})_j\right|}$$

$$\text{Share}_{C_{leaf},WM}(\%) = \frac{\sum_{j=1}^{ncomp}\left|\Delta\ln(Y_{leaf})_j\right| \cdot \text{Share}_{C_{leaf},j}}{\sum_{j=1}^{ncomp}\left|\Delta\ln(Y_{leaf})_j\right|}$$

$$\text{Share}_{C_{root},WM}(\%) = \frac{\sum_{j=1}^{ncomp}\left|\Delta\ln(Y_{root})_j\right| \cdot \text{Share}_{C_{root},j}}{\sum_{j=1}^{ncomp}\left|\Delta\ln(Y_{root})_j\right|}$$

2.5 LMDI additive

In the case of additive variables, the total variation can no longer be separated through a simple logarithmic sum. In this scenario, the additive form of LMDI is used, which employs logarithmic means as weights to correctly distribute the total variation among the different components. This approach ensures an exact, symmetric decomposition with no residual, allowing the contribution of each compartment or organ (leaf and root) to the observed total variation to be assessed.

The LMDI method was applied according to two approaches corresponding to the two previously described methods for calculating total yields:

- *First approach (structural or organ-specific approach): organ decomposition (Leaf vs Root).* The logarithmic variation of total dry mass or total yield is expressed as the sum of contributions from the two main organs treated as independent additive components.
- **S***econd approach (physiological approach): physiological decomposition (DM and C).* The variation in yield is decomposed into contributions arising from the two main physiological factors (dry mass and concentration) within each organ.

2.5.1 General definition of the LMDI first approach (structural or organ-specific – total dry mass)

$$\Delta ln(DM_{tot)} = \sum_{i=1}^{n} \omega_i \cdot \Delta ln(DM_i)$$

where:

- $\Delta \ln(DM_{tot}) = \ln(DM_{tot,ref}) - \ln(DM_{tot,cond})$ = total logarithmic variation of total dry mass between the reference condition (*ref*) and the comparison condition (*cond*)
- $\Delta \ln(DM_i) = \ln(DM_{i,ref}) - \ln(DM_{i,cond})$ = logarithmic variation of dry mass in organ *i* (leaf or root) between *ref* and *cond* conditions
- $\omega_{i,m}$ = MDI weight associated with organ *i*, reflecting its relative importance in the total variation, calculated as:

$$\omega_{i,m} = \frac{L(DM_{i,ref}, DM_{i,cond})}{L(DM_{tot,ref}, DM_{tot,cond})}$$

with L (a, b) is the logarithmic mean of *ref* (a) and *cond* (b) values:

$$L(a,b)\begin{cases} \frac{a-b}{\ln(a)-\ln(b)} & a\neq b \\ a & a=b \end{cases} \quad a > 0, b > 0$$

where:

- a e b are positive values (> 0)
- Numerator:

  a= $DM_{i,\ ref}$: dry mass of organ *i* (leaf or root) under the *ref* condition

  b=$DM_{i,,cond}$: dry mass of organ *i* (leaf or root) under *cond* condition

- Denominator:

  a=$DM_{tot,ref}$: total dry mass under the *ref* condition

  b=$DM_{tot,cond}$: total dry mass under the *cond* condition

Logarithmic variation per single comparison j

$$\Delta \ln(DM_{i,j}) = \ln(DM_{i,ref}) - \ln(DM_{i,cond,j})$$

$$\Delta ln(DM_{tot)} = \ln(DM_{tot,ref}) - \ln(DM_{tot,cond,j})$$

Weighted mean logarithmic variations

For each variable DM_i, the weighted mean (WM) of the logarithmic variation was calculated as:

$$\Delta\ln(DM_i)_{WM} = \frac{\sum_{j=1}^{ncomp}\left|\Delta ln(DM_{tot})_j\right| \cdot \Delta ln(DM_i)_j}{\sum_{j=1}^{ncomp}\left|\Delta ln(DM_{tot})_j\right|}$$

where:

- j = index of the individual experimental comparison
- n_{comp} = total number of comparisons considered (*e.g.*, n=6 for LED light spectrum: RB vs all the other spectra; n=2 for sampling time considering T2 vs T1 and T0, n=3 for PAW:sampling time considering YES:T2 vs NO:T2, YES:T1, and NO:T1)
- $\Delta ln(DM_i)_j$ = logarithmic variation of variable DW_i for the individual comparison j
- $\left|\Delta ln(DM_{tot})_j\right|$ = weight assigned to each comparison, given by the absolute value of the logarithmic variation of DM_{tot} for the individual comparison j

Analogously, the weighted mean total logarithmic variation (WM) of $Y_{m,o}$ was calculated as:

$$\Delta\ln(DM_{tot})_{WM} = \frac{\sum_{j=1}^{ncomp}\left|\Delta ln(DM_{tot})_j\right| \cdot \Delta ln(DM_{tot})_j}{\sum_{j=1}^{ncomp}\left|\Delta ln(DM_{tot})_j\right|}$$

where:

- $\Delta ln(DM_{tot})_j$ = logarithmic variation of the variable DM_{tot} for the individual comparison j

Structural contributions

For each organ *i*, partial contributions are defined as:

$$Contr_{\mathrm{DM}i} = \omega_i \cdot \Delta\ln\,(DM_i)$$

The total variation is exactly decomposed as:

$$\Delta\ln\,(DM_{tot}) = \sum_{i=1}^{n} \mathrm{Contr}_{\mathrm{DM}i}$$

Each contribution can also be expressed as a sum of single organ *i* contributions:

$$\mathrm{Contr}_{DM_{tot}} = \sum_{i=1}^{n} \mathrm{Contr}_{\mathrm{DM}i}$$

Structural contributions per single comparison j

$$Contr_{\mathrm{DM}i,j} = \omega_{i,j} \cdot \Delta\ln(DM_i)_j$$

where

$$\Delta\ln\,(DM_i)_j = \ln\left(DM_{i,ref}\right) - \ln(DM_{i,cond})_j$$

Weighted mean (WM) structural contributions:

$$Contr_{DM_{i,WM}} = \frac{\sum_{j=1}^{ncomp}\left|\Delta\ln\,(DM_{tot})_j\right| \cdot Contr_{DMi,j}}{\sum_{j=1}^{ncomp}\left|\Delta\ln\,(DM_{tot})_j\right|}$$

where:

- j = index of the individual experimental comparison
- n_{comp} = total number of comparisons considered (*e.g.*, n=6 for LED light spectrum: RB vs all the other spectra; n=2 for sampling time considering T2 vs T1 and T0, n=3 for PAW:sampling time considering YES:T2 vs NO:T2, YES:T1, and NO:T1)
- $Contr_{DMi,j}$ = partial contribution for comparison j
- $\left|\Delta\ln\,(DM_{tot})_j\right|$ = weight assigned to each comparison, given by the absolute value of the logarithmic variation of DM_{tot} for the individual comparison j

Weighted mean total logarithmic (WM) variation of DM_{tot}:

$$\Delta\ln(DM_{tot})_{WM} = \frac{\sum_{j=1}^{ncomp}\left|\Delta\ln\,(DM_{tot})_j\right| \cdot \Delta\ln\,(DM_{tot})_j}{\sum_{j=1}^{ncomp}\left|\Delta\ln\,(DM_{tot})_j\right|}$$

where:

- $\Delta\ln\,(DM_{tot})_j$ = logarithmic variation of the variable DM_{tot} for the individual comparison j

Structural percentage shares for single comparisons j

Single shares indicate the percentage of variation attributable to each organ in a single comparison.

$$\mathrm{Share}_{DM_{i,j}}(\%) = \frac{\mathrm{Contr}_{DM_{i,j}}}{\Delta\ln\,(DM_{tot})_j} \times 100$$

where

- j = index of the individual experimental comparison
- $\mathrm{Contr}_{DM_{i,j}}$ = partial contribution for comparison j
- $\Delta\ln\,(DM_{tot})_j$ = logarithmic variation of the variable DM_{tot} for the individual comparison j

Weighted mean (WM) percentage structural shares

Weighted mean shares provide a synthetic and robust estimate of the average contribution of each component across all comparisons under a single reference condition, allowing assessment of whether the total increase in dry mass is mainly driven by a specific organ under the same condition.

$$\text{Share}_{DM_i,MW}(\%) = \frac{\sum_{j=1}^{ncomp} \left|\Delta ln(DM_{tot})_j\right| \cdot \text{Share}_{DM_i,j}}{\sum_{j=1}^{ncomp} \left|\Delta ln(DM_{tot})_j\right|}$$

where:

- $j =$ index of the individual experimental comparison
- n_{comp} = total number of comparisons considered (*e.g.*, n=6 for LED light spectrum: RB vs all the other spectra; n=2 for sampling time considering T2 vs T1 and T0, n=3 for PAW:sampling time considering YES:T2 vs NO:T2, YES:T1, and NO:T1);
- $\text{Share}_{DM_i,j}$= the ratio between $\text{Contr}_{DM_i,j}$ and $\Delta\text{ln}\ (DM_{tot})_j$, for each comparison j, *i.e.* the contribution share of dry mass in organ *i* (leaves or roots) in comparison *j*.
- $\Delta ln(DM_{tot})_j$= logarithmic variation of the variable DM_{tot} for the individual comparison *j*
- $\left|\Delta ln(DM_{tot})_j\right|$= absolute value of the logarithmic change in DM_{tot} in comparison *j*, used as a weighting factor to prevent negative variations from cancelling out positive ones and proportional to the magnitude of the total change.

Application to the present study

Total dry biomass is defined as:

$$DM_{tot} = DM_{leaf} + DM_{root}$$

Logarithmic variations per comparison *j*:

$$\Delta\text{ln}(\text{DM}_{\text{leaf}})_{\text{j}} = \text{ln}(\text{DM}_{\text{leaf,ref}}) - \text{ln}(\text{DM}_{\text{leaf,cond}})_{\text{j}}$$

$$\Delta\text{ln}(\text{DM}_{\text{root}})_{\text{j}} = \text{ln}(\text{DM}_{\text{root,ref}}) - \text{ln}(\text{DM}_{\text{root,cond}})_{\text{j}}$$

$$\Delta\ln(\mathrm{DM_{tot}})_j = \ln(\mathrm{DM_{tot,ref}}) - \ln(\mathrm{DM_{tot,cond}})_j$$

Weighted mean (WM) logarithmic variations:

$$\Delta\ln(DM_{tot})_{WM} = \frac{\sum_{j=1}^{ncomp}\left|\Delta ln(DM_{tot})_j\right| \cdot \Delta ln(DM_{tot})_j}{\sum_{j=1}^{ncomp}\left|\Delta ln(DM_{tot})_j\right|}$$

Organ-specific LMDI weights

$$\omega_{leaf} = \frac{L(DM_{leaf,ref}, DM_{leaf,cond})}{L(DM_{tot,ref}, DM_{tot,cond})}$$

$$\omega_{root} = \frac{L(DM_{root,ref}, DM_{root,cond})}{L(DM_{tot,Ref}, DM_{tot,cond})}$$

Organ-specific contributions per single comparison j:

$$Contr_{DM,leaf,j} = \omega_{leaf,j} \cdot \Delta\ln\,(DM_{leaf})_j$$

$$Contr_{DM,root,j} = \omega_{root,j} \cdot \Delta\ln(DM_{root})_j$$

where

$$\Delta\ln\,(DM_{leaf})j = \ln(DM_{leaf,ref}) - \ln(DM_{leaf,cond})_j$$

$$\Delta\ln\,(DM_{root})_j = \ln(DM_{root,ref}) - \ln(DM_{root,cond})_j$$

and

$$\Delta\ln\,(DM_{tot})_j = Contr_{DM,leaf,j} + Contr_{DM,root,j}$$

Weighted mean (WM) contributions

$$Contr_{DM_{leaf},WM} = \frac{\sum_{j=1}^{ncomp}\left|\Delta\ln\,(DM_{tot})_j\right| \cdot Contr_{DMleaf,j}}{\sum_{j=1}^{ncomp}\left|\Delta\ln\,(DM_{tot})_j\right|}$$

$$Contr_{DM_{root}WM} = \frac{\sum_{j=1}^{ncomp}\left|\Delta\ln\,(DM_{tot})_j\right| \cdot Contr_{DMroot,j}}{\sum_{j=1}^{ncomp}\left|\Delta\ln\,(DM_{tot})_j\right|}$$

$$\Delta\ln(DM_{tot})_{WM} = \frac{\sum_{j=1}^{ncomp}\left|\Delta\ln\,(DM_{tot})_j\right| \cdot \Delta\ln\,(DM_{tot})_j}{\sum_{j=1}^{ncomp}\left|\Delta\ln\,(DM_{tot})_j\right|}$$

Structural percentage shares for single comparison j:

$$\mathrm{Share}_{DM_{leaf,j}}(\%) = \frac{Contr_{DM_{leaf,j}}}{\Delta\ln\,(DM_{tot})_j} \times 100$$

$$\text{Share}_{DM_{root}}(\%) = \frac{Contr_{DM_{root}}}{\Delta\ln\,(DM_{tot})_j} \times 100$$

Weighted mean (WM) percentage structural shares:

$$\text{Share}_{DM_{leaf},WM}(\%) = \frac{\sum_{j=1}^{ncomp} \left|\Delta ln(DM_{tot})_j\right| \cdot \text{Share}_{DM_{leaf},j}}{\sum_{j=1}^{ncomp} \left|\Delta ln(DM_{tot})_j\right|}$$

$$\text{Share}_{DM_{root},WM}(\%) = \frac{\sum_{j=1}^{ncomp} \left|\Delta ln(DM_{tot})_j\right| \cdot \text{Share}_{DM_{root},j}}{\sum_{j=1}^{ncomp} \left|\Delta ln(DM_{tot})_j\right|}$$

2.5.2 General definition of the LMDI first approach (structural or organ-specific – total alkaloid yield)

$$\Delta ln(Y_{tot,m}) = \sum_{i=1}^{n} \omega_{i,m} \cdot \Delta ln(Y_{i,m})$$

where:

- $\Delta\ln(Y_{tot,m}) = \ln(Y_{tot,m,ref}) - \ln\,(Y_{tot,m,cond})$ = total logarithmic variation of the total yield of metabolite *m* (VDL or CAT) between the reference (*ref)* and comparison (*cond)* conditions
- $\Delta\ln(Y_{i,m}) = \ln(Y_{i,m,ref}) - \ln\,(Y_{i,m,cond})$ = logarithmic variation of the yield of metabolite *m* in organ *i* (leaves or roots) between the *ref* and *cond* conditions
- $\omega_{i,m}$ = LMDI weight associated with metabolite *m* in organ *i*, reflecting its relative importance in the total variation, calculated as:

$$\omega_{i,m} = \frac{L(Y_{i,m,ref}, Y_{i,m,cond})}{L(Y_{tot,m,ref}, Y_{tot,m,cond})} \text{ with}$$

with L (a, b) =is the logarithmic mean of *ref* (a) and *cond* (b) values:

$$L(a,b) \begin{cases} \frac{a-b}{\ln(a)-\ln(b)} & a \neq b \\ a & a = b \end{cases} \quad a > 0,\ b > 0$$

where:

- a, b are positive values (> 0)
- *Numerator*:

a= $Y_{i,m,ref}$: yield of metabolite m (VDL or CAT) in organ *i* (leaf or root) under the *ref* condition;

b=$Y_{i,,m,\ cond}$: yield of metabolite m (VDL or CAT) in organ *i* (leaf or root) under the *cond* condition

– <u>*Denominator*</u>:

a= $Y_{tot,m\ ref}$: total yield of metabolite *m* under the *ref* condition;

b=$Y_{tot,m,cond}$: total yield of metabolite *m* under the *cond* condition;

Logarithmic variation per single comparison

$$\Delta\ln(Y_{i,m})_j = \ln(Y_{i,m,ref}) - \ln(Y_{i,m,cond})_j$$

$$\Delta\ln(Y_{tot,m})_j = \ln(Y_{tot,m,ref})_j - \ln(Y_{tot,m,cond})_j$$

Weighted mean (WM) logarithmic variations

$$\Delta\ln(Y_{i,m})_{WM} = \frac{\sum_{j=1}^{ncomp} |\Delta ln(Y_{tot,m})_j| \cdot \Delta ln(Y_{i,m})_j}{\sum_{j=1}^{ncomp} |\Delta ln(Y_{tot,m})_j|}$$

where:

- j = index of the individual experimental comparison
- n_{comp} = total number of comparisons considered (*e.g.*, n=6 for LED light spectrum: RB vs all the other spectra; n=2 for sampling time considering T2 vs T1 and T0, n=3 for PAW:sampling time considering YES:T2 vs NO:T2, YES:T1, and NO:T1);
- $\Delta ln(Y_{i,m})_j$ = logarithmic variation of variable $Y_{i,m}$ for the individual comparison j
- $|\Delta ln(Y_{tot,m})_j|$ = absolute value of the logarithmic change in the variable $Y_{tot,m}$ for comparison j, used as a weight to prevent negative variations from cancelling out positive ones and proportional to the magnitude of the total change.

Similarly, the total weighted mean (WM) log variation of $Y_{tot,m}$ was calculated:

$$\Delta\ln(Y_{tot,m})_{WM} = \frac{\sum_{j=1}^{ncomp} |\Delta ln(Y_{tot,m})_j| \cdot \Delta ln(Y_{tot,m})_j}{\sum_{j=1}^{ncomp} |\Delta ln(Y_{tot,m})_j|}$$

where:

- $\Delta ln(Y_{tot,m})_j$ = logarithmic variation of the variable $Y_{tot,m}$ for the individual comparison j

Structural contributions

For each organ *i* and metabolite *m*, partial contributions are defined as:

$$Contr_{Yi,m} = \omega_{i,m} \cdot \Delta\ln(Y_{i,m})$$

The total variation is exactly decomposed as::

$$\Delta \ln\,(Y_{tot,m}) = \sum_{i=1}^{n} \mathrm{Contr}_{\mathrm{Y}i,m}$$

Each contribution can also be expressed as the sum of physiological contributions

$$\mathrm{Contr}_{\mathrm{Y}i,m} = \mathrm{Contr}_{\mathrm{DM},i} + \mathrm{Contr}_{\mathrm{C},i,m}$$

Structural contributions for single comparison j

$$Contr_{Yi,m,j} = \omega_{i,m,j} \cdot \Delta \ln\,(Y_{i,m}) j$$

where

$$\Delta \ln\,(Y_{i,m})_j = \ln\left(Y_{i,m,ref}\right) - \ln(Y_{i,m,cond})_j$$

In total:

$$\Delta \ln\,(Y_{tot,m})_j = \sum_{i=1}^{n} \mathrm{Contr}_{\mathrm{Y}i,m,j}$$

Weighted mean (WM) structural contribution

$$Contr_{Y_{i,m}WM} = \frac{\sum_{j=1}^{ncomp} \left|\Delta \ln\,(Y_{tot,m})_j\right| \cdot Contr_{Yi,m,j}}{\sum_{j=1}^{ncomp} \left|\Delta \ln\,(Y_{tot,m})_j\right|}$$

where:

- *j* = index of the individual experimental comparison
- n_{comp} = total number of comparisons considered (*e.g.*, n=6 for LED light spectrum: RB vs all the other spectra; n=2 for sampling time considering T2 vs T1 and T0, n=3 for PAW:sampling time considering YES:T2 vs NO:T2, YES:T1, and NO:T1);
- $Contr_{Yi,m,j}$ = partial contribution for comparison *j*
- $\left|\Delta \ln\,(Y_{tot,m})_j\right|$ = absolute value of the logarithmic change in the variable $Y_{tot,m}$ for comparison *j*, used as a weight to prevent negative variations from cancelling out positive ones and proportional to the magnitude of the total change.

Weighted mean total logarithmic variation of $Y_{tot,m}$*:*

$$\Delta \ln\left(Y_{tot,m}\right)_{WM} = \frac{\sum_{j=1}^{ncomp} \left|\Delta \ln\,(Y_{tot,m})_j\right| \cdot \Delta \ln\,(Y_{tot,m})_j}{\sum_{j=1}^{ncomp} \left|\Delta \ln\,(Y_{tot,m})_j\right|}$$

where:

- $\Delta\ln\,(Y_{tot,m})_j$ = logarithmic variation of the variable $Y_{tot,m}$ for the individual comparison *j*

Structural percentage shares for single comparison j

Individual shares represent the percentage contribution of variables $Y_{i,m}$ to variable $Y_{tot,m}$

$$\mathrm{Share}_{Y_{i,m,j}}(\%) = \frac{\mathrm{Contr}_{Y_{i,},m,j}}{\Delta\ln\,(Y_{tot,m})_j} \times 100$$

where

- *j* = index of the individual experimental comparison
- $\mathrm{Contr}_{Y_{i,},m,j}$ = partial contribution for single comparison *j*
- $\Delta\ln\,(Y_{tot,m})_j$ = logarithmic variation of the variable $Y_{tot,m}$ for the single comparison *j*

Weighted mean (WM) structural percentage shares

These provide a synthetic and robust estimate of the average contribution of different components across all comparisons under a single reference condition, allowing assessment of whether the increase in total alkaloid yield under a given condition is mainly driven by a specific organ.

$$\mathrm{Share}_{Y_{i,m}MW} = \frac{\sum_{j=1}^{ncomp}\left|\Delta ln(Y_{tot,m})_j\right| \cdot \mathrm{Share}_{Y_{i,m},j}}{\sum_{j=1}^{ncomp}\left|\Delta ln(Y_{tot,m})_j\right|}$$

where:

- j = index of the individual experimental comparison;
- n_{comp} = total number of comparisons considered;
- $\mathrm{Share}_{Y_{i,m},j}$= ratio between $\mathrm{Contr}_{Y_{i,},m,j}$ e $\Delta\ln\,(Y_{tot,m})_j$ for each comparison j, *i.e.*, the share of dry mass contribution of organ *i* (leaves or roots) in comparison j.;
- $\Delta ln(Y_{tot,m})_j$= variazione logaritmica di $Y_{tot,m}$ nel confronto j;
- $\left|\Delta ln(Y_{tot,m})_j\right|$= absolute value of the logarithmic change in the variable $Y_{tot,m}$ for comparison *j*, used as a weight to prevent negative variations from cancelling out positive ones and proportional to the magnitude of the total change.

Application to the present study

For VDL and CAT:

$$Y_{tot} = Y_{leaf} + Y_{root}$$

$$(Y_{leaf} = DM_{leaf} \cdot C_{leaf})$$

$$(Y_{root} = DM_{root} \cdot C_{root})$$

Logarithmic variations per comparison j:

$$\Delta\ln(Y_{leaf})_j = \ln\left(Y_{leaf,ref}\right) - \ln\,(Y_{leaf,cond})_j$$

$$\Delta\ln(Y_{root})_j = \ln\left(Y_{root,ref}\right) - \ln\,(Y_{root,cond})_j$$

$$\Delta\ln\,(Y_{tot})_j = \ln\left(Y_{tot,ref}\right) - \ln\,(Y_{tot,cond})_j$$

Weighted mean (WM) logarithmic variations:

$$\Delta\ln\left(Y_{leaf}\right)_{WM} = \frac{\sum_{j=1}^{ncomp}\left|\Delta ln(Y_{tot})_j\right| \cdot \Delta ln(Y_{leaf})_j}{\sum_{j=1}^{ncomp}\left|\Delta ln(Y_{tot})_j\right|}$$

$$\Delta\ln(Y_{root})_{WM} = \frac{\sum_{j=1}^{ncomp}\left|\Delta ln(Y_{tot})_j\right| \cdot \Delta ln(Y_{root})_j}{\sum_{j=1}^{ncomp}\left|\Delta ln(Y_{tot})_j\right|}$$

$$\Delta\ln(Y_{tot})_{WM} = \frac{\sum_{j=1}^{ncomp}\left|\Delta ln(Y_{tot})_j\right| \cdot \Delta ln(Y_{tot})_j}{\sum_{j=1}^{ncomp}\left|\Delta ln(Y_{tot})_j\right|}$$

Organ-specific LMDI weights

$$\omega_{leaf} = \frac{L(Y_{leaf,ref}, Y_{leaf,cond})}{L(Y_{tot,ref}, Y_{tot,cond})}$$

$$\omega_{root} = \frac{L(Y_{root,ref}, Y_{root,cond})}{L(Y_{tot,Ref}, Y_{tot,cond})}$$

Organ-specific contributions per single comparison j:

$$Contr_{Y_{leaf,j}} = \omega_{leaf} \cdot \Delta\ln(Y_{leaf})_j$$

$$Contr_{Y_{root,j}} = \omega_{root} \cdot \Delta\ln(Y_{root})_j$$

where

$$\Delta\ln(Y_{leaf})_j = \ln\left(Y_{leaf,ref}\right) - \ln(Y_{leaf,cond})_j$$

$$\Delta\ln(Y_{root})_j = \ln\left(Y_{root,ref}\right) - \ln(Y_{root,cond})_j$$

$$\Delta\ln(Y_{tot}) = Contr_{Y_{leaf}} + Contr_{Y_{root}}$$

Relation with the second approach:

$$Contr_{Y_{leaf}} = Contr_{DM_{leaf}} + Contr_{C_{leaf}}$$

$$Contr_{Y_{root}} = Contr_{DM_{root}} + Contr_{C_{root}}$$

Weighted mean (WM) contributions:

$$Contr_{Y_{leaf},WM} = \frac{\sum_{j=1}^{ncomp} \left|\Delta\ln\,(Y_{tot})_j\right| \cdot \,Contr_{Yleaf,j}}{\sum_{j=1}^{ncomp} \left|\Delta\ln\,(Y_{tot})_j\right|}$$

$$Contr_{Y_{root}WM} = \frac{\sum_{j=1}^{ncomp} \left|\Delta\ln\,(Y_{tot})_j\right| \cdot \,Contr_{Yroot,j}}{\sum_{j=1}^{ncomp} \left|\Delta\ln\,(Y_{tot})_j\right|}$$

$$\Delta\ln(Y_{tot})_{WM} = \frac{\sum_{j=1}^{ncomp} \left|\Delta\ln\,(Y_{tot})_j\right| \cdot \Delta\ln\,(Y_{tot})_j}{\sum_{j=1}^{ncomp} \left|\Delta\ln\,(Y_{tot})_j\right|}$$

Percentage shares per single comparison j:

$$\text{Share}_{Y_{leaf},j}(\%) = \frac{Contr_{Y_{leaf,j}}}{\Delta ln(Y_{tot})_j} \times 100$$

$$\text{Share}_{Y_{root},j}(\%) = \frac{Contr_{Y_{root,j}}}{\Delta ln(Y_{tot})_j} \times 100$$

Weighted mean (WM) structural percentage shares:

$$\text{Share}_{Y_{leaf},WM}(\%) = \frac{\sum_{j=1}^{ncomp} \left|\Delta ln(Y_{tot})_j\right| \cdot \text{Share}_{Y_{leaf},j}}{\sum_{j=1}^{ncomp} \left|\Delta ln(Y_{tot})_j\right|}$$

$$\text{Share}_{Y_{root},WM}(\%) = \frac{\sum_{j=1}^{ncomp} \left|\Delta ln(Y_{tot})_j\right| \cdot \text{Share}_{Y_{root},j}}{\sum_{j=1}^{ncomp} \left|\Delta ln(Y_{tot})_j\right|}$$

2.5.3 General definition of the second LMDI approach (physiological decomposition):

The logarithmic variation of the total yield is obtained as::

$$\Delta ln(Y_{tot,m)} = \sum_{i=1}^{n} \omega_{i,m} \cdot \Delta ln(Y_{i,m})$$

and, by substitution:

$$ln(Y_{tot,m}) = \sum_{i=1}^{n} \omega_{i,m}\, \Delta ln(DM_i) + \omega_{i,m} \Delta ln(C_{i,m})$$

where:

- $\Delta\ln(Y_{tot,m}) = \ln(Y_{tot,m,ref}) - \ln(Y_{tot,m,cond})$ = total logarithmic variation of the total yield of metabolite *m* (VDL or CAT) between the reference (*ref*) and comparison (*cond*) conditions
- $\Delta\ln(Y_{i,m}) = [\ln(DM_{i,ref}) - \ln(DM_{i,cond})] + [\ln(C_{i,m,ref}) - \ln(C_{i,m,cond})] = \Delta\ln(DM_{i,}) + \Delta\ln(C_{i,m,})$ = logarithmic variation of *m* yield (VDL or CAT) in organ *i* (leaves or roots) between the *ref* and *cond* conditions
- $\omega_{i,m}$ = LMDI weight associated with metabolite *m* (VDL or CAT) in organ *i* (leaves or roots), reflecting its relative importance in the total variation, defined as:

$$\omega_{i,m} = \frac{L(Y_{i,m,ref}, Y_{i,m,cond})}{L\,(Y_{tot,m,ref}, Y_{tot,m,cond})}$$

with L (a, b) = logarithmic mean of *ref* (a) and *cond* (b) values:

$$L(a,b)\begin{cases} \frac{a-b}{\ln(a)-\ln(b)} & a\neq b \\ a & a=b \end{cases} \quad a>0, b>0$$

where:

- a, b are positive values (> 0)
- *Numerator*:

  a= $Y_{i,m,ref}$ = yield of metabolite *m* (VDL or CAT) in organ *i* under the *ref* condition;

  b=$Y_{i,,m,cond}$= yield of metabolite m in organ i (leaf or root) under *cond* condition

- *Denominator*:

  a= $Y_{tot,m\ ref}$ = total yield of metabolite *m* (VDL o CAT) under the *ref* condition;

  b=$Y_{tot,m,cond}$= total yield of metabolite *m* under the *cond* condition

Logarithmic variations for single comparison j:

$$\Delta \ln(DM_i)_j = \ln(DM_{i,ref}) - \ln\,(DM_{i,cond})_j$$

$$\Delta \ln(C_{i,m})_j = \ln(C_{i,m,ref}) - \ln\,(C_{i,m,cond})_j$$

$$\Delta \ln\,(Y_{tot,m})_j = \ln(Y_{tot,m,ref}) - \ln\,(Y_{tot,m,cond})_j$$

Weighted mean (WM) logarithmic variations:

$$\Delta \ln(DM_i)_{WM} = \frac{\sum_{j=1}^{ncomp} \left|\Delta ln(Y_{tot,m})_j\right| \cdot \Delta ln(DM_i)_j}{\sum_{j=1}^{ncomp} \left|\Delta ln(Y_{tot,m})_j\right|}$$

$$\Delta \ln(C_{i,m})_{WM} = \frac{\sum_{j=1}^{ncomp} \left|\Delta ln(Y_{tot,m})_j\right| \cdot \Delta ln(C_{i,m})_j}{\sum_{j=1}^{ncomp} \left|\Delta ln(Y_{tot,m})_j\right|}$$

where:

- j = index of the individual experimental comparison
- n_{comp} = total number of comparisons considered (*e.g.*, n=6 for LED light spectrum: RB vs all the other spectra; n=2 for sampling time considering T2 vs T1 and T0, n=3 for PAW:sampling time considering YES:T2 vs NO:T2, YES:T1, and NO:T1)
- $\Delta ln(DM_i)_j$ and $\Delta ln(C_{i,m})_j$ = logarithmic variation of variable DM_i and $C_{i,m}$ for the individual comparison
- $\left|\Delta ln(Y_{tot,m})_j\right|$ = absolute value of the logarithmic change in the variable $Y_{tot,m}$ for comparison j, used as a weight to prevent negative variations from cancelling out positive ones and proportional to the magnitude of the total change

Similarly, the total weighted mean (WM) log change of $Y_{tot,m}$ wa salso calculated:

$$\Delta \ln(Y_{tot,m})_{WM} = \frac{\sum_{j=1}^{ncomp} \left|\Delta ln(Y_{tot,m})_j\right| \cdot \Delta ln(Y_{tot,m})_j}{\sum_{j=1}^{ncomp} \left|\Delta ln(Y_{tot,m})_j\right|}$$

where:

- $\Delta ln(Y_{tot,m})_j$ = logarithmic variation of the variable $Y_{tot,m}$ for the individual comparison j

Physiological contributions

$$Contr_{DM_i} = \omega_{i,m} \cdot \Delta\ln\,(DM_i)$$

$$Contr_{C_{i,m}} = \omega_{i,m} \cdot \Delta\ln\,(C_{i,m})$$

The total variation is exactly decomposed as:

$$\Delta\ln\left(Y_{tot,m}\right) = \sum_{i=1}^{n}[Contr_{DM_i} + \,Contr_{C_{i,m}}]$$

and

$$\mathrm{Contr}_{\mathrm{Y}tot,m} = \sum_{i=1}^{n}[Contr_{DM_i} + \,Contr_{C_{i,m}}]$$

Each contribution can in turn be expressed as the sum of the physiological contributions:

$$\mathrm{Contr}_{\mathrm{Y}i,m} = \mathrm{Contr}_{DM_i} + \,\mathrm{Contr}_{C_{i,m}}$$

Physiological contributions for single comparison j:

$$Contr_{DM,i,j} = \omega_{i,m,j} \cdot \Delta\ln\,(DM_i)j$$

$$Contr_{C,i,m,j} = \omega_{i,m,j} \cdot \Delta\ln\,(C_{i,m})j$$

where:

$$\Delta\ln\,(DM_i)_j = \,\ln\left(DM_{i,ref}\right) - \,\ln(DM_{i,cond})_j$$

$$\Delta\ln\,(C_{i,m})_j = \,\ln\left(C_{i,m,ref}\right) - \,\ln(C_{i,m,cond})_j$$

In total:

$$\Delta\ln\,(Y_{tot,m})_j = \sum_{i=1}^{n}[Contr_{DM_{i,j}} + \,Contr_{C_{i,m,j}}]$$

Weighted mean (WM) physiological contributions

$$Contr_{DM_{i,}WM} = \frac{\sum_{j=1}^{ncomp}\left|\Delta\ln\,(Y_{tot,m})_j\right| \cdot \,Contr_{DM,i,j}}{\sum_{j=1}^{ncomp}\left|\Delta\ln\,(Y_{tot,m})_j\right|}$$

$$Contr_{C_{i,m}WM} = \frac{\sum_{j=1}^{ncomp}\left|\Delta\ln\,(Y_{tot,m})_j\right| \cdot \,Contr_{Ci,m,j}}{\sum_{j=1}^{ncomp}\left|\Delta\ln\,(Y_{tot,m})_j\right|}$$

where:

- *j* = index of the individual experimental comparison
- n_{comp} = total number of comparisons considered (*e.g.*, n=6 for LED light spectrum: RB vs all the other spectra; n=2 for sampling time considering T2 vs T1 and T0, n=3 for PAW:sampling time considering YES:T2 vs NO:T2, YES:T1, and NO:T1)
- $Contr_{DM_{i,}}$ e $Contr_{C_{i,m}}$ = partial contributions for the individual comparison *j*
- $|\Delta\ln\,(Y_{tot,m})_j|$ = absolute value of the logarithmic change in the variable $Y_{tot,m}$ for comparison *j*, used as a weight to prevent negative variations from cancelling out positive ones and proportional to the magnitude of the total change

Weighted mean total logarithmic variation (WM) of $Y_{m,o}$:

$$\Delta\ln(Y_{tot,m})_{WM} = \frac{\sum_{j=1}^{ncomp}|\Delta\ln\,(Y_{tot,m})_j| \cdot \Delta\ln\,(Y_{tot,m})_j}{\sum_{j=1}^{ncomp}|\Delta\ln\,(Y_{tot,m})_j|}$$

where:

- $\Delta\ln\,(Y_{tot,m})_j$ = logarithmic variation of the variable $Y_{tot,m}$ for the individual comparison *j*

Physiological percentage shares per single comparison j:

$$\text{Share}_{DM_{i},j}(\%) = \frac{\text{Contr}_{DM_{i},j}}{\Delta\ln\,(Y_{tot,m})_j} \times 100$$

$$\text{Share}_{C_{i,m,j}}(\%) = \frac{\text{Contr}_{C_{i,m,j}}}{\Delta\ln\,(Y_{tot,m})_j} \times 100$$

where:

- j = index of the individual experimental comparison;
- $\text{Contr}_{DM_{i},j}$ e $\text{Contr}_{C_{i,m,j}}$ = partial contributions of DM and C of metabolite *m* (VDL or CAT) in organ i for single comparison *j*
- $\Delta ln(Y_{tot,m})_j$ = ogarithmic variation of$Y_{tot,m}$ in comparison *j*

Weighted mean (WM) physiological percentage shares

They provide a synthetic and robust estimate of the average contribution of the different components across all comparisons analyzed under a single reference condition, allowing to establish whether the overall increase in total alkaloid yield under a given reference condition is driven predominantly by dry mass or metabolite concentration in a given organ under the same reference condition.

$$\text{Share}_{DM_i,WM}(\%) = \frac{\sum_{j=1}^{ncomp} \left|\Delta ln(Y_{tot,m})_j\right| \cdot \text{Share}_{DM_i,j}}{\sum_{j=1}^{nconf} \left|\Delta ln(Y_{tot,m})_j\right|}$$

$$\text{Share}_{C_{i,m},WM}(\%) = \frac{\sum_{j=1}^{ncomp} \left|\Delta ln(Y_{tot,m})_j\right| \cdot \text{Share}_{C_{i,m},j}}{\sum_{j=1}^{ncomp} \left|\Delta ln(Y_{tot,m})_j\right|}$$

where:

- j = index of the individual experimental comparison;
- n_{comp} = total number of comparisons considered (*e.g.*, n=6 for LED light spectrum: RB vs all the other spectra; n=2 for sampling time considering T2 vs T1 and T0, n=3 for PAW:sampling time considering YES:T2 vs NO:T2, YES:T1, and NO:T1)
- $\text{Share}_{DM_i,j}$ = Q share of the contribution of dry mass in organ iii (leaves or roots) for comparison *j*;
- $\text{Share}_{C_{i,m},j}$ = share of the contribution of metabolite concentration mmm (VDL or CAT) in organ *i* (leaves or roots) for comparison *j*;
- $\Delta ln(Y_{tot,m})_j$ = logarithmic variation of $Y_{tot,m}$ for comparison *j*;
- $\left|\Delta ln(Y_{tot,m})_j\right|$ = absolute value of the logarithmic change in the variable $Y_{tot,m}$ for comparison *j*, used as a weight to prevent negative variations from cancelling out positive ones and proportional to the magnitude of the total change

Application to the present study

For VDL or CAT (m), the following is considered:

$$Y_{leaf} = DM_{leaf} \cdot C_{leaf}$$

$$Y_{root} = DM_{root} \cdot C_{root}$$

$$Y_{tot} = Y_{leaf} + Y_{root}$$

Logarithmic variations for each individual comparison j:

$$\Delta\ln(DM_{leaf})_j = \ln\left(DM_{leaf,ref}\right) - \ln\left(DM_{leaf,cond}\right)_j$$

$$\Delta \ln(DM_{root})_j = \ln\left(DM_{root,ref}\right) - \ln\,(DM_{root,cond})_j$$

$$\Delta \ln(C_{leaf})_j = \ln\left(C_{leaf,ref}\right) - \ln\,(C_{leaf,cond})_j$$

$$\Delta \ln(C_{root})_j = \ln\left(C_{root,ref}\right) - \ln\,(C_{root,cond})_j$$

$$\Delta \ln\,(Y_{tot})_j = \ln\left(Y_{tot,ref}\right) - \ln\,(Y_{tot,cond})_j$$

Weighted mean (WM) logarithmic variations:

$$\Delta \ln\left(DM_{leaf}\right)_{WM} = \frac{\sum_{j=1}^{ncomp} \left|\Delta ln(Y_{tot})_j\right| \cdot \Delta ln(DM_{leaf})_j}{\sum_{j=1}^{ncomp} \left|\Delta ln(Y_{tot})_j\right|}$$

$$\Delta \ln(DM_{root})_{WM} = \frac{\sum_{j=1}^{ncomp} \left|\Delta ln(Y_{tot})_j\right| \cdot \Delta ln(DM_{root})_j}{\sum_{j=1}^{ncomp} \left|\Delta ln(Y_{tot})_j\right|}$$

$$\Delta \ln\left(C_{leaf}\right)_{WM} = \frac{\sum_{j=1}^{ncomp} \left|\Delta ln(Y_{tot})_j\right| \cdot \Delta ln(C_{leaf})_j}{\sum_{j=1}^{ncomp} \left|\Delta ln(Y_{tot})_j\right|}$$

$$\Delta \ln(C_{root})_{WM} = \frac{\sum_{j=1}^{ncomp} \left|\Delta ln(Y_{tot})_j\right| \cdot \Delta ln(C_{root})_j}{\sum_{j=1}^{ncomp} \left|\Delta ln(Y_{tot})_j\right|}$$

$$\Delta \ln\left(Y_{tot,m}\right)_{WM} = \frac{\sum_{j=1}^{ncomp} \left|\Delta ln(Y_{tot})_j\right| \cdot \Delta ln(Y_{tot})_j}{\sum_{j=1}^{ncomp} \left|\Delta ln(Y_{tot})_j\right|}$$

Organ-specific LMDI weights

$$\omega_{leaf} = \frac{L(Y_{leaf,ref}, Y_{leaf,cond})}{L(Y_{tot,ref}, Y_{tot,cond})}$$

$$\omega_{root} = \frac{L(Y_{root,ref}, Y_{root,cond})}{L(Y_{tot,ref}, Y_{tot,cond})}$$

The sum of contributions is exact:

$$\Delta \ln(Y_{tot}) = Contr_{DM_{leaf}} + Contr_{DM_{root}} + Contr_{C_{leaf}} + Contr_{C_{root}}$$

Contributions by organ for each individual comparison j:

$$Contr_{DM_{leaf},j} = \omega_{leaf} \cdot \Delta \ln(DM_{leaf})_j$$

$$Contr_{DM_{root},j} = \omega_{root} \cdot \Delta \ln(DM_{root})_j$$

$$Contr_{C_{leaf},j} = \omega_{leaf} \cdot \Delta \ln(C_{leaf})_j$$

$$Contr_{C_{root},j} = \omega_{root} \cdot \Delta\ln(C_{root})_j$$

Weighted mean (WM) physiological contributions

$$Contr_{C_{leaf}WM} = \frac{\sum_{j=1}^{ncomp} |\Delta\ln\,(Y_{tot})_j| \cdot\ Contr_{Cleaf,j}}{\sum_{j=1}^{ncomp} |\Delta\ln\,(Y_{tot,m})_j|}$$

$$Contr_{C_{root}WM} = \frac{\sum_{j=1}^{ncomp} |\Delta\ln\,(Y_{tot})_j| \cdot\ Contr_{Croot,j}}{\sum_{j=1}^{ncomp} |\Delta\ln\,(Y_{tot,m})_j|}$$

$$Contr_{DM_{leaf},WM} = \frac{\sum_{j=1}^{ncomp} |\Delta\ln\,(Y_{tot})_j| \cdot\ Contr_{DMleaf,j}}{\sum_{j=1}^{ncomp} |\Delta\ln\,(Y_{tot,m})_j|}$$

$$Contr_{DM_{root},WM} = \frac{\sum_{j=1}^{ncomp} |\Delta\ln\,(Y_{tot})_j| \cdot\ Contr_{DMroot,j}}{\sum_{j=1}^{ncomp} |\Delta\ln\,(Y_{tot})_j|}$$

$$\Delta\ln(Y_{tot})_{WM} = \frac{\sum_{j=1}^{ncomp} |\Delta\ln\,(Y_{tot})_j| \cdot\ \Delta ln(Y_{tot})_j}{\sum_{j=1}^{ncomp} |\Delta\ln\,(Y_{tot})_j|}$$

Physiological percentage shares per single comparison j:

$$\text{Share}_{DM_{leaf},j}(\%) = \frac{Contr_{DM_{leaf,j}}}{\Delta\ln\,(Y_{tot})_j} \times 100$$

$$\text{Share}_{DM_{root},j}(\%) = \frac{Contr_{DM_{root,j}}}{\Delta\ln\,(Y_{tot})_j} \times 100$$

$$\text{Share}_{C_{leaf,j}}(\%) = \frac{Contr_{C_{leaf,j}}}{\Delta\ln\,(Y_{tot})_j} \times 100$$

$$\text{Share}_{C_{root},j}(\%) = \frac{Contr_{C_{root}},j}{\Delta\ln\,(Y_{tot})_j} \times 100$$

Weighted mean (WM) physiological percentage shares:

$$\text{Share}_{DM_{leaf}WM}(\%) = \frac{\sum_{j=1}^{ncomp} |\Delta ln(Y_{tot})_j| \cdot \text{Share}_{DM_{leaf},j}}{\sum_{j=1}^{ncomp} |\Delta ln(Y_{tot})_j|}$$

$$\text{Share}_{DM_{root},WM}(\%) = \frac{\sum_{j=1}^{ncomp} |\Delta ln(Y_{tot})_j| \cdot \text{Share}_{DM_{root},j}}{\sum_{j=1}^{ncomp} |\Delta ln(Y_{tot})_j|}$$

$$\text{Share}_{C_{leaf},WM}(\%) = \frac{\sum_{j=1}^{ncomp} |\Delta ln(Y_{tot})_j| \cdot \text{Share}_{C_{leaf},j}}{\sum_{j=1}^{ncomp} |\Delta ln(Y_{tot})_j|}$$

$$\text{Share}_{C_{root},WM}(\%) = \frac{\sum_{j=1}^{ncomp} \left|\Delta ln(Y_{tot})_j\right| \cdot \text{Share}_{C_{root},j}}{\sum_{j=1}^{ncomp} \left|\Delta ln(Y_{tot})_j\right|}$$

3. Conceptual and interpretative synthesis

The combined use of simple logarithmic decomposition and the LMDI method makes it possible to distinguish:

• Physiological contributions: effect of biomass and metabolite concentration within each organ

• Structural contributions: effect of the different relative importance of organs (leaf and root) on total dry mass and on total alkaloid yield

• Consistency properties: both decompositions preserve additive exactness and are free of numerical residuals

In biological terms, this approach allows the quantitative and rigorous identification of which physiological or morphological processes (biomass accumulation, metabolite concentration, leaf/root balance) are responsible for the increase in total dry mass and in the total production of VDL and CAT under different experimental conditions.

Conceptual distinction between simple logarithmic decomposition and the LMDI method

It is useful to clarify that simple logarithmic decomposition represents a simplified form of the LMDI (Logarithmic Mean Divisia Index) method, applied to purely multiplicative identities (for example $Y = DM \cdot C$). In the case where the factors are independent and the relationship between variables is strictly multiplicative, simple logarithmic decomposition — based on the logarithmic property $\ln(a \cdot b) = \ln(a) + \ln(b)$ — provides an exact decomposition of the overall logarithmic variation, without the need for weighting.

Conversely, when additive identities are analyzed, the simple logarithmic sum is no longer applicable. In these cases, the LMDI method extends the logarithmic principle through the introduction of weights based on the logarithmic mean L(a,b)L(a,b)L(a,b), which ensure an exact, symmetric, and residual-free decomposition even in the presence of multiple interdependent components.

In theoretical terms, simple logarithmic decomposition can be considered as the simplified form of LMDI in the case of a single multiplicative identity composed of two independent factors.

In such a configuration, the weights ωi\omega_iωi tend to 1 and the LMDI decomposition reduces to the direct logarithmic sum of the contributions of the factors, that is:

$$\Delta Y = \sum_i \Delta \ln (X_i)$$

Therefore:

• simple logarithmic decomposition corresponds to the unweighted version of LMDI, applicable to strictly multiplicative relationships;

• the LMDI method instead represents the generalized and weighted form, necessary to consistently decompose additive identities or more complex structures, preserving numerical exactness and symmetry between comparison conditions.

References

[1] Ang BW. Decomposition analysis for policymaking in energy:: which is the preferred method? Energy Policy 2004;32:1131–9. https://doi.org/10.1016/S0301-4215(03)00076-4.

[2] Su B, Ang BW. Structural decomposition analysis applied to energy and emissions: Some methodological developments. Energy Econ 2012;34:177–88. https://doi.org/10.1016/J.ENECO.2011.10.009.

[3] Ang BW, Liu FL. A new energy decomposition method: perfect in decomposition and consistent in aggregation. Energy 2001;26:537–48. https://doi.org/10.1016/S0360-5442(01)00022-6.

[4] Ang BW, Zhang FQ. A survey of index decomposition analysis in energy and environmental studies. Energy 2000;25:1149–76. https://doi.org/10.1016/S0360-5442(00)00039-6.

[5] Ang BW. LMDI decomposition approach: A guide for implementation. Energy Policy 2015;86:233–8. https://doi.org/10.1016/j.enpol.2015.07.007.

[6] Ang BW. The LMDI approach to decomposition analysis: a practical guide. Energy Policy 2005;33:867–71. https://doi.org/10.1016/J.ENPOL.2003.10.010.

[7] Zhang S, Su X, Singh VP, Ayantobo OO, Xie J. Logarithmic Mean Divisia Index (LMDI) decomposition analysis of changes in agricultural water use: a case study of the middle reaches of the Heihe River basin, China. Agric Water Manag 2018;208:422–30. https://doi.org/10.1016/j.agwat.2018.06.041.

[8] Zhang X, Liu X, Wang L, Zhao Q, Yu Y, Du R, et al. Wheat yield and grain-filling characteristics due to cultivar replacement in the Haihe Plain in China. Front Plant Sci 2024;15:1374453. https://doi.org/10.3389/FPLS.2024.1374453/BIBTEX.

[9] Li T, Baležentis T, Cao L, Zhu J, Kriščiukaitiene I, Melnikiene R. Are the Changes in China's Grain Production Sustainable: Extensive and Intensive Development by the LMDI Approach. Sustainability 2016, Vol 8, Page 1198 2016;8:1198. https://doi.org/10.3390/SU8121198.

[10] Hu J, Zhang J. Spatial–temporal evolution and driving mechanism in soybean production patterns: a case study of Inner Mongolia, China. Front Sustain Food Syst 2025;9:1686721. https://doi.org/10.3389/FSUFS.2025.1686721/BIBTEX.
